# Finding memo: versatile interactions of the VPS10p-Domain receptors in Alzheimer’s disease

**DOI:** 10.1186/s13024-022-00576-2

**Published:** 2022-11-18

**Authors:** Alena Salasova, Giulia Monti, Olav M. Andersen, Anders Nykjaer

**Affiliations:** The Danish National Research Foundation Center PROMEMO, The Danish Research Institute of Translational Neuroscience (DANDRITE), Nordic-EMBL Partnership for Molecular Medicine, Department of Biomedicine, Aarhus University, Høegh-Guldbergs Gade 10, DK-8000 Aarhus, Denmark

**Keywords:** Alzheimer’s disease, SorLA, Sortilin, SorCS1, SorCS2, SorCS3, Neurotrophins, Comorbidity

## Abstract

The family of VPS10p-Domain (D) receptors comprises five members named SorLA, Sortilin, SorCS1, SorCS2 and SorCS3. While their physiological roles remain incompletely resolved, they have been recognized for their signaling engagements and trafficking abilities, navigating a number of molecules between endosome, Golgi compartments, and the cell surface. Strikingly, recent studies connected all the VPS10p-D receptors to Alzheimer’s disease (AD) development. In addition, they have been also associated with diseases comorbid with AD such as diabetes mellitus and major depressive disorder. This systematic review elaborates on genetic, functional, and mechanistic insights into how dysfunction in VPS10p-D receptors may contribute to AD etiology, AD onset diversity, and AD comorbidities. Starting with their functions in controlling cellular trafficking of amyloid precursor protein and the metabolism of the amyloid beta peptide, we present and exemplify how these receptors, despite being structurally similar, regulate various and distinct cellular events involved in AD. This includes a plethora of signaling crosstalks that impact on neuronal survival, neuronal wiring, neuronal polarity, and synaptic plasticity. Signaling activities of the VPS10p-D receptors are especially linked, but not limited to, the regulation of neuronal fitness and apoptosis via their physical interaction with pro- and mature neurotrophins and their receptors. By compiling the functional versatility of VPS10p-D receptors and their interactions with AD-related pathways, we aim to further propel the AD research towards VPS10p-D receptor family, knowledge that may lead to new diagnostic markers and therapeutic strategies for AD patients.

## Background

### Alzheimer’s disease pathophysiology

Over 55 million people worldwide suffer from dementia, which is expected to rise to 78 million by 2030 (*World Alzheimer Report 2021*). Alzheimer’s disease (AD) accounts for 60–80% of all diagnosed dementia cases [1]. Disturbingly, no efficient treatment is currently available. This unmet medical need is likely a consequence of the complex biology of the disease, which remains poorly understood. AD is clinically characterized by extensive neuronal cell death in the cerebral cortex and limbic system that is manifested by cognitive impairments, memory deficits, disorientation, spatiovisual difficulties, linguistic problems, and emotional imbalances. At the histopathological levels, AD is defined by the accumulation of extracellular Amyloid-β (Aβ) plaques and by the formation of intracellular neurofibrillary tangles composed of hyperphosphorylated Tau protein (pTau) in the brain parenchyma [2]. The Aβ plaques are considered the pathological hallmark of AD, but synergistic effect of pTau leading to weakening and deterioration of synapses, dystrophic neurites, neuroinflammation, and progressive neuronal cell death is likely involved too [2, 3].

**Fig. 1:**
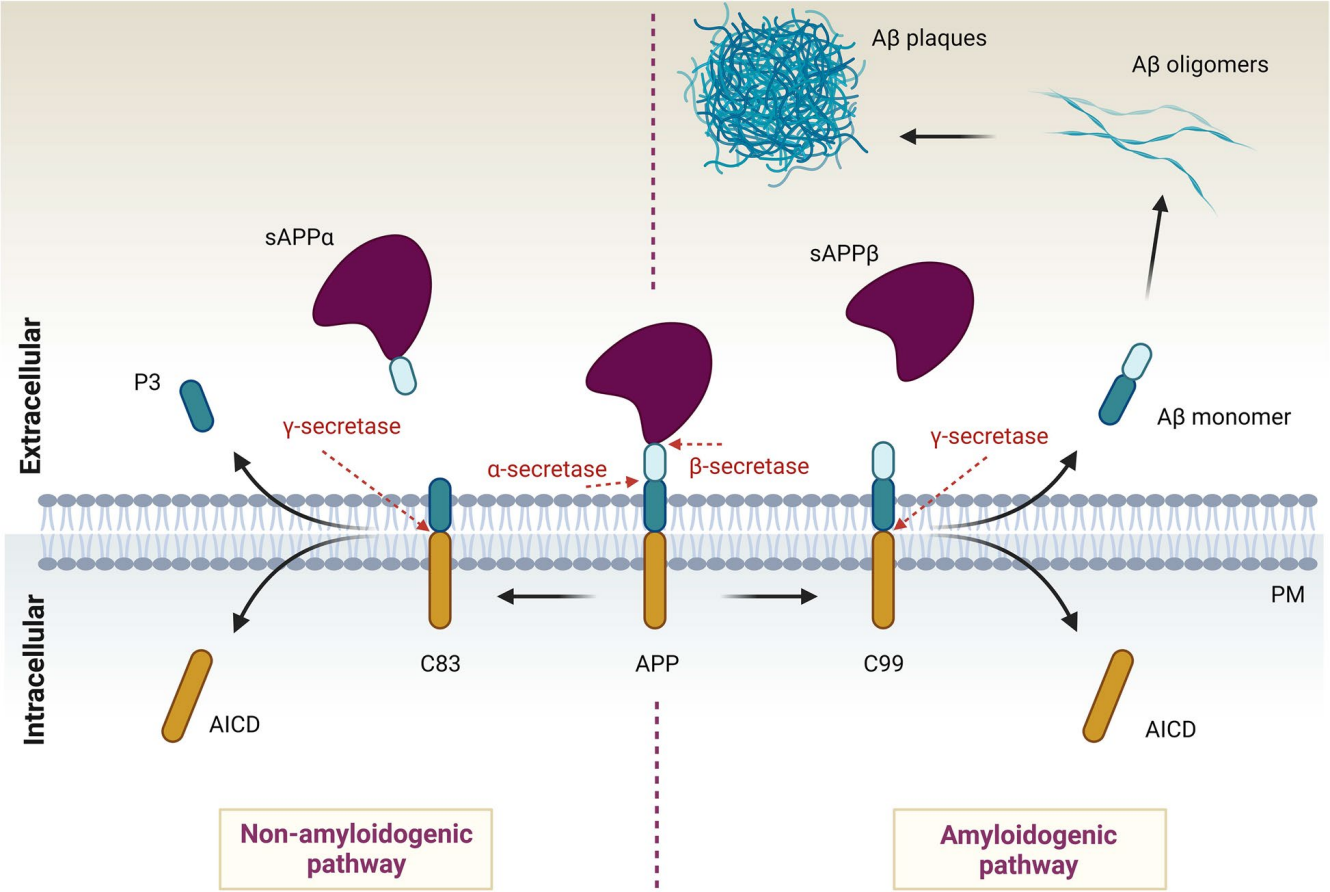
A simplified scheme of APP proteolytic processing and the origin of Aβ plaques. APP is a type I transmembrane receptor that contains Aβ peptide within its sequence. α-secretases such as ADAM10/17 cleave APP inside the Aβ peptide, which is disrupting, and produces soluble, secreted sAPPα fragment. sAPPα is neuroprotective, and thus this cleavage is called non-amyloidogenic pathway. The C83 peptide can be further cleaved by γ-secretase producing soluble P3 peptide. In contrast, APP can be cleaved by β-secretase, for example BACE1, creating a cytotoxic, soluble sAPPβ. The proteolysis by β-secretase exposes Aβ peptide, which is further cleaved by γ-secretase. This cleavage results in the release of Aβ monomers into the extracellular space, where they can further polymerize forming Aβ oligomers, and later Aβ plaques. This pathway is neurotoxic and is called amyloidogenic pathway

The Aβ peptide lies within the amyloid precursor protein (APP). Under normal conditions, the nascent APP is transported through the trans-Golgi network (TGN) to the plasma membrane where it is sequentially cleaved by α- and γ-secretases, respectively, disrupting the Aβ sequence. These processes produce and liberate the non-pathological, soluble fragment called sAPPα. The APP processing is depicted in (Fig. 1). According to the “amyloid cascade hypothesis”, APP may, however, escape α-secretase cleavage. Following internalization to the endosomal compartment, APP is then sequentially processed by β-secretase and γ-secretase, respectively, which generates the Aβ peptide. After secretion into the extracellular space, Aβ peptides polymerize into detrimental Aβ oligomers (AβO), later forming the ultimately insoluble, cytotoxic Aβ plaques [4]. However, attempts to treat AD by lowering Aβ have failed in several clinical trials which questions the impact of Aβ plaques as the only factor that controls disease progression [5]. Imbalance between proapoptotic and neuroprotective stimulation by proneurotrophins and mature neurotrophins likely contributes to the AD pathophysiology by negatively impacting on synaptic plasticity, and neuronal vulnerability and integrity [6–10]. Strikingly, the family of Vacuolar protein sorting 10p-Domain (VPS10p-D) receptors plays a dual role in the pathobiology of AD: it controls APP and Aβ trafficking and clearance, and it regulates the balance between the trophic and apoptotic signaling by neurotrophins such as BDNF and its precursor proBDNF. These activities may explain why members of this receptor family have surfaced as important risk genes in AD. Interestingly, the individual receptors often regulate different, sometimes even opposing aspects of AD, which will be discussed mostly in the main text.

### Genetic risks in AD

The strongest genetic evidence behind AD pathogenesis is linked to familial early-onset AD (EOAD), which accounts for 5–10% of all AD cases. EOAD diagnosis has been mostly associated to autosomal-dominant mutations in three genes that result in increased levels and aggregation of Aβ. They are *APP*, and APP-cleaving γ-secretase presenilin components, *PSEN1* and *PSEN2*. However, the majority of patients are diagnosed with sporadic, late-onset AD (LOAD), the heritability of which is estimated to be between 60 and 80% [11, 12]. Hence, genome-wide association studies (GWAS) investigating up to 150,000 AD cases have identified a number of risk genes underscoring the polygenic nature of LOAD [11, 13, 14]. Most recently, a study incorporating more than 100,000 AD cases and almost 700,000 control individuals identified 75 risk loci, of which 42 had not previously been described [14]. Functional annotation of the risk genes indicated that amyloid aggregation, Tau phosphorylation, endocytosis, intracellular vesicle trafficking, altered lipid metabolism, and immune responses are critically involved in the pathogenesis of LOAD [2, 13–18]. Notably, around 40% of LOAD patients carry a disease-associated SNP in the gene encoding Apolipoprotein E (*APOE*), which exists in 3 polymorphic alleles called E2, E3, and E4. Due to such high incidence, *APOE* polymorphism is considered the most important genetic risk factor for LOAD [19–21]. ApoE has multiple physiological functions, but it is mostly known for transportation of cholesterol and other lipids through the circulation system as well as within the brain parenchyma. Even though all human ApoE isoforms interact with Aβ, the functional outcome is different [22]. While ApoE2 shows neuroprotective features, ApoE4 represents the major risk factor for AD due to its involvement in Aβ processing [23]. The possession of ApoE4 allele leads to intracellular accumulation of Aβ by enhancing the uptake of Aβ peptides, resulting in the enlargement of endosomal compartments, subsequent endosomal-lysosomal pathway dysregulation, and thus decreased clearance of Aβ [24]. Several other mechanisms have been described for ApoE4 in relation to AD, including neuronal hyperactivation [25], increased Tau phosphorylation [26], modulation of neuroinflammatory pathways [23], and impaired synaptic plasticity [27]. Such abnormalities, fueled by reduced trophic support, are considered major drivers behind the progression of AD. For this reason, impairments in proteins involved in endocytic trafficking and trophic signaling have surfaced as important AD risk factors including the VPS10p-D receptors family.

### AD as imbalance between neurodegeneration and neuroprotection

The neurotrophin protein family (NTs) is a subgroup of secreted neurotrophic factors that are essential for axonal outgrowth, neuronal differentiation, synaptic plasticity, and neuronal survival [9, 28, 29]. It comprises Brain-derived neurotrophic factor (BDNF), Nerve growth factor (NGF), Neurotrophin 3 (NT3), and Neurotrophin 4 (NT4); proteins that are expressed across the CNS in a spatiotemporal manner. Their actions depend on binding to transmembrane receptor complexes composed of Tropomyosin receptor tyrosine kinase (Trk) that is ligand-specific, and the promiscuous neurotrophin receptor denoted p75 (p75^NTR^). While all neurotrophins can bind p75^NTR^, they show strongest binding to their respective Trk receptor: NGF binds to TrkA, BDNF and NT4 binds to TrkB, and NT3 bind to TrkC. The p75^NTR^ can form heterodimers with a given Trk, which increases the affinity and fidelity of the Trk receptor towards its cognate neurotrophin [9].

BDNF is considered particularly relevant to AD [8, 9]. Reduced BDNF expression in the hippocampus and cortex of AD patients have been consistently reported at both transcriptional and protein levels [30–33]. In addition to its well established function in sustaining neuronal survival, BDNF is also important for cognitive abilities as it promotes learning and memory by increasing synaptic strength [8, 34, 35]. There is a naturally occurring single nucleotide polymorphism (SNP) in BDNF at codon 66, which results in the substitution of Valine with Methionine (BDNF-Val66Met). This mutation has been associated with reduced synaptic plasticity, dendritic spine elimination [36], and impaired memory and learning in AD patients [37–39]. Strikingly, in rodent and primate models of Alzheimer’s disease, BDNF gene delivery administered after disease onset showed potent neuroprotective effects by increasing synaptogenesis and synaptic plasticity leading to restoration of cognitive function [40]. Likewise, transplantation of neural stem cells have been able to rescue memory function in AD mice via BDNF-induced stimulation of synaptogenesis [41].

BDNF levels also shape the onset of AD neurodegeneration by regulating Aβ production and formation of Tau containing neuritic plaques and neurofibrillary tangles [34]. In cultured hippocampal neurons, BDNF deprivation leads to increased cell death by 50% and elevated levels of APP and PSEN1, all of which can be rescued by inhibiting Aβ production [42]. Low BDNF levels increase expression of δ-secretase that cleaves Tau to produce a pathogenic fragment [43]. Subsequently, Tau peptide becomes hyperphosphorylated, abolishing microtubule assembly, and triggering the formation of neurofibrillary tangles. Tau peptide also binds TrkB leading to degradation of the receptor. This prevents trophic activity of TrkB and blunts APP phosphorylation thereby increasing Aβ production [44, 45]. The importance of BDNF is further substantiated by the beneficial effects of physical exercise, which increases BDNF and TrkB expression, reduces APP processing and amyloid aggregation, and protects against cognitive decline in animal models and AD patients. Indeed, regular physical activity is considered among the most efficient ways to slow AD progression today [46–49]. Taken together, the above findings suggest that increasing BDNF can have therapeutic benefits for AD patients.

NTs are synthesized as precursor proteins named “proneurotrophins” (proNTs) that undergo proteolytic cleavage of the prodomain during their maturation [50, 51]. proNTs are active signaling molecules, but as opposed to their mature counterparts, they induce apoptosis, axonal growth cone retraction, and synaptic weakening; actions that require p75^NTR^ and are independent of Trk [50, 52–56]. As a consequence, perturbed maturation and incorrect balance between proNTs and NTs may propel the neurodegenerative process and exacerbate the disease [33, 51, 57]. Indeed, AD patients with mild to medium cognitive impairments commonly exhibit increased levels of proBDNF in cortex, hippocampus and cerebrospinal fluid (CSF) on the expense of reduced mature BDNF [40, 57–61]. The studies showed that while proNGF is increased, NGF is decreased in the CSF and in different brain regions. Notably, this is the case in the basal forebrain where cholinergic neurons are reliant on trophic stimulation from NGF while being sensitive to proNGF-induced apoptosis [59, 62–67]. Expression of the neurotrophin receptors are altered too. Hence, p75^NTR^ is commonly upregulated in several regions affected in AD brains [68–73], whereas TrkA [59, 74–76], TrkB [77], and TrkC [77] are downregulated. Reported changes in neurotrophin signaling components in AD patients are summarized in (Table 1).

**Table 1:** Reported changes in transcriptional and/or protein levels of neurotrophin signaling components exhibited by AD patients

***NEUROTROPHIN SIGNALING***	***EXPRESSION LEVELS IN AD PATIENTS***	
	**Upregulation**	**Downregulation**
proNGF	↑ in brain [59, 62–64]; ↑ in CSF [65]	
NGF	↑ in brain [68]	
proBDNF		↓ in brain [59, 60]
BDNF		↓ in brain [59, 60]
p75NTR	↑ in brain [68–71]; ↑ in serum [72, 73]	↓ in CSF [72, 73]
TRKA		↓ in brain [59, 74–77]
TRKB		↓ in brain [77]
TRKC		↓ in brain [77]

A functional interaction between APP/Aβ metabolism and neurotrophin system has also been demonstrated. p75^NTR^ can bind APP to enable APP trafficking to the endosomal compartment. Hence, in AD mouse models, removal of p75^NTR^ or disruption of its internalization substantially lowers amyloidogenic processing, Aβ levels, and reduces cognitive decline [78, 79]. p75^NTR^ also promotes amyloid-induced neuritic dystrophy [80], while soluble ectodomain of p75^NTR^ is neuroprotective against Aβ [73]. Phosphorylation of APP promotes amyloid processing of APP along the amyloidogenic pathway. NGF induces the binding between TrkA and APP, which downregulates APP phosphorylation, and enables its retrograde trafficking into the TGN thereby bypassing β-secretase processing [81, 82]. Taken together, the imbalance between proNTs and NTs, and the expression levels of their receptors may shift their function from being neuroprotective to amplifying neurodegenerative processes.

### Introduction to VPS10p-D receptor family

**Fig. 2:**
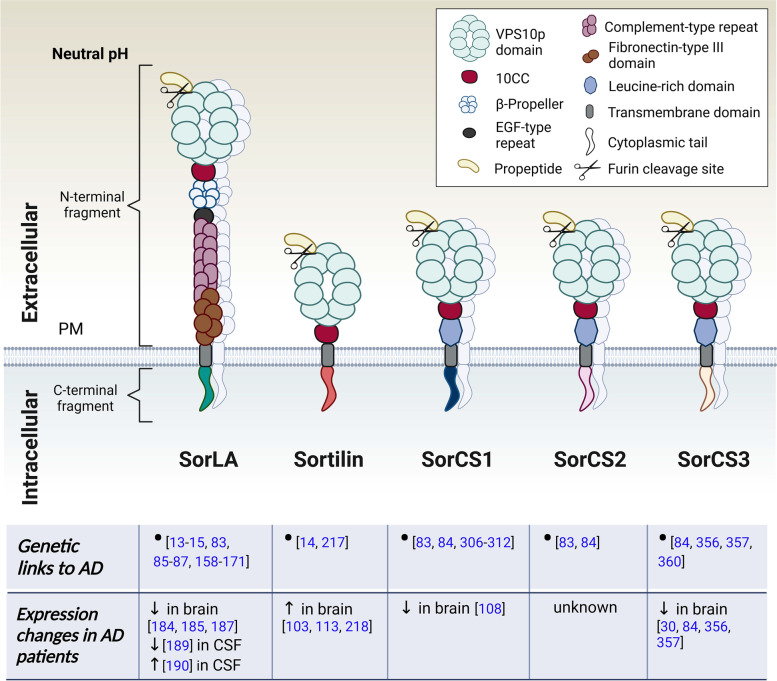
VPS10p-D receptors – their structure, and genetic and transcriptional relations to AD. The VPS10-D receptors are produced as proforms containing a propeptide which is cleaved by Furin. Except for SorLA, the receptors exhibit similar structure, mostly differing in the sequence of their intracellular cytoplasmic tails. SorLA can dimerize at neutral pH while Sortilin forms dimers only at acidic pH, for example in lysosomes. SorCS1-3 are paralogs that tend to form stable homodimers. All the receptors have been genetically linked to AD. Independently from the genetic background, AD patients display changes in the receptors’ expression levels within the brain parenchyma. These are predominantly diminished, and likely contribute to the disease progression such as decreased neuroprotection

VPS10p-D receptor family comprises five single-span type 1 transmembrane receptors SorLA, Sortilin, SorCS1, SorCS2, and SorCS3. All members of this receptor family have been recently identified as AD risk loci, and are now considered hotspots in LOAD [14, 83, 84]. Notably, SorLA has been also associated with familial EOAD [85–87]. Accordingly, based on several GWAS studies, VPS10p-D receptors exhibit key functions in the causal pathways influencing Alzheimer’s disease risk such as APP catabolic processes, cholesterol and lipid metabolism, endocytosis, cellular sorting and trafficking, and immune responses [2, 13–18]. They are involved in the etiology of a number of neurological and psychiatric disorders [88–90] including AD and frontotemporal dementia [84, 91–94]. Strikingly, genetic variants in all VPS10p-D receptors have been associated with AD, with their expression being predominantly decreased in the diseased brains. We present an overview of the structure and genetic association of the VPS10p-D receptors with AD in (Fig. 2). Except for SorCS2, the receptors functionally interact with a number of canonical AD proteins including APP, Aβ, secretases, and ApoE. We summarized the functional interactions of the receptors with AD-related proteins in (Table 2).

**Table 2:** Reported functional interactions of VPS10p-D receptors with canonical AD-related proteins

***AD-RELATED PROTEINS***	***VPS10p-D RECEPTORS***				
	**SorLA**	**Sortilin**	**SorCS1**	**SorCS2**	**SorCS3**
*APP*	• [95–100] x [101, 102]	• [97, 103–105]	• [106–108] x [101]		• [84] x [101]
*Amyloid β*	• [109–111]	• [93, 104, 112] x [113]	x [106, 108]		
β-secretase *BACE1*	• [114]	• [97, 103, 105]			
γ-secretases *(PSEN1/2)*	• [91, 115]	• [91]	• [91, 115]		• [84, 115]
*APOE*	• [110, 116]	• [93]			
*α-secretases (ADAM10, ADAM17)*		• [91, 115, 117, 118]	• [91]		

• - Functional interaction

x - Transcriptional changes (indirect interaction)

Expression of VPS10p-D receptors is regulated in spatiotemporal manner from embryonic development to adulthood [119–123], with SorCS1-3 being particularly sensitive to external stimuli [124–127]. In adult brain, SorCS1-3 expression predominates in neurons, whereas Sortilin and SorLA are also expressed by immune cells such as microglia/macrophages or T and B lymphocytes [128]. For more insights regarding the cell-type specific expression of VPS10p-D receptors please explore e.g. *Brainseq.org* database. SorLA, SorCS1, and SorCS2 exist in more splice variants and cleavage products, but they can also exist in soluble forms that can modify cell signaling over long distances (so far shown for SorLA and Sortilin). These features broaden molecular interactions and signaling diversity of VPS10p-D receptors across distinct cell types [129–131]. VPS10p-D receptors act by two different signaling modalities; either they control signal transduction at the cell membrane where they bind their ligands and co-receptors, or they sort multiple types of cargoes by endocytosis and intracellular trafficking thus targeting them to distinct cellular compartments. Dysfunction in endosomal and lysosomal pathways typically causes cytotoxic protein aggregation and altered cell signaling, a major cause behind the progression of AD neurodegeneration. Importantly, VPS10p-D receptors also bind synaptic components, in particular pro- and mature neurotrophins and their respective receptors, to control synaptic plasticity, synaptogenesis and cell fate decisions. These processes are also severely affected in AD [52, 132, 133]. The reported functional interactions between the VPS10p-D receptors, neurotrophins and their receptors are listed in (Table 3).

**Table 3:** Reported functional interactions of VPS10p-D receptors with components of neurotrophin signaling

***NEUROTROPHIN SIGNALING***	***VPS10p-D RECEPTORS***				
	**SorLA**	**Sortilin**	**SorCS1**	**SorCS2**	**SorCS3**
proNGF		• [52, 53, 134]		• [55, 132, 135]	
NGF		• [52]		• [135]	• [136]
proBDNF		• [134] x [113]		• [54, 132, 135]	
BDNF	x [137]	• [117, 138]		• [36, 54, 139]	
proNT3		• [140, 141]			
p75NTR		• [52, 53, 134] x [113]		• [55, 132]	
TrkA		• [142]			
TrkB	• [143]	• [142]	• [144]	• [54]	• [144]
TrkC		• [142]			

• - Functional interaction

x - Transcriptional changes (indirect interaction)

The VPS10p-D receptor family is named after the yeast sorting protein VPS10p-D that forms their N terminus. A schematic representation of the receptors’ structure is illustrated in (Fig. 2). All the receptors contain an extracellular propeptide typically removed by Furin-mediated cleavage in the late-Golgi compartments within the secretory pathway (Fig. 2). This proteolytic event is considered a stringent requirement for receptor activation, since it primes the VPS10p-D domain for ligand binding. So far, this has been demonstrated for SorLA and Sortilin [145–147]. The propeptide is followed by the archetype VPS10p-D domain, which is a 10-bladed β-propeller initially identified in the yeast S. cerevisiae, and that serves as binding site for many target proteins [148]. All VPS10p-D receptors can undergo ectodomain shedding to a different extend, thus releasing a soluble fragment into the extracellular space [91, 115, 149–151].

Structurally, the receptors differ from each other also by the unique sequences within their cytoplasmic domains, which determine their distinct signaling features and sorting capacities due to diverse interactions with adaptor molecules. However, given that some adaptor molecules can interact with more than one VPS10p-D receptor, and that several ligands are shared between the receptors, the VPS10p-D receptors sometimes exhibit redundant or complementary functions [133, 148]. VPS10p-D proteins can form homodimers or heterodimers with each other [152], as well as engage in formation of receptor complexes with other transmembrane proteins including APP, p75^NTR^, TrkA and TrkB [153].

Importantly, the receptors differ also by their preferred oligomeric states and conformational dynamics. For example, Sortilin exists only as a monomer at neutral pH, and dimerizes selectively in acidic environment [154, 155]. On the other hand, SorCS1-3 prefer forming stable dimers at neutral pH (by 79% for SorCS3) [135, 152, 156], which is likely determined by posttranslational modifications of monomers such as glycosylation levels [152]. So far three different dimeric conformations have been identified for SorCS3 [156]. Interestingly, SorLa seems to have an even preference for monomeric (by 52%) vs dimeric (by 48%) assembly at neutral pH, while the bound within the dimer is less stable [157]. Unfortunately, conformational states of SorCS1-3 and SorLA at acidic pH have not been resolved yet. Similarly, we still lack complete crystal structures of the receptors determining their intracellular tails or ligand-dependent conformational changes to obtain complex insights into their structural features.

Put together, oligomerization diversity in different subcellular compartments together with the precise composition of their protein complexes determine signaling and sorting actions of the VPS10p-D receptors which span from e.g. APP processing and Aβ turnover to cell survival and proapoptotic cell behavior. Moreover, the distinct expression profiles, various protein interactions, and high mobility of the VPS10p-D receptors highlight their pleiotropic function in neuronal trafficking and cell-to-cell communication in developing CNS as well as in adult brain [121, 122, 132]. A few studies suggested synergistic activity of these receptors, likely due to their structural similarities and overlapping ligands when expressed in the same tissue, i.e. in the hippocampus [121, 152]. This may explain why their shared heritability can result in epistatic effects in developing AD [84]. Here we present an overview of the many joint but also distinct roles played by the VPS10p-D receptors in neuronal communication, with a particular focus on their contribution to AD pathogenesis.

## Main text

### SorLA biology and its role in AD

The strongest genetic link to AD occurs for the *SORL1* gene that has been included in the exclusive list of genes that can act as causal for AD (together with *PSEN1*, *PSEN2,* and *APP* [2]). Initially found in LOAD cohorts [83, 158–164], rare *SORL1* variants were recently identified also in EOAD patients [85–87, 165–169], and in cases with familial AD [85, 170, 171]. Its protein product, sorting-related receptor with A-type repeats (SorLA, also known as LR11), is the largest member of the VPS10p-D receptor family. It was first identified in 1996 as a non-canonical member of the low-density lipoprotein receptor family that, in addition to the VPS10p-D, also contained 11 complement-type repeats, one EGF-type repeats, and six fibronectin type III domains [116, 172, 173]. A recent study by Zhang et al. used a single-particle cryogenic electron microscopy to determine that SorLA can exist as a monomer (52% of the particles) as well as a dimer (48% of the particles) at neutral pH. Unfortunately, the authors failed to explore conformations at acidic pH and contribution of the C-terminus to dimerization [157].

SorLA is found in most regions of the mammalian CNS with a predominant neuronal expression. It is mostly localized in endosomal sorting compartments, where it mediates the trafficking of variety of cargo molecules such as APP [95], β-secretase 1 (BACE1) [114], Aβ [109], or Glial cell line-derived neurotrophic factor (GDNF) [174]. The extensive role of SorLA in controlling protein sorting is defined by cytosolic adaptors that recognize specific motifs located within the receptor tail. Once at the cell surface, SorLA can be internalized via a clathrin-dependent mechanism through binding to the clathrin adaptor protein 2 (AP-2) [175]. Internalized SorLA molecules are trafficked from endosomal compartments either directly back to the plasma membrane, which is dependent on its interaction with retromer and SNX-27 [176, 177], or to the TGN by retrograde sorting facilitated by cytosolic adaptors PACS1 and the retromer complex containing VPS26 [177, 178]. From the TGN, SorLA can return to the endosomes by anterograde transport guided by adaptors Golgi-localized γ-ear-containing Arf-binding proteins GGA1 and GGA2 [179] and AP-1 [175]. Jointly, these trafficking events establish an endosome-TGN shuttle. Interestingly, AP-1, a key regulator of dendritic targeting of transmembrane receptors [180], was also shown to facilitate the polarized transport of SorLA towards somatodendritic domains in primary cultures of rat hippocampal neurons [181]. Recently, the heat shock protein 12A (HSP12A) was identified as a novel adaptor molecule able to selectively target the cytoplasmic domain of SorLA in an ATP-dependent fashion, affecting its internalization and subcellular localization [182]. Also, the last three amino acids “VIA” at the C terminus of SorLA mediates binding to Protein Interacting with C Kinase-1 (PICK1), a protein known to promote transport of different cargos to endosomes including postsynaptic AMPA glutamate receptor (AMPAR) [183].

Recently, an alternatively spliced *SORL1* variant was identified and designated “*SORL1-38b*” due to the inclusion of an additional exon 38b. SorLA-38b lacks four fibronectin domains, the transmembrane domain, and the cytoplasmic tail giving rise to a truncated and soluble receptor [184]. As opposed to the full-length receptor, which is enriched in the soma, SorLA-38b predominates in dendrites, suggesting it may assist synaptic functions in a manner that does not require receptor trafficking. Notably, the number of *SORL1-38b* transcripts is substantially decreased in the cerebellum of AD patients, whereas the expression of full-length receptor is unaltered. The protein truncation at the C-terminus disables SorLA interaction with cytosolic adaptors, which is likely involved in distinct biological processes than the full-length receptor [184].

**Fig. 3:**
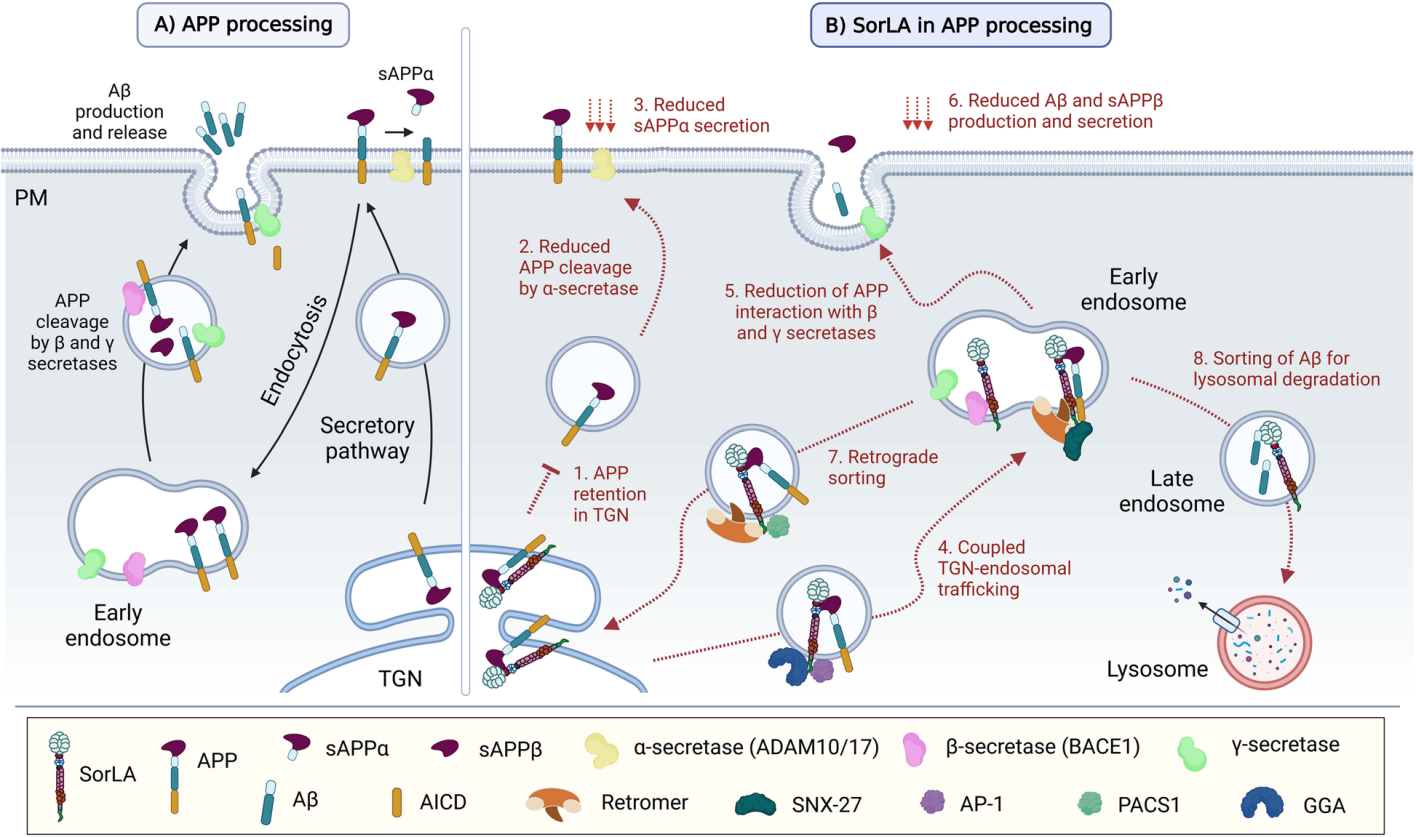
The role of SorLA in APP processing. **A** APP is directed from the trans-Golgi network (TGN) to the plasma membrane via the secretory pathway. APP molecules are either cleaved by α-secretase at the plasma membrane or recycled through endocytosis, and trafficked by early endosomes. There, APP is sequentially cleaved by β- and γ-secretases, thus generating Aβ monomers that are secreted to the extracellular space. **B** A model of SorLA involvement in the amyloid cascade. *1.-3.* SorLA interacts with APP in TGN acting as a retention factor, which reduces α-secretase cleavage and secretion of sAPPα from the cell surface. *4.-7.* In addition, SorLA forms a complex with APP that shuttles between the TGN and endosomes. The anterograde transport is dependent on SorLA’s interaction with AP-1 and GGA (*4.*), while the retrograde transport is determined by its binding to retromer or PACS1 (*7.*). SorLA’s interaction with retromer and SNX-27 in early endosomes additionally enables the sorting of APP along the recycling pathway to the plasma membrane (*5.*). This way, the sorting receptor is responsible for reducing the interaction between APP and β- and γ- secretases (*5.*). Importantly, binding of SorLA to BACE1 blocks the APP-BACE1 interaction, which reduces the production of secreted Aβ peptides (*6.*). Last but not least, SorLA also engages with Aβ peptides in endosomes and navigates them for the lysosomal degradation (*8.*)

**Fig. 4:**
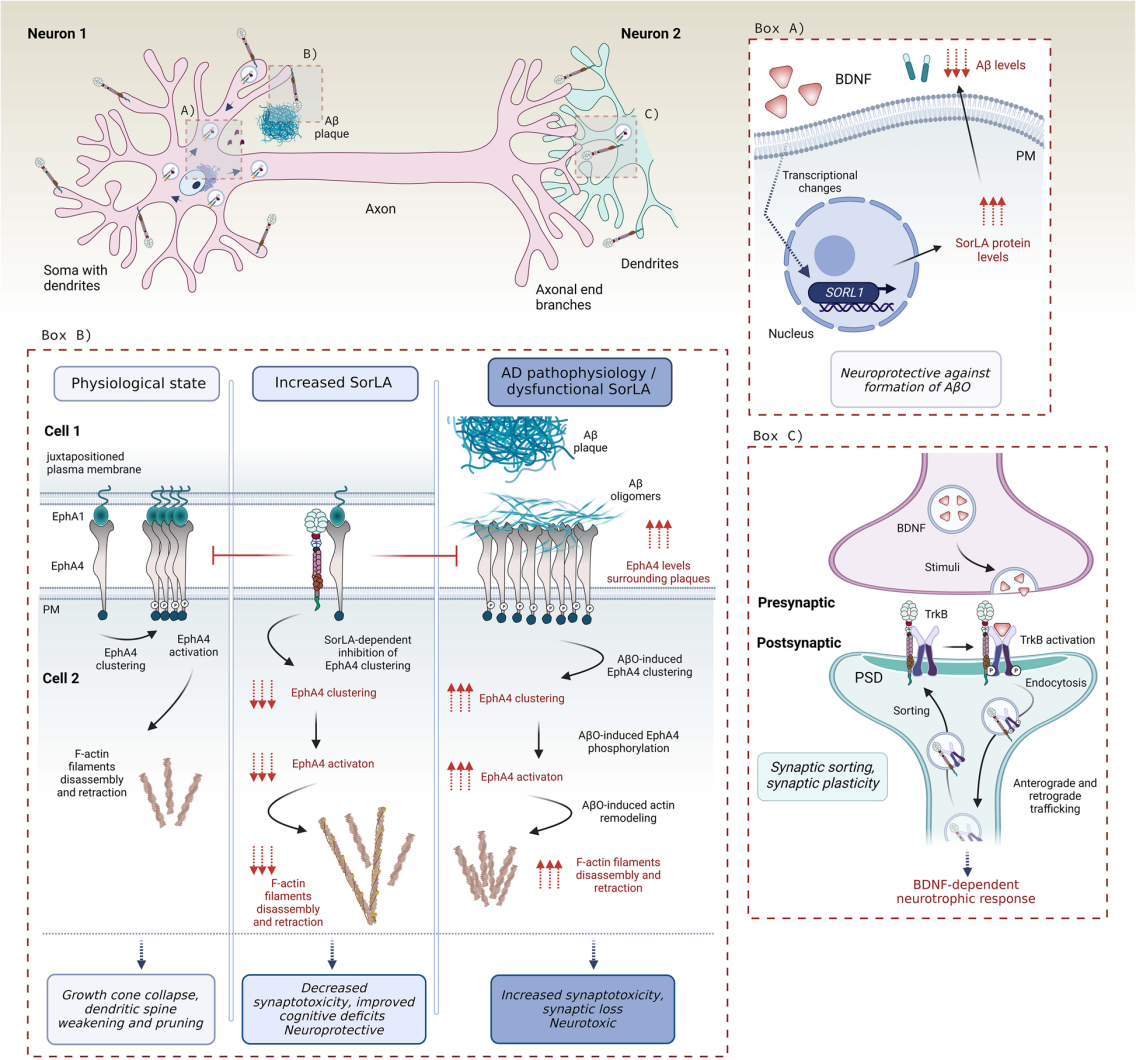
SorLA localization within a neuron and its signaling in AD. SorLA predominantly localizes in neural soma and dendrites, either in sorting vesicles or at the plasma membrane. Box A) The presence of extracellular BDNF in human brain mediates expression of *SORL1*, which increases SORLA protein levels attenuating the production and secretion of Aβ. Box B) A scheme of how SorLA regulates EphA4 signaling. Under physiological conditions (*left panel*), EphA4 binds its juxtapositioned ligand EphA1 which triggers clustering of EphA4 receptors, and their subsequent phosphorylation. EphA4 activation triggers disassembly and retraction of F-actin filaments causing growth cone collapse crucial e.g. for dendritic spine pruning. AD patients (*right panel*) show increased levels of EphA4 in close proximity to Aβ plaques. Moreover, EphA4 binds Aβ oligomers which results in increased AphA4 activation and abnormal actin filaments retraction causing dendritic spine retraction and synaptic loss. SorLA (*middle panel*) binds EphA4, which prevents EphA4 clustering. Increased SorLA levels thus diminish the EphA4 activation, which lowers the responsiveness of the neurons to growth cone retraction even in presence of AβO, thus protecting the neurons against synaptotoxicity. Box C) SorLA binds and traffics TrkB receptor towards the synapse where they remain as a receptor complex. Upon BDNF release and subsequent activation of TrkB, SorLA further drives TrkB internalization, which is a critical step for the subsequent BDNF-dependent neurotrophic response and synaptic plasticity

SorLA also interacts with transmembrane receptors at the synapse such as TrkB [143], GDNF receptors GFRα1 and RET [174], and EphA4 [96] in order to modulate neuronal integrity and synaptic plasticity events. It is worth noticing that SorLA undergoes differential trafficking and polarized distribution, which subsequently influences axonal or dendritic guidance of its cargos [181]. Strikingly, *SORL1* transcription can be enhanced by BDNF leading to an increased production of SorLA, which has a neuroprotective effect, and also reduces the production of Aβ levels [137]. In addition to its intracellular functions, the ectodomain of SorLA can be liberated by ADAM17 and released into the extracellular space as soluble receptor (sSorLA) [115]. It was shown that sSorLA binds and activates EGF receptor to induce neurite outgrowth and neurite regeneration [150]. In the following paragraphs we will describe molecular interactions of SorLA with AD-related pathways and its role in neuroprotection. A schematic representation of SorLA functions is shown in (Figs. 3 and 4).

#### SorLA interactions in amyloidogenic cascade

While uncovering differences in gene expression between AD and control brains, Scherzer et al. discovered that *SORL1* expression is dramatically reduced in hippocampus and frontal cortex of patients with sporadic AD [185, 186]. Soon after, SorLA was identified as an interaction partner for APP that determines APP intracellular trafficking and processing (Fig. 3) [95]. SorLA activity influences APP metabolism by retaining APP in the TGN, and by escorting APP out of endosomes thereby preventing proteolytic cleavages of APP into Aβ peptide in endosomal compartments [95]. SorLA also regulates polarized distribution of APP within a neuron [97, 181]. Kinetic studies from Schmidt et al. revealed that the receptor is able to inhibit oligomeric assembly of APP both in vitro and in vivo, which influences its processing as the APP dimer is a preferred substrate of its secretases [98]. These observations were confirmed using a SorLA deficient mouse line that exhibits increased production of Aβ peptides in the brain parenchyma [187]. AD mouse models APP/PSEN1 and PDAPP further revealed that cerebral levels of Aβ peptides and the deposition of plaques were significantly exacerbated in a SorLA concentration-dependent manner [187, 188]. Interestingly, overexpressed SorLA mediates an increased uptake of sAPP from the medium [97]. It is worth noticing that both, reduced levels of soluable SorLA [189] as well increased levels of soluable SorLA [190] have been detected in CSF of AD patients. Altogether, these findings propose that SorLA may critically influence amyloidogenic events underlying the AD pathology and nominate the receptor as a potential target for the therapeutic interventions.

The physical interaction of SorLA with APP occurs within the complement-type repeats cluster of SorLA. The two proteins form a 1:1 stoichiometric complex observed more efficiently at acidic rather than neutral pH. This finding fosters a model of SorLA and APP interacting inside of the secretory and endosomal vesicles where the luminal pH is maintained in a range of 5.5 – 6.5, as opposed to the neutral pH at the cell surface [99]. Maturation of APP O-glycans in the Golgi compartment is required for the APP precursor release into the secretory pathway, determining how APP cleavage is regulated, thus affecting the formation of its soluble forms (sAPPα and sAPPβ) [191, 192]. Interestingly, engineered SorLA mutants were found to influence the APP breakdown by regulating its O-glycosylation, which exemplifies yet another mechanism how this receptor interferes with amyloidogenesis [99]. Furthermore, a splice variant of *SORL1* that incorporates a novel exon 30B and denoted *SORL1-30B*, is present in most brain regions with the highest expression in the temporal lobe and the hippocampus [193]. The receptor variant specifically lacks the binding sites for Aβ and APP, leaving the rest of the receptor intact. This observation suggests that alternative transcription may provide SorLA the ability to control the ligand specificity.

Besides APP trafficking and processing, SorLA controls additional events in the amyloidogenic cascade. Overexpression studies by Spoelgen et al. showed that SorLA’s cytoplasmic tail forms a protein complex with BACE1, a secretase initiating the proteolysis of APP. As SorLA interacts with both BACE1 and APP, the authors proposed that SorLA can render APP less accessible to the secretase, by which it prevents the formation of the BACE1-APP complex in the endosome, thus reducing the APP cleavage. SorLA-BACE1 interaction therefore directly affects APP processing and Aβ production [114]. Importantly, the secreted form of the receptor, sSorLA, is released into CSF, which was found to positively correlate with sAPPβ and Tau in AD patients [194].

SorLA has been also demonstrated to bind ApoE at the cell surface and mediate ApoE-dependent Aβ endocytosis. A biochemical study by Yajima et al. revealed a stronger binding of ApoE4 isoform to SorLA when compared to ApoE3. On the other hand, ApoE2 isoform exhibited the lowest affinity for SorLA. Similarly, SorLA mediated higher cellular uptake of ApoE3 and ApoE4 in contrast to ApoE2 isoform. The same isoform preference was observed when examining the ApoE-dependent uptake of extracellular Aβ by SorLA [110]. Although these findings suggest a role of SorLA in clearing out extracellular ApoE/Aβ oligomers in an ApoE-isoform-dependent manner, a caution should be taken to its physiological relevance since the experiments were conducted in cell lines that overexpress the receptor. Indeed, Carlo et al. failed to show accumulation of ApoE on endogenous levels in mice genetically deficient for *Sorl1*, thus questioning the significance of this endocytic pathway [93]. SorLA engages in Aβ metabolism also through their direct interaction [111]. Structural studies demonstrated that Aβ can physically interact with a peptide-binding site inside of the propeller tunnel of the VPS10p-domain [109]. Although unable to mediate Aβ uptake when overexpressed in mice, SorLA could target newly generated Aβ peptides from the late endosomal compartment to the lysosomes for its degradation, thereby controlling the amount of Aβ secreted into the extracellular space [109, 111]. Accordingly, a *SORL1* variant p.G511R that segregates with AD in a family [87] was impaired in Aβ binding to the VPS10p-D, providing a mechanistic link how disturbed SorLA functionality may increase Aβ levels [111]. Impairments in these processes may increase neuronal vulnerability, a feature that could escalate the intracellular concentration of Aβ, and thus provide extensive pool of peptides to form cytotoxic AβO.

SorLA is linked to AD also through its interaction with subunits of retromer [177, 195], an evolutionary conserved heteropentameric complex and key player in neuronal protein endosomal recycling [196]. The complex is required for cargo export from the endosome both in the retrograde pathway to Golgi/TGN and recycling to the cell surface [195, 197, 198]. Retromer is composed of two sub-complexes: the trimer VPS26-VPS29-VPS35 forming the core assembly, and the dimer of sorting nexin proteins (i.e. SNX-1 and SNX-2) binding to phosphatidylinositol phosphate membrane lipids [195]. The interaction between SorLA and the retromer occurs via binding of the VPS26 subunit to a hexameric FANSHY amino acid sequence located in the cytoplasmic tail of SorLA. The deletion of the retromer binding site in SorLA is correlated with defective endosomal sorting and the consequent misguidance of cargo proteins. These findings are in agreement with SorLA and retromer forming a functional unit that engage in neuronal endosomal recycling [177]. The tail of SorLA also interacts with SNX-27, another trafficking component that mediates recycling of specific receptors (e.g. AMPAR or NMDAR) from the endosomes to the cell surface, which is often retromer-dependent [176]. Similarly to retromer, SNX-27 downregulation has been linked to AD via increased intracellular production of Aβ [199]. These processes are depicted in the (Fig. 3).

#### SorLA in AD-related neurotrophin signaling and synaptic transmission

Rohe and colleagues showed that BDNF is a specific enhancer of *Sorl1* transcription in vitro and in vivo*,* whereas Sortilin expression was not altered [137]. Accordingly, BDNF treatment was able to reduce Aβ production in the brain of wild-type mice but not of SorLA-deficient animals. These data were supported by neuronal differentiation experiments using human induced pluripotent stem (iPS) cells from AD patients carrying SNPs in *SORL1* [200]. The authors screened a number of *SORL1* genetic risk variants for their ability to respond to BDNF and to produce Aβ. Individuals carrying mutations in the 5’ end of *SORL1* gene showed reduced response to BDNF. Together, the studies demonstrate that SorLA is required for BDNF to decrease the production of Aβ peptides [137, 200]. These findings may have therapeutic potential as the induction of SorLA via BDNF treatment reduces the production of Aβ by 40% (Fig. 4, BOX A) [137]. Later studies uncovered that the BDNF receptor TrkB is not only upstream but also downstream of SorLA. Hence, SorLA can physically associate with TrkB to enhance its anterograde and retrograde trafficking between the cell body and its synaptic destinations, thereby potentiating BDNF-dependent neurotrophic signaling and synaptic plasticity (Fig. 4, BOX C) [143]. Any impairments in this machinery via abnormal SorLA activity therefore likely reduces neurotrophic signaling, synaptic plasticity and ultimately deterioration of synapses, and thus propel the neurodegenerative processes.

More recently, a new role for SorLA in signal transduction recently surfaced thanks to its interaction with Ephrin type-A receptor 4 (EphA4) [96], a tyrosine kinase regulating synaptic structure and functionality [201]. EphA4 binds multiple ligands at the plasma membrane, such as Ephrin A1, which is necessary for EphA4 clustering and its subsequent activation prior to axonal outgrowth and synaptic plasticity [202]. EphA4 exhibits altered distribution in hippocampus of AD patients, where it localizes in Aβ plaques [203]. Aβ oligomers bind EphA4 leading to aberrant activation of the receptor, which ultimately enhances synaptotoxicity and memory deficits in AD mouse models. This interaction is inhibited by SorLA [204–206]. While SorLA does not modulate EphA4 localization, it reduces the aberrant clustering and activation of EphA4/c-Abl pathway triggered by intracellular AβO, particularly in response to Ephrin A1 ligand binding. This is why EphA4 may become a novel therapeutic target for AD [204]. SorLA interacts with the extracellular region of EphA4 via its YWTD/EGF-like domain, by which it controls growth cone collapse in hippocampal neurons [96]. Interestingly, a *SORL1* genetic variant translating into SorLA-T947M receptor mutant was identified in LOAD patients, and carries an amino acid substitution in the YWTD domain [207]. Functional studies exploring this mutation further demonstrated that SorLA-T947M is unable to bind EphA4 (without or in presence of Ephrin A1), and to repress its Aβ-dependent activation. Strikingly, elevated EphA4 activation in human AD brains correlates with the reduced SorLA-EphA4 association [96]. Huang et al. also showed that SorLA-mediated inhibition of EphA4 improved spatial learning and memory of mice injected with human AβO. This mechanism might thus be fundamental for the potential use of SorLA as a neuroprotective agent against cognitive impairment in AD patients resulting from abnormal activation of EphA4. SorLA interaction with EphA4 signaling is schematized in (Fig. 4, BOX B).

Overall, the versatility of SorLA as a crucial player in distinct sorting pathways, amyloidogenic, neurotrophic as well as neuroprotective processes makes the receptor a powerful clinical target for approaching synaptic plasticity impairments, and impeding the AD onset and progressive neurodegeneration.

#### SorLA in AD-related pathology and associated disorders

AD etiology is influenced by non-genomic risk factors such hyperglycemia and insulin resistance, dyslipidemia, and obesity, which may explain why AD is commonly comorbid with diabetes mellitus, cardiovascular diseases, and metabolic syndrome [3]. In the past decade, elevated levels of circulating sSorLA have been correlated to increased body mass index and adiposity [149, 208], and to increased intima-media thickness of carotid arteries in patients with organic coronary stenosis and Type 2 diabetes mellitus [3, 209, 210]. Indeed, overexpression of SorLA in vascular smooth muscle cells enhances their migration from media to intima layer, a key step for formation of atherosclerosis. Hyperglycemic condition was proposed as a promoting factor for SorLA expression in the intimal cells that could contribute to stenosis [209].

Expression of lipoprotein receptors is commonly regulated by lipids, for example essential fatty acids and cholesterol. Accordingly, *Sorl1* expression can be induced by Omega-3 docosahexaenoic fatty acid in primary cortical neurons [211]. Interestingly, obesity and caloric intake in Type 2 diabetes mellitus patients with morbid obesity and cognitive decline enhance *SORL1* expression in peripheral mononuclear cells of plasma, together with other AD-related genes such as *APP, PSEN2, ADAM9,* and *GSK3β*. Such expression profile is reduced after gastric bypass surgery [212] or diet [212, 213]. However, interesting data came from a rat model of mild cognitive impairment and dementia. These rats were chronically fed with high-fat diet to become metabolically obese but of normal weight. In contrast to the peripheral blood, the expression analysis of hippocampi revealed decreased *Sorl1* and *Sort1* mRNA levels while expression of *APP*, *TNFα* and *CASP3* remained increased [214]. Strikingly, patients of normal weight suffering with metabolic syndrome also exhibit decreased SorLA levels in peripheral blood mononuclear cells [215]. Such findings indicate different regulatory mechanisms of SorLA induction in the brain compared to the peripheral tissue. This should be taken in consideration when targeting SorLA in AD patients since high levels of sSorLA might contribute to the development of non-genomic AD risk factors.

The role of SorLA in regulating energy metabolism was also supported by functional experiments. Whittle et al. showed that enhanced sSorLA suppresses thermogenesis in adipose tissue by inhibiting BMP/TGFβ signaling [149]. Strikingly, *Sorl1-/-* mice are protected from high-fat diet obesity due to increased browning of white adipose tissue (WAT), and hypermetabolism [149, 208]. Schmidt et al. further proposed the existence of gene-dosage effect of SorLA on obesity and glucose intolerance as SorLA overexpression in WAT blocked hydrolysis of triacylglycerides and caused excessive adiposity [208]. SorLA expression in WAT was also positively correlated with insulin-induced suppression of lipolysis, and inversely with lipolytic enzyme activities. They suggested that SorLA expressed by adipocytes in WAT functions as a sorting and recycling factor for insulin receptor (IR) that redirects IR molecules from endosomes to the plasma membrane, to enhance IR surface expression, and to reinforce insulin signaling [208]. Interestingly, high expression of SorLA was also found in adipocyte precursors in juvenile visceral WAT. The authors proposed that upon high-fat diet, elevated levels of SorLA in these cells increases their sensitivity to insulin stimulation, which promotes the mitotic expansion of the visceral precursor cell pool in juvenile mice. In contrast, low levels of SorLA in subcutaneous precursors blunt their response to insulin, and prevent their proliferation [216]. Taken together, these studies highlight the complex regulation of SorLA expression and its functional versatility, underscoring SorLA as a key regulator also of activities that might not be directly involved in canonical AD pathophysiology, but could further contribute to it.

### Sortilin biology and its role in AD

Belenguez et al. recently identified *SORT1* as a high-impact AD risk gene [14]. The magnitude of the association was similar whether the patients were diagnosed by questionnaires or clinical evaluation, which substantiates the robustness. Among the SNPs, the lead observations encoded a rare missense variant that substitutes an arginine with a glutamic acid at residue 302, which is located in the β-propeller of the VPS10p-domain, and harbors the ligand binding site. In a Swedish cohort, Anderson et al. identified *SORT1* SNPs that are associated with reduced disease risk, suggesting the existence of gain of function variants [217]. On contrary to SorLA, Sortilin protein levels are higher in temporal cortex and cerebellum of some AD patients, and in a transgenic mouse model of AD (APP/PS1dE9) [103, 104, 113, 218]. It has been suggested that this increase positively correlates with the severity of AD pathophysiology since no changes are observed in patients with mild cognitive impairments [219]. One explanation for this apparent paradox could be that increased Sortilin may act as a compensatory event to counteract disease progression in those AD patients not harboring a risk *SORT1* SNP. Interestingly, C-terminal fragments of Sortilin are deposited in neuritic Aβ plaques in human cerebrum [218] but not in brains from transgenic AD mouse models nor aged macaques exhibiting amyloid plaque deposition [220] suggesting the interspecies differences in the formation/composition of senile plaques in regards to VPS10p-D receptors. Despite an overlap in the sorting machinery used by Sortilin and SorLA, their structural difference and opposite regulation of expression suggest that they exhibit distinct activities in AD pathogenesis.

Sortilin was identified as the second member of the VPS10p-D receptor family in 1997 [221]. Its expression is highly abundant in the CNS and in peripheral nervous system (PNS) neurons [142, 222], and it is enriched in forebrain, particularly in temporal cortex [222, 223]. Sortilin binds a vast number of ligands to control their sorting or signaling activities [153]. Important roles of Sortilin is to mediate anterograde trafficking from the secretory pathway along neurites and to endosomes and lysosomes, and to mediate endocytosis and retrograde transport from the cell surface to the TGN by evading lysosomal targeting and degradation [224]. The low pH within the endolysosomal compartments causes conformational change and dimerization of Sortilin. This results in collapse of the binding site, which triggers the release of Sortilin’s cargo, and enables recycling of the receptor back to the cell surface by TGN-coupled retrograde transport [155]. Similarly to SorLA, Sortilin trafficking pattern is controlled by binding to a number of cytoplasmatic proteins including GGA1-3 [224], AP1 and -2 [225], Rac-p21-activated kinases 1 to 3 (PAK1-3) [226], and Ras-related protein (Rab7b) [227]. The retromer complex also binds Sortilin and is required for its proper sorting [228, 229]. Interestingly, Belenguez et al. also identified the retromer subunit *SNX-1* as a novel top-risk gene for AD, similarly to *SORT1* [14].

Among other ligands, Sortilin transports BACE1 and APP, by which it regulates the production and endocytosis of sAPP [97]. Similarly, Sortilin facilitates the uptake of AβO [112] and ApoE [93]. Perhaps most well established is the role of the receptor in regulating neurotrophic signaling. It forms a receptor complex with p75^NTR^ at the plasma membrane by which it modulates binding of proNTs, and controls their pro-apoptotic activity [52, 53, 134, 140]. Sortilin also enables the anterograde transport of neurotrophin receptors such as TrkB and the secretion of its ligand BDNF [138, 142], the latter being dependent on the complex formation with Huntingtin associated protein-1 (HAP1) [230]. Sortilin undergoes ectodomain shedding at the cell surface as well as during intracellular trafficking which inhibits lysosomal degradation of its cargos including BDNF [117]. Put together, any impairment of Sortilin function affects cell survival and homeostasis in the brain, particularly during aging [53]. In the following paragraphs, we will discuss studies that highlight the function of Sortilin in AD and related disorders.

#### Sortilin interactions in amyloidogenic cascade

**Fig. 5:**
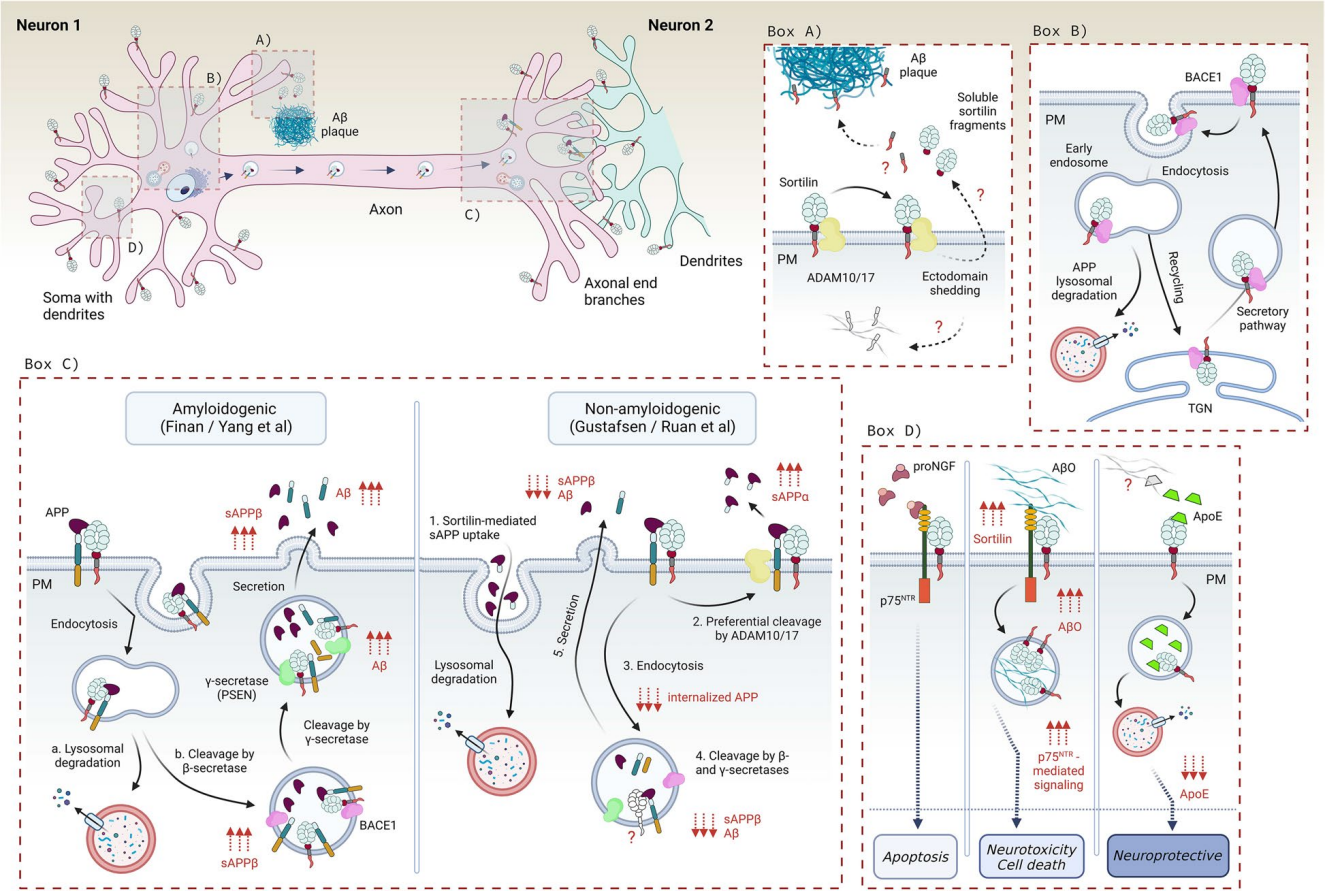
Functional involvement of Sortilin in AD-related signaling. Sortilin localizes in sorting vesicles and on the plasma membrane (PM) in neuronal somas, dendrites and axons. Box A) Sortilin undergoes ectodomain shedding by ADAM10/17, which produces soluble Sortilin fragments. In humans, C-terminal fragments are found within the Aβ plaques, however, their precise origin and trafficking route is unknown (marked as “?”). Box B) Sortilin binds BACE1 in TGN and facilitates its intracellular trafficking via anterograde and retrograde pathways, the later directed either towards the recycling pathway or for the lysosomal degradation. Box C) Sortilin binds APP at PM; however, its involvement in APP processing is controversial. *Left panel*—Sortilin binds APP at axonal PM where they undergo internalization. Sortilin either traffics APP for its lysosomal degradation (*a.*) or engages in amyloidogenic pathway by enhancing APP cleavage by BACE1 (*b.*), subsequently causing an increased formation and secretion of sAPPβ and Aβ. Sortilin is also a PSEN1/2 substrate. *Right panel* – Sortilin has a neuroprotective role as it mediates the uptake of soluble APP from the extracellular space (*1.*) for lysosomal degradation thus decreasing their extracellular concentration. Moreover, Sortilin binds APP in neurites where it drives its preferential cleavage by ADAM10/17 (*2.*), thus elevating sAPPα levels. Consequently, there is less APP internalized (*3.*) prior the sequential cleavage by β- and γ-secretases (*4.*), resulting in decreased production of sAPPβ and Aβ (*4.-5.*). However, the molecular mechanisms are rather unknown (marked with “?”). Box D) Upon proNGF binding, Sortilin forms a complex with p75^NTR^ receptor, which mediates pro-apoptotic cell responses (*left*). The presence of AβO increases Sortilin expression, which likely enhances the formation and activity of Sortilin-p75^NTR^ complexes. Sortilin-p75.^NTR^ complex binds and internalizes AβO leading to increased intracellular neurotoxicity, and later cell death (*middle*). Sortilin can also bind and sequester extracellular ApoE, subsequently facilitating its lysosomal degradation, which has a neuroprotective effect (*right*). It is not clear if Sortilin sequesters ApoE-Aβ complexes (marked as “?”)

Sortilin transports BACE1 and APP [97, 103, 105], and regulates the production and endocytosis of sAPP [97]. The intracellular trafficking of BACE1 between TGN and endosomes is necessary for its functioning [231, 232], and it is governed by adaptor proteins including GGA3 [233], which targets BACE1 for its lysosomal degradation [234]. Accordingly, inhibition of GGA3 activity results in local, cytotoxic accumulation of BACE1 in axonal swellings leading to enhanced BACE1 activity, and later axonal dystrophy observed even before enhanced levels of Aβ [235]. Indeed, reduced levels of GGA3 protein in AD brains correlate with increased levels of BACE1, APP, and Aβ [234, 236]. Sortilin cytoplasmic tail also contains a consensus motif for binding GGA adaptor proteins that facilitates Sortilin transport from the Golgi compartment to endosomes and lysosomes [224, 237, 238]. Finan et al. showed that Sortilin forms a complex with BACE1 in the human brain, by which it regulates retrograde trafficking of BACE1 from the early endosomes back to the perinuclear region of TGN (Fig. 5, BOX B). The authors further s

uggested that Sortlin-BACE1 interaction facilitates BACE1-mediated first cleavage of APP, leading to an increased formation of sAPPβ and accumulation of Aβ peptides. Importantly, they showed that this process is partially regulated by Sortilin’s but not by SorLA’s or SorCS1b’s cytoplasmic tails [103]. These data highlight the non-redundant, pro-amyloidogenic function of Sortilin, and the specificity of its cytoplasmic tail in BACE1-dependent first cleavage of APP (Fig. 5, BOX C).

Later studies revealed that Sortilin’s extracellular domain can bind APP in vitro and in vivo [97, 105], and that Sortilin is a substrate for all three secretases involved in APP processing [91, 115, 117, 118, 239, 240]. The α-secretases ADAM10 [117, 118] and ADAM17 [115] that are responsible for the first non-amyloidogenic cleavage of APP, also facilitate Sortilin ectodomain shedding [117]. Interestingly, Sortilin is a target of γ-secretase [91, 239], and it has been a predicted substrate for BACE1-mediated processing [240]. These data suggest that Sortilin might be cleaved in the same subcellular compartments as APP, perhaps when bound to each other. Indeed, Yang et al. showed that Sortilin co-localizes with APP in perinuclear space and in axons of cultured neurons, where it facilitates APP trafficking from the late endosomes to the lysosomes for its degradation [105]. Remarkably, C-terminal fragments of Sortilin liberated by γ-secretase are deposited in neuritic Aβ plaques of human cerebrum [218]. Even though there is no experimental explanation yet, one might speculate that Sortilin can be sequentially processed when in complex with APP resulting in shedding of Sortilin’s ectodomain by the α- of β-secretase, later followed by γ-secretase cleavage. The C-terminal tail may be then either released from neurons, and subsequently captured in Aβ plaques, or it may associate with Aβ already intracellularly, and be released upon neuronal demise. Both possible interpretations are schematized in (Fig. 5, BOX A).

The cleavage of APP and ectodomain shedding of Sortilin may explain why the C-terminal domain of the receptor accumulates, and represent a prominent constituent of the amyloid plaques [218, 220]. However, these data were contradictory with Gustafsen et al. who proposed that APP and Sortilin primarily co-localize in the neurites [97]. Gustafsen et al. found that Sortilin directly enhances the production of secreted sAPPα, and mediates uptake of the extracellular sAPP. Interestingly, the authors detected decreased levels of sAPPβ in the presence of Sortilin, contrary to the Finan study [103]. The authors proposed that Sortilin increases APP processing in non-amyloidogenic pathway (sAPPα) when compared to the pro-amyloidogenic pathway (sAPPβ). This is in contrast to SorLA that reduces the levels of both, sAPPα and sAPPβ [97]. These observations are supported by a recent study by Ruan et al. that used a triple transgenic AD model (APP/PSEN1) deficient in Sortilin. They reported that the lack of Sortilin enhances the Aβ deposition, neuronal loss, and astrocytic activation during aging. They also demonstrated that Sortilin’s intracellular domain mediates APP degradation [104]. According to these studies, Sortilin thus has a neuroprotective feature against APP-dependent amyloidosis likely because it consequently decreases the cleavage of cytotoxic sAPPβ. These two opposing models are depicted in (Fig. 5, BOX C). Nevertheless when combined, these studies demonstrate that the proteolytic cleavage of APP differs dependent on the expression levels, protein localization, and perhaps activity of the specific VPS10p-D receptors. For example, high Sortilin levels might increase APP cleavage and Aβ release, while increased SorLA retains APP in TGN, thus protecting the cell against cytotoxicity. However, the biochemical studies addressing Sortilin localization and the mechanism of its action on APP processing are not consistent, likely due to the differences between the experimental models. For instance, the Finan and Yang studies employed a C-terminally tagged Sortilin variant [103, 105], whereas Gustafsen et al. used the untagged receptor [97]. The C-terminal tagging will likely lead to aberrant Sortilin localization since binding of the GGA adaptors requires the C-terminal acid cluster of the receptor tail, as demonstrated by Cramer et al. [241]. More functional studies using either untagged proteins or both models in parallel are required to determine the precise molecular mechanism by which Sortilin regulates APP transport and catabolism in non/amyloidogenic pathways.

It is well established that Sortilin forms a receptor complex with p75^NTR^ which mediates pro-apoptotic cell responses [52, 134]. Accumulation of extracellular Aβ oligomers facilitates the neurotoxicity and neuronal cell death via their physical binding to p75^NTR^ [242], while the addition of Aβ peptides increases expression of Sortilin in vitro likely via activation of the p75^NTR^/RhoA pathway [113]. In line with these data, Takamura et al. found that extracellular AβO, as opposed to non-oligomerized Aβ, act as Sortilin ligands, and that Sortilin loss-of-function suppresses AβO-targeted autophagy and AβO-induced cell death [112]. Interestingly, extracellular AβO triggers the co-localization of Sortilin with p75^NTR^ at the neuronal surface, proposing a model where Sortilin-p75^NTR^ receptor complex mediates apoptotic response upon binding of AβO, a mechanism that can contribute to the progressive neurodegeneration in AD patients [112].

Besides regulating the amyloidogenic cleavage of APP, Sortilin is the major neuronal ApoE receptor for endocytic uptake and catabolism of Aβ [93]. Binding studies revealed that Sortilin is able to interact with each of the three major ApoE variants with slightly higher affinity for the cytotoxic ApoE4 [93]. Carlo et al. further showed that crossing AD mice (PDAPP and FAD lines) with mice deficient in Sortilin increases Aβ and ApoE levels in cortex and hippocampus [93]. There was no difference in sAPPα, sAPPβ, and BACE1 expression. Importantly, no changes in ApoE levels were seen in glia cells, known to be the main site of apolipoprotein synthesis. Remarkably, however, the absence of Sortilin significantly attenuated cellular uptake of ApoE-Aβ complexes, demonstrating that impaired ApoE clearence by Sortilin causes accumulation of Aβ in the brain [93]. Given Aβ handling was unchanged in knockout neurons in the absence of ApoE, this suggests that Sortilin exclusively but potently mediates the Aβ uptake when in complex with ApoE. These observations were in marked contrast to SorLA deficient mice that exhibited no accumulation of ApoE, supporting the model of Sortilin’s direct effect on ApoE and Aβ turnover [93]. Most recently, the same research group studied the relevance of the Sortilin-mediated uptake of ApoE for brain lipid metabolism [243, 244]. They found that Sortilin is required to accumulate and facilitate the metabolism of polyunsaturated fatty acid into endocannabinoids; lipids with potent anti-inflammatory and neuroprotective functions. Remarkably, Sortilin expression had no impact on endocannabinoid production in transgenic mice expressing the AD risk variant ApoE4, demonstrating that this function was restricted to the ApoE3 isoform. The authors explain this apparent paradox by ApoE4 being unable to uncouple from Sortilin in the endosomal compartment, which disrupts recycling and re-exposure of the receptor at the plasma membrane. The combined findings are schematized in (Fig. 5, BOX D) and suggest a protective role of Sortilin in AD by lowering Aβ levels, reducing production of neuroinflammatory cytokines [244, 245], stimulating synapse function, and sustaining neuron viability.

#### Sortilin in AD-related neurotrophin signaling and synaptic transmission

Sortilin is also an important neurotrophic receptor. The proneurotrophins proBDNF, proNGF, and proNT3 can form ternary complexes with Sortilin and p75^NTR^, which promotes signaling towards neuronal cell death [52, 53, 134, 140]. For instance, the proNGF-Sortilin-p75^NTR^ complex is fundamental for pruning of retinal ganglion cells, and is involved in degeneration of acutely injured and senescent neurons [53]. Surprisingly, Sortilin and p75^NTR^ also support trophic activities induced by mature neurotrophins. While it was well-established that p75^NTR^ strengthens binding of NGF, BDNF and NT3 to their cognate receptor [9], Vaegter et al. found that Sortilin works in tandem with p75^NTR^ to empower neurotrophin signaling by facilitating the anterograte transport of their respective Trk receptors along axons to postsynaptic densities [142]. In particular, they found that peripheral neurons in Sortilin deficient mice on the genetic background of p75^NTR–/–^ were normally developed but underwent a marked age-dependent neurodegeneration leading to severe neuronal demise, and sensory and motor impairments [142].

Sortilin also regulates BDNF secretion. proBDNF sorting to lysosomes is blunted upon ADAM10-mediated and activity-dependent Sortilin’s ectodomain shedding from the cell membrane and intracellular compartments, thus providing more BDNF for secretion [117]. Interestingly, proBDNF binding to HAP1 is important for Sortilin to traffic proBDNF in neurites, and to stimulate BDNF processing and secretion [138, 230]. Sortilin may also target proBDNF for lysosomal degradation to blunt BDNF secretion [117]. When and how Sortilin acts as a regulatory switch to increase or decrease BDNF secretion has not yet been resolved, but possibly the levels of the receptor expression are involved. To further increase the complexity, proBDNF increases the expression of Sortilin and p75^NTR^ in vitro*.* This prevents proteolytic cleavage and processing of proBDNF, possibly as a consequence of its binding to the high-affinity Sortilin and p75^NTR^ receptor complex [113, 134]. Chen et al. showed that binding of proBDNF to Sortilin is mediated by the prodomain of proBDNF (amino acids 44–102), and that this interaction is reduced in the BDNF-Val66Met mutated protein [138]. They further showed that Sortilin traffics wild-type BDNF into the pathways for regulated secretion, whereas the BDNF-Val66Met mutation disrupted this sorting [138]. The reduced binding of BDNF-Val66Met to Sortilin may explain the faster cognitive decline in AD that harbors this mutation [37–39]. A recent study from Fleitas et al. further proposed that accumulation of reactive oxidative species (ROS) in AD patients stabilizes proBDNF and disables its maturation into BDNF. This will subsequently increase proapoptotic signaling and blunt trophic actions of BDNF [57]. The authors examined hippocampal tissue from AD patients and found a significant increase in Sortilin and proBDNF levels, which translated into an enhanced proBDNF/BDNF ratio in CSF of the patients. Strikingly, when the authors applied CSF from AD patients on cultured WT hippocampal neurons, they observed enhanced proBDNF-p75^NTR^-dependent apoptosis in contrast to CSF from healthy controls [57]. These data propose the proBDNF/BDNF ratio as a biomarker for AD diagnosis or disease progression.

A hallmark of AD is the early and progressive dysfunction, synaptic loss, and degeneration of basal forebrain cholinergic neurons (BFCN). The reason for their selective vulnerability is not fully understood but BFCN are reliant on mature NGF that is produced by and transported retrogradely back from their target neurons in cortex and hippocampus. In AD patients, NGF levels and TrkA expression are decreased in BFCN whereas proNGF is increased [10, 62, 74, 246]. Given the expression of p75^NTR^ and Sortilin is preserved in AD brains, these alterations favor an increase in proapoptotic signaling on the expensive trophic stimulation. Indeed, transgenic mice expressing an anti-NGF-antibody electively targeting mature NGF and leaving the proform unperturbed, exhibited accelerated BFCN pathology and cognitive impairments [247]. Likewise, mice with proNGF overexpression develop age-dependent memory impairments, cholinergic deficits, and, surprisingly, also increased formation of Aβ oligomers [248]. To demonstrate the requirement of the p75^NTR^-Sortilin receptor complex for executing these functions, BFCN pathology and cognitive impairments were rescued in mice expressing the neutralizing anti-NGF antibodies on the genetic background of *p75*^*NTR–/–*^ and *Sort1*^*–/–*^, respectively [249, 250].

#### Sortilin in AD-related pathology and associated disorders

Increased Tau phosphorylation, its subsequent misfolding and prion-like spreading are common pathological features in AD brains [4]. By using mutant Tau transgenic mice (P301S), prion-propagation assay, and inhibitory antibodies against Sortilin, Johnson et al. found that Sortilin activity suppresses replication of Tau prion in the forebrain thus protecting it against neurotoxic pTau aggregation. On contrary, Sortilin expression is lower in the hindbrain where it does not protect against p-Tau accumulation [223]. AD shares several other mechanisms with Prion diseases, a group of fatal neurodegenerative disorders which major genetic component is neuronal Prion protein (PrP^C^). Prp^C^ is a transmembrane receptor localized in lipid rafts [251] that regulates neuronal excitability and neurite outgrowth [252]. PrP^c^ inhibits BACE1 and Tau expression, which subsequently reduces the levels of Aβ in the brain [253]. During AD, PrP^C^ converts into its polymerizing, misfolded form called scrapie isoform PrP^Sc^, which binds AβO, and transduces their cytotoxic signals across the neuronal membrane [254–256] causing synaptic failure and cognitive impairments [257–261]. Upon AβO binding, PrP^C^ is phosphorylated by Fyn kinase leading to hyperactivation of NMDAR channels, and subsequent glutamate toxicity. Furthermore, AβO-PrP^C^ complex physically binds its co-receptor the metabotropic glutamate receptor 5 (mGluR5), which activates the Fyn kinase, followed by eEF2 phosphorylation, and consequent loss of neuritic spines and memory [262–264]. At the plasma membrane, PrP^C^ binds Aβ oligomers with high-affinity, yet during aging, AβO-PrP complexes eventually accumulate extracellularly in form of plaques, even before AD manifestation [265]. Thus, targeting receptors involved in AβO signal transduction such as PrP^C^ and mGluR5, or disrupting the AβO-PrP^C^ complex holds therapeutic potential in AD patients [266]. Indeed, a recent study discovered that a PrP^C^ antagonist blocks the protein aggregation, and rescues the Aβ-related synapse loss and memory deficits in AD transgenic mice [267], similarly to mGluR5 antagonist [262]. Strikingly, Uchiyama et al. showed that Sortilin is neuroprotective against the prion spreading as it internalizes PrP^C^ and PrP^Sc^, and transports them into lysosomes for their degradation. However, PrP can be a determinant of Sortilin activity since increased accumulation of cytotoxic PrP^Sc^ leads to lysosomal degradation of Sortilin resulting in progressive propagation of PrP^Sc^ [268]. Accordingly, Sortilin deficiency leads to early accumulation of PrP^Sc^, and accelerated disease progression and death of the mice. These observations pinpoint the neuroprotective role of Sortilin sorting against protein misfolding and prion-related spreading that might include internalization of other proteins than just Tau and PrP.

Along with aggregation of TAR DNA-binding protein 43 (TDP-43), Tau pathology is also a hallmark of frontotemporal dementia (FTD) [269]. Haploinsufficiency for *GRN*, a gene encoding progranulin (PGRN) that is a protein with widespread neuroprotective and anti-inflammatory functions, is one possible causative for FTD [270]. Haploinsufficent patients have a 50% reduction in PGRN levels, why inhibiting its clearance from the brain extracellular space has been proposed as a therapeutic approach. In a human cohort, a SNP in *SORT1* that increases Sortilin expression is associated with reduced plasma PGRN concentration [271]. Hu et al. found that Sortilin binds PGRN and mediates its endocytic uptake and extracellular clearance, and that preventing its function can normalize PGRN levels in *Grn*^+*/–*^ mice [272]. Accordingly, a phase II clinical trial using a Sortilin inhibiting antibody recently achieved positive results in FTD patients [270].

TDP-43 pathology is also common in AD with more than 55% of the patients having these inclusions [273]. Interestingly, *SORT1* can be alternatively spliced to generate an mRNA transcript named Ex17b that includes a premature stop codon translating into a truncated soluble receptor variant that retains its ligand binding abilities [274, 275]. In the healthy brain, nuclear TDP-43 inhibits this splicing which leads to exclusion of Ex17b, and expression of the full-length receptor. In FTD and AD, the nucleus is depleted from TDP-43, favoring its cytoplasmic aggregation. This will drive splicing and produce the soluble and dominant-negative Ex17b decoy receptor [274, 275]. The functional link between Sortilin, FTD, and AD is further supported by Bellenguez et al. who, in addition to *SORT1*, also identified *GRN* as a critical risk gene in AD [14]. Remarkably, the rare Sortilin K302E variant, which is a predicted loss of function mutation present in AD patients [14], has been also identified as a causal patient-only variant in FTD patients [94].

Sortilin has been also extensively studied for its contribution to hyperlipidemia, cardiovascular disease, diabetes mellitus, obesity, and metabolic syndrome [275, 276]. In the following paragraphs we will pin point the key observations on Sortilin’s involvement in glucose and lipid metabolism, while we refer to these two recent reviews [276, 277] for complete overview of the topic.

Obesity in humans and mice is associated with downregulation of Sortilin in subcutaneous WAT and liver [278–280], while Sortilin deficiency results in slower weight gain on western diet [281, 282]. These findings are in contrast to SorLa which expression is upregulated upon hyperglycemic condition, obesity, and high caloric intake [209, 212, 213]. Interestingly, *Sort1*^*–/–*^ mice on the genetic background of the LDL receptor (LDL-R) knockout exhibit improved function of brown adipose tissue [283]. These data point at a negative feedback-loop where high body mass index downregulates Sortilin expression to prevent further weight gain. Other studies reported that deprivation of glucose attenuates Sortilin levels in skeletal muscle [284], and that insulin resistance induces hepatic degradation of Sortilin in mice [285], suggesting Sortilin’s role also in glucose homeostasis. Indeed, Sortilin enables the biogenesis of glucose transporter 4 (GLUT4) storage vesicles by binding and targeting GLUT4 into maturing vesicles, and by controlling GLUT4 recycling [286–290]. When insulin levels decline, GLUT4-positive vesicles are retrieved from the plasma membrane to the endosomal compartment. Here, the cytoplasmic tail of Sortilin will engage the retromer complex, enabling GLUT4 retrograde transport and reuse [291]. Paradoxically, studies on *Sort1*^*−/−*^ mice have so far not shown any signs of insulin resistance or reduced glucose handling [282, 292]. Only one study found increased glucose uptake and insulin sensitivity [282].

A vast number of GWAS have identified SNPs in *SORT1* strongly associated with LDL cholesterol and risk of cardiovascular disease [277]. Kathiresan et al. [293]and Musunuru et al. [294] were first to identify a SNP that associated with LDL cholesterol. Carriers of the minor allele have lower LDL and increased hepatic expression of Sortilin, suggesting that Sortilin is protective against cardiovascular disease. Unfortunately, studies in mice have yielded conflicting results about the directionality of LDL cholesterol versus levels of Sortilin expression. Using viral-mediated overexpression, Musunuru et al., confirmed an inverted correlation between Sortilin expression plasma LDL [294]. A similar directional correlation was confirmed by Rader’s group who suggested that Sortilin destines LDL for lysosomal degradation during its biosynthesis, and enables hepatic LDL clearance [280, 295, 296]. In marked contrast, Kjølby et al. showed the opposite correlation; i.e. low Sortilin expression equals low LDL cholesterol [297]. The authors demonstrated that Sortilin facilitates the formation and hepatic export of ApoB10-containing lipoproteins. Another study reported that Sortilin also supports secretion of Proprotein convertase subtilisin/kexin type 9 (PCSK9), which targets the LDL receptor for degradation, thereby further increasing plasma cholesterol [298]. The confusion for the discrepant results remains, with a similar number of papers arguing for a positive and negative correlation between Sortilin expression and plasma lipids, respectively. The conflicting results from animal models have been largely discussed [276, 277], and might be explained by the strong interplay between glucose and lipid metabolism, which is highly regulated by the composition of diet, weight, age, genetic background, and hormonal stimulation. Moreover, Sortilin expression itself is greatly regulated by metabolic activity and, as described in the previous sections, even minor changes in receptor expression may have substantial functional implications, similarly to SorLA.

To conclude, there is substantial evidence that Sortilin regulates a number of activities involved in Aβ production and clearance, neurotrophic signaling, tau pathology, prion-related spreading, as well as metabolic disorders that are comorbid with AD. The complex modalities by which Sortilin operates with some functions being protective and others detrimental, may explain why certain *SORT1* SNPs reduce whereas others increase the AD risk.

### SorCS1 biology and its role in AD

**Fig. 6:**
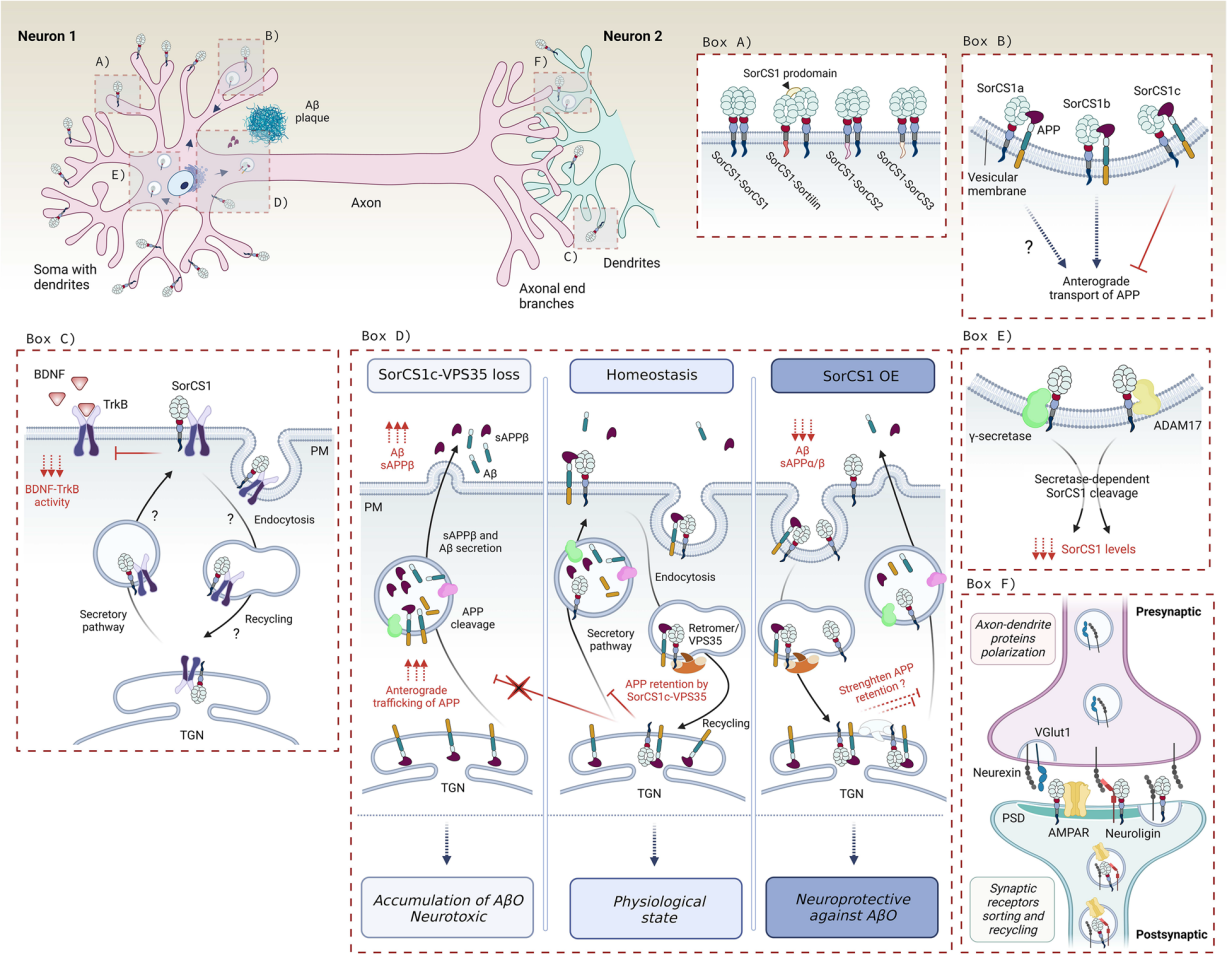
SorCS1 localization and signaling relevant to AD. SorCS1 localization is restricted to cell soma and dendrites. Box A) SorCS1 forms homodimers, but also heterodimers with Sortilin via SorCS1 prodomain, and with SorCS2/3. Box B) SorCS1 binds APP in vesicular compartments; however, SorCS1 variants control APP sorting in different manner. While SorCS1b mediates APP trafficking towards PM, SorCS1c blocks it. Box C) SorCS1 binds TrkB, which inhibits TrkB activation by BDNF stimulation. SorCS1 might be responsible (marked “?”) for TrkB sorting between TGN, PM and recycling pathway. Box D) This figure schematizes the possible consequences of SorCS1 loss and gain of function. In homeostatic state (*middle panel*), SorCS1 binds APP and the retromer complex via its VPS35 subunit. This protein complex is internalized and later recycled into TGN. SorCS1c remains in complex with APP and retromer, which retains APP in TGN, and subsequently regulates its cleavage by BACE1 and γ-secretase. This way SorCS1 could control the physiological levels of secreted sAPPβ and Aβ. The abortion of SorCS1c-VPS35 interaction (*left panel*) enhances the APP anterograde trafficking causing an increased production and release of neurotoxic Aβ and sAPPβ. SorCS1 overexpression (OE; *right panel*) seems to strengthen the APP retention in TGN, thus significantly reducing the production and secretion of Aβ and sAPPβ, which has a neuroprotective effect against the formation of Aβ oligomers. Box E) SorCS1 is a substrate for PSEN1/2 and ADAM17, which attenuates its protein levels. However, molecular mechanisms involved in this regulation are unknown. Box F) SorCS1 sorts and recycles a number of synaptic receptors including Neurexin, AMPAR or Neuroligin at the postsynaptic side, by which it establishes the correct axon-to-dendrite polarization of synaptic proteins, processes critical for correct neurotransmission, connectivity, and synaptic plasticity

SorCS1 was identified as the first SorCS protein from mouse brain by Hermey et al. in 1999 [129], followed by SorCS2 and SorCS3 in 2001 [123, 131]. SorCS1, 2 and 3 hold high structural homology, and thus they likely partially overlap in their functions when expressed in the same tissue. They mostly differ from each other in their cytoplasmic tail, which interacts with various adaptors to control cellular trafficking and signaling [121, 131]. SorCS1 is unique as it exists in (at least) five isoforms, SorCS1a-e, that vary in their cytoplasmic tails and in their expression pattern. When overexpressed, murine SorCS1a undergoes rapid internalization via its binding to clathrin adaptor AP-2, whereas SorCS1b predominates at the plasma membrane, and shows little trafficking activity [299]. Rather, SorCS1b may engage in signal transduction given that its cytoplasmic tail contains consensus sequences for a SRC Homology 3 Domain (SH3) binding motif. SH3 motif is recognized by protein tyrosine kinases including Src family such as Src, Fyn, Blk or Lyn, which regulate many cellular functions including cell proliferation, differentiation, migration, and survival [300]. SorCS1c can bind VPS35, the core protein of the retromer complex that controls transport out of the endosomal compartment but its intracellular domain also harbors interaction site for adaptors involved in cellular signaling [106, 133, 148, 299]. As a consequence, SorCS1 is present both in the soma, dendritic vesicles, and at the plasma membrane in neurons [107, 301, 302] (Fig. 6). The physiological functions of the receptor variants are only slowly emerging and needs to be investigated in more detail. SorCS1 can form homodimers as well as heterodimers with SorCS2 and -3 but the functional consequence has not been studied [303]. However, the N-terminal propeptide of human SorCS1 can bind Sortilin, which substantially reduces the ability of Sortilin to mediate cellular uptake of its ligands and hampers its ability to support signaling by ciliary neurotrophic factor [304] (Fig. 6, BOX A). SorCS1 can also bind SorLA and VPS35, while *Sorcs1*^*−/−*^ mice exhibit decreased expression of these two genes in the brain [92]. SorCS1 shows the highest expression in neurons from cerebral cortex, amygdala, hippocampus, and thalamus, while it is mostly expressed in forebrain during mouse development [122, 301]. SorCS1 expression is very dynamic and can be regulated by synaptic activity [121, 301]. For example, kainic acid, a glutamate analog, induces high expression of SorCS1 in the hippocampus [121, 301]. More physiologically, SorCS1 has been detected as the most correlated gene expressed in mouse hippocampus after novel object recognition performance [305]. Rao-Ruiz et al. further showed that SorCS1 is upregulated 220-fold in engram cells, the cells that encode a specific memory during memory consolidation [126]. Whether this upregulation is specific to one or more of the splice variants is not known.

SorCS1 involvement in learning and memory is further supported by several human genetic studies that linked SNPs in *SORCS1* gene to memory retention [306] and the risk of AD [83, 84, 108, 307–312]. Accordingly, gene expression analysis of amygdala from 19 AD patients revealed significantly lower *SORCS1* expression compared to healthy controls [108]. Furthermore, *SORCS1* genetically interacts with SNPs in *APOE* [313, 314], *SORCS2* and *SORCS3* [84], respectively, to increase AD risk suggesting that these proteins functionally associate in shared actions. Several SNPs in *SORCS1* have also been linked to neurodegenerative transmissible spongiform encephalopathies caused by accumulation of PrP^sc^. Given that prion seeding characterizes many deteriorating brain disorders, it may suggest a broader involvement of SorCS1 in neurodegenerative processes, similarly to Sortilin [315].

#### SorCS1 interactions in amyloidogenic cascade

There are several functional studies uncovering the importance of SorCS1 in Aβ metabolism. They demonstrated that APP can physically interact with both the SorCS1a, -b, and -c isoforms suggesting that SorCS1 may function in trafficking of and potentially also signaling by APP [106–108, 133]. In accordance with a function in cellular sorting, SorCS1c but not SorCS1b retains APP from insertion into anterogradely transported vesicles in hippocampal neurons [101, 107] (Fig. 6, BOX B). Knockdown of SorCS1a and -c expression in neuroblastoma cells increase Aβ production [107, 108]. Furthermore, the disruption of SorCS1c internalization motif YAQM in its cytoplasmic tail perturbed APP sorting through endosomal compartments, decreased retrograde TGN trafficking, and increased Aβ production, thus resembling SorCS1 loss of function [106]. On the other hand, overexpression of SorCS1a, -b, and -c decreased levels of Aβ, as well as lowered levels of secreted APP products [92, 106, 108] (Fig. 6, BOX D). Studies in SorCS1 knockout mice have confirmed many of the in vitro observations. Hence, receptor deficiency translated into an increase in APP C-terminal fragments in brain of females but apparently the male mice were not affected by SorCS1 deletion [106]. This is especially interesting given that the genetic association between SorCS1 and AD was strongest for women. Strikingly, Hermey et al. recently found that the progressive amyloid plaque formation in aged AD mice (APP/PS1) decreases the levels of SorLA, SorCS1, and SorCS3 in frontal cerebral cortex, and to a minor extent also in hippocampus, forming a virtuous self-amplifying loop [101]. It has also been reported that SorCS1 itself is a substrate for PSEN-dependent γ-secretase cleavage [91] and ADAM17 α-secretase, which will downregulate the receptor expression, adding yet another loop to the complex function of SorCS1 (Fig. 6, BOX E) [115].

#### SorCS1 in AD-related pathology and associated disorders

Type 2 diabetes mellitus (T2DM) is characterized by hyperglycemia and insulin resistance. T2DM is commonly comorbid with AD, increasing the risk of AD diagnosis by approximately 2–3 folds [316–318]. On contrary, approximately 80% of AD patients exhibit insulin resistance and impaired glucose handling [319–321], suggesting that, likely, there are common molecular pathways involved in both disorders. Hyperinsulinemia is associated with impaired cognitive performance, while hyperglycemia increases Aβ accumulation, exacerbate oxidative stress, neuroinflammation, and mitochondrial dysfunction, ultimately leading to impaired neuronal integrity and neurodegeneration. In recent years, this shared comorbidity is sometimes called Type 3 diabetes mellitus [321–325]. Interestingly, SorCS1 regulates insulin metabolism and secretion as it facilitates the release of insulin from pancreatic β cells [326], in contrast to SorLA which establishes cellular sensitivity to insulin by trafficking the insulin receptor to the plasma membrane [208]. Therefore, it is not surprising that impairments in SorCS1 activity are strongly associated with Type 1 and Type 2 diabetes [327–331]. Strikingly, T2DM has been identified as a contributing risk factor to AD etiology patients carrying SNPs in SorCS1 [332]. Put together, these data suggest that aberrant SorCS1 function in the brain might fail to not only integrate the APP sorting and processing, but also the signaling of another contributing trophic factor, insulin. Impairments in SorCS1 can thus lead to the pathophysiological events that are behind the AD onset and progressive neurodegeneration.

#### SorCS1 in AD-related neurotrophin signaling and synaptic transmission

A function of SorCS1 in neurotrophin signaling has been only sparsely studied. When exploring SorCS1 interactome using recombinant SorCS1-ecto-His protein as a bait in synaptosomes from mouse brain, Savas et al. identified TrkB as a candidate binding partner of SorCS1 [133]. In marked contrast to mice lacking SorLA [143] or Sortilin [104, 142], Subkhangulova et al. further showed increased TrkB signaling in a knockout mouse line that is devoid in both *Sorcs1* and *Sorcs3* expression [144]. The authors proposed that SorCS1 binds TrkB and likely facilitates its intracellular trafficking and signaling abilities (Fig. 6, BOX C). Unfortunately, BDNF signaling was not studied in neurons deficient in SorCS1, precluding any conclusions as to whether the effect was reliant on this receptor. Better investigated is the function of SorCS1 in binding of the presynaptic cell adhesion molecules Neurexins, and the regulation of synapse structure. Decreased levels of synaptic membrane proteins like Neurexins [333–335] and enlargement of early endosomes [336] have been proposed as novel biomarkers for AD diagnosis. The trans-binding of Neurexins expressed on the presynapse to Neuroligins that are present at the postsynapse, is required for synaptogenesis and synaptic stabilization. However, Neurexins can also bind AβO, which disrupts the interaction with Neuroligins, leading to severe damages of excitatory synapses [337, 338]. Recently, Joris de Wit’s group described that SorCS1 is a key sorting molecule regulating the axonal-dendritic polarization of synaptic proteins, a critical feature for neuronal wiring and synaptic plasticity [133, 302]. They discovered that SorCS1 localizes into early and recycling endosomes, where it controls trafficking of Neurexin and AMPAR to the neuronal surface. Since SorCS1 expression must be tightly regulated, the overexpression and the loss of SorCS1 activity leads to perturbed sorting and shifted ratio in the localization of Neurexin-1α, AMPARs, Neuroligin, and other polarized synaptic adhesion molecules at the axonal and dendritic surfaces [133, 302] (Fig. 6, BOX F). The imbalance in synaptic proteins distribution is caused by impaired SorCS1 interaction with Rab11-family-interacting protein 5 (Rip11), which governs the transition from early endosomes to Rab11-positive recycling endosomes. The alterations translate into reduced glutamatergic and GABAergic neurotransmission in cortical layer 5, an area where neurons are substantially affected in AD [133]. This is in accordance with observation that fluctuations in Neurexin and AMPA receptor activity sways the balance between excitatory and inhibitory neurotransmission in AD [339]. Notably, GABAergic and AMPA receptor neurotransmission is compromised in AD patients, and such alterations are associated with the cognitive decline [340–342]. Therefore, the impairments in SorCS1-dependent APP catabolism, trafficking of adhesion molecules, neurotransmitter receptors and trophic receptors may jointly cause synaptic dysfunction, synapse loss and neurodegeneration in AD patients. Unfortunately, the molecular mechanisms of SorCS1 signaling are still largely unknown. Future studies should thus functionally address the spectra of SorCS1 isoforms in relations to its binding partners in order to fully understand the regulatory mechanisms directly or indirectly involved in AD pathogenesis.

### SorCS2 biology and its role in AD

**Fig. 7:**
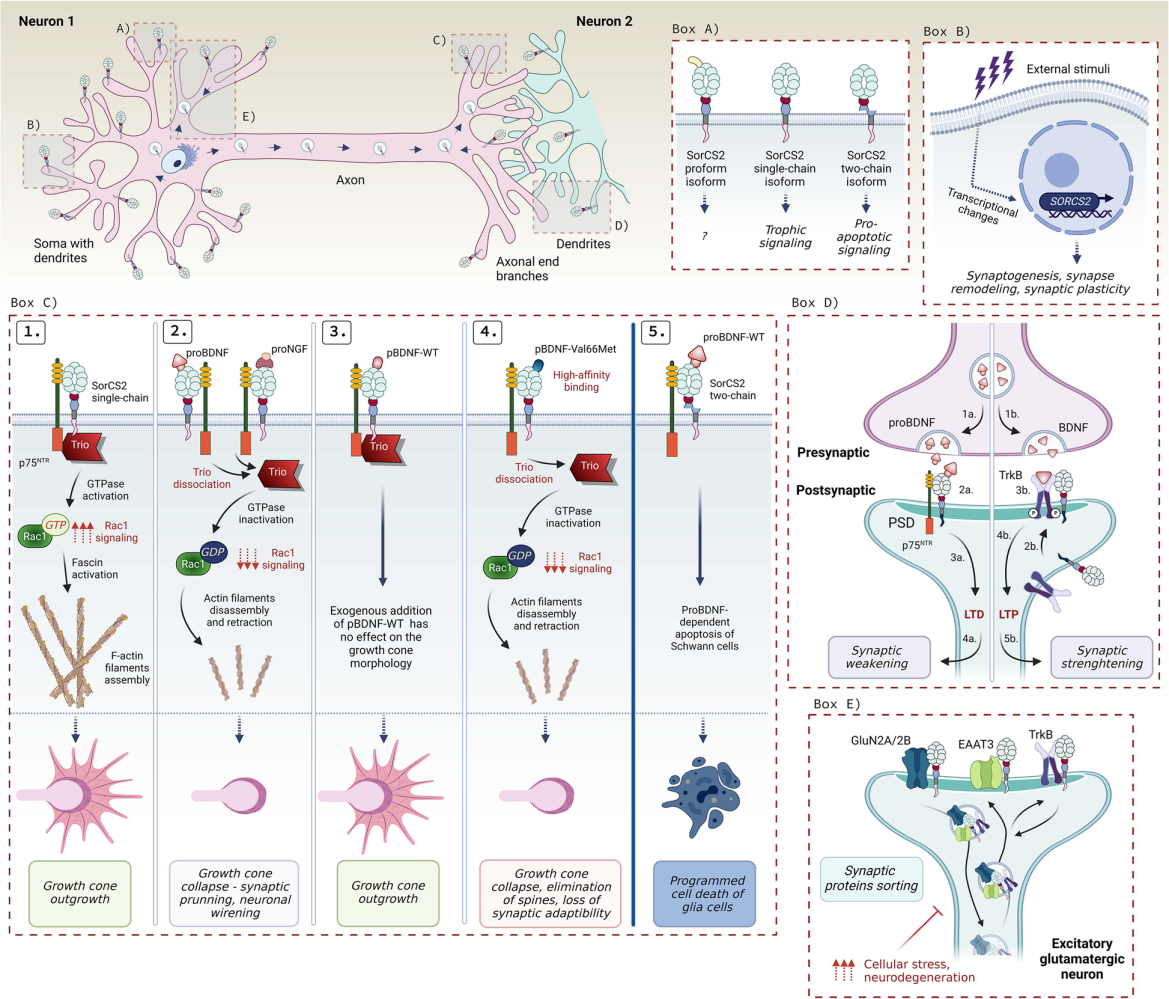
SorCS2 signaling in neuronal networks relevant for AD. SorCS2 is found in neural soma, dendrites and axons. Box A) SorCS2 exists in three isoforms that have different signaling profiles. SorCS2 is initially produced as a proform, which can be cleaved by Furin from its propeptide, giving rise to a single-chain receptor. The single-chain can be further cleaved within the leucine-rich domain, producing a two-chain isoform. Box B) SorCS2 expression changes upon external stimuli, which affects synaptic plasticity. Box C) SorCS2 interactions with neurotrophins. *1.* SorCS2 single-chain binds p75^NTR^ and Trio, which mediates Rac1 and Fascin signaling. Fascin activation leads to F-actin filaments assembly and growth cone outgrowth. *2.* ProBDNF or proNGF binding to SorCS2 leads to dissociation of Trio causing Rac1 signaling inactivation, actin filaments disassembly and retraction, and grow cone collapse, which is important for synaptic pruning and neuronal wirening. *3.* Propeptide of BDNF-WT binding to SorCS2 does not affect the growth cone outgrowth. *4.* Propeptide of BDNF with Val66Met mutation exhibits high binding affinity to SorCS2, subsequently dissociating Trio, and inhibiting Rac1 signaling. This pathway promotes elimination of spines and loss of synaptic adaptability. *5.* SorCS2 two-chain binds p75^NTR^, which mediates proBDNF-dependent apoptosis. Box D) SorCS2 controls synaptic plasticity. Upon proBDNF release (*1a.*), SorCS2 mediates synaptic weakening (*4a.*) via its interaction with proBDNF and p75^NTR^ (*2a.*), which induces long-term potentiation (LTD; *3a.*). SorCS2 and the BDNF receptor TrkB are located outside of the postsynaptic density (PSD). Upon BDNF release (*1b.*), SorCS2 and TrkB relocate to the synapse (*2b.*) where they interact. TrkB binds BDNF, and undergoes phosphorylation and activation (*3b.*), subsequently inducing LTP (*4b.*) and synaptic strengthening (*5b.*). Box E) SorCS2 interacts with synaptic receptors GluN2A/2B, EAAT3, and TrkB at PSD of glutamergic neurons, and regulates their anterograde and retrograde trafficking. Impairments in these processes lead to increased cellular stress and neurodegeneration

SorCS2 was described by Rezgaoui et al. in 2001 as a gene dynamically expressed during mouse brain development, particularly in dorsal thalamus and midbrain floorplate [123]. In adulthood, SorCS2 is highly expressed in hippocampus (particularly in dentate gyrus, CA2 and CA3 regions), piriform cortex, and in striatal medium spiny neurons [121, 123, 343, 344]. It is localized mostly in somatic vesicles, but also in neurites, dendritic spines, filopodia-rich projections, and in the growth cone of projecting axons [36, 54, 55, 132]. Like other VPS10p-D receptors, SorCS2 engages in cellular trafficking and signaling controlled by its expression and the presence of co-receptors and ligands. Expression of SorCS2 can be altered by external stimuli, which was demonstrated by deep brain stimulation of subthalamic nucleus [127] (Fig. 7, BOX B). This feature is crucial for acute morphological responses, such as neurite regrowth, synaptogenesis as well as disassembling of synapses, and the control of synaptic plasticity [55]. Nykjaer’s group showed that SorCS2 exists in three active isoforms: an immature proform, and mature single- and two-chain variants. These isoforms are generated by sequential proteolytic cleavage, and are presented uniquely by neurons and glial cells during intercellular communication in order to activate either trophic (single-chain) or pro-apoptotic (two-chain) signaling [132] (Fig. 7, BOX A). The receptor can also form homodimers which may adopt at least two distinct conformations that is controlled by binding of its ligands [135, 303]. Potentially, this might modify its interaction with co-receptors or cytosolic adaptor proteins, and thus regulate SorCS2-dependent signaling and sorting. Among its many functions, SorCS2 facilitates intracellular sorting and distribution of synaptic proteins, and protects neurons from oxidative stress and neuronal death [344, 345]. It also transduces signals that mediate neuronal remodeling and synaptic plasticity [54, 55, 132]. As such, it has been genetically linked to a number of psychiatric and neurodegenerative disorders [344]. Human genetic studies uncovered more than 18 SNPs in *SORCS2* that were associated to AD [83, 84]. They also observed epistatic interactions with SorCS1 and SorCS3 in AD patients [84]. Despite the genetic data and established functions in signaling and neurotransmission, SorCS2 is the least studied VPS10p-D receptor when it comes to AD. In the following paragraphs we will discuss the possible molecular and functional implications of SorCS2 in AD pathogenesis.

#### SorCS2 in AD-related neurotrophin signaling and synaptic transmission

In the adult brain, the major function of SorCS2 is to govern synapse morphology, synaptic plasticity, and neurotrophin signaling; processes crucially involved in AD. SorCS2 binds and mediates intracellular sorting and synaptic localization of the NMDA receptor subunits GluN2A and GluN2B [344, 346]. Strikingly, mass spectrometry analysis of rat brains revealed that SorCS2 also interacts with Neurexin-1β [133]. These studies thus manifest a fundamental role of SorCS2 in synaptogenesis, synaptic proteome composition, and synapse stability. Importantly, SorCS2 might also regulate cellular responses to stress. Malik et al. reported that SorCS2 controls the functional expression of the excitatory amino acid transporter EAAT3 and protects neurons from oxidative stress, excitotoxicity, and neurodegeneration [345] (Fig. 7, BOX E). SorCS2 involvement in cellular fitness has been further demonstrated by Gospodinova et al. who showed that SorCS2 loss enhances the incidence of Topoisomerase II-dependent DNA double-strand breaks in hippocampal neurons, which subsequently reduced neuronal viability [347]. This is interesting since DNA breaks accompany aging, and are common in neurodegenerative conditions. Recent study by Chaves et al. suggested that DNA polymorphisms in *SORCS2* and other members of the VPS10p-D family are causative to the neurodegenerative disorder Huntington’s disease [348]. Indeed, SorCS2 has been functionally associated with Huntington’s disease via its interaction with mutated huntingtin protein and its impaired sorting [344]. Combined, there is a substantial evidence that SorCS2 might be involved in several aspects of neurodegenerative processes including its neuroprotective role against cellular stress.

Particularly well studied is SorCS2 function in signaling established by pro- and mature neurotrophins, which is illustrated in Fig. 7, BOX C. ProNGF and proBDNF bind to SorCS2 at the plasma membrane, and both single- and two-chain SorCS2 variants can be in complex with a proneurotrophin ligand and p75^NTR^ [132], forming higher-order signaling assembly [135]. During neuronal development, the SorCS2-p75^NTR^ complex is essential for proBDNF and proNGF to induce growth cone collapse of extending neurites thereby controlling neuronal wiring and connectivity. Notably, this activity is specifically reliant on the single-chain isoform of SorCS2. In contrast, two-chain SorCS2 is required for pro-NTs-dependent induction of glial cells apoptosis [132]. Binding of the cytosolic guanine-nucleotide exchange factor Trio to SorCS2-p75^NTR^ is required for the retraction of extending axon [55]. As demonstrated for proNGF, the engagement of the SorCS2-p75^NTR^ complex with a proNT displaces Trio and downregulates Rac1 signaling while activating Protein kinase C. Jointly, these events destabilize actin filaments, and lead to impaired filopodia retraction and subsequent growth cone collapse [55]. If defective, such impairments perturb neuronal connectivity and synapse function, causing increased neuronal vulnerability and neuronal death in the aging brain [132, 349–351].

The biological function of single-chain SorCS2 is not limited to the neurodevelopmental stage. In the postnatal hippocampus, binding of proBDNF released from the presynapse towards the SorCS2-p75^NTR^ complex located in the postsynapse will induce long-term depression (LTD) and synaptic weakening [54]. Notably, SorCS2 can engage with TrkB to enable the local recruitment of TrkB from extrasynaptic sites to PSD95-positive domains, which is necessary for induction of long-term potentiation (LTP) and synaptic strengthening by mature BDNF [54, 352] (Fig. 7, BOX D). Studies by Mizui et al. further revealed that cleaved propeptide of BDNF (pBDNF) can be independently secreted in the activity-dependent manner to facilitate LTD [353]. Strikingly, the propeptide containing the naturally occurring pBDNF-Val66Met mutation, which is associated with memory impairment and predicts cognitive decline in AD patients [37–39], binds to SorCS2 with a greater affinity than pBDNF-WT [139]. While SorCS2 binding to pBDNF-WT increases LTD [353], the interaction with the pBDNF-Val66Met abolishes LTD as it induces Rac1 downregulation followed by the loss of Trio-positive dendritic spines, and acute growth cone retraction [36, 139] (Fig. 7, BOX C). This regulation is followed by reduced dendritic spine density in CA1 region of hippocampus, altered prelimbic projections and maturation of fear extinction circuitry [36, 139, 353].

So far, SorCS2 has not been studied in context of the amyloidogenic pathways. However, its multiple activities in neuronal wiring, synapse dynamics, neurotransmission, and synaptic plasticity governed by proNT-SorCS2-p75^NTR^ and BDNF-SorCS2-TrkB signaling and by trafficking of neurotransmitter receptors and transporters, clearly demonstrate the critical role of SorCS2 in neuronal integrity and functionality. Although disturbances in SorCS2 increase neuronal vulnerability [344] and may precipitate earlier AD onset and propel disease progression for those at risk, functional studies are required to address whether SorCS2 may also directly impact on amyloid and Tau biology.

### SorCS3 biology and its role in AD

Despite very limited knowledge about the molecular function of SorCS3, there is substantial evidence for its implications in AD. Similarly to SorCS1 and -2, SorCS3 exhibits spatiotemporal expression during development. In the adulthood, SorCS3 is expressed across the brain, with the highest expression in the CA1 region of hippocampus and in cerebral cortex [121, 122]. SorCS3 mostly localizes at the plasma membrane where it binds its ligands. Using single-particle cryogenic electron microscopy at neutral pH, Dong et al. recently suggested that human SorCS3 exists mostly as a dimer since they observed 79% of dimer particles compared to 21% of monomer particles [156]. Interestingly, SorCS3 monomer contained 10CC domain in different orientation than the dimers. They detected three different conformations of the dimers, and suggested that SorCS3 exhibits dynamic conformational changes, which differed from SorLA and SorCS2, especially in the possible ligand-binding features. However, this structural study is still quite preliminary.

**Fig. 8:**
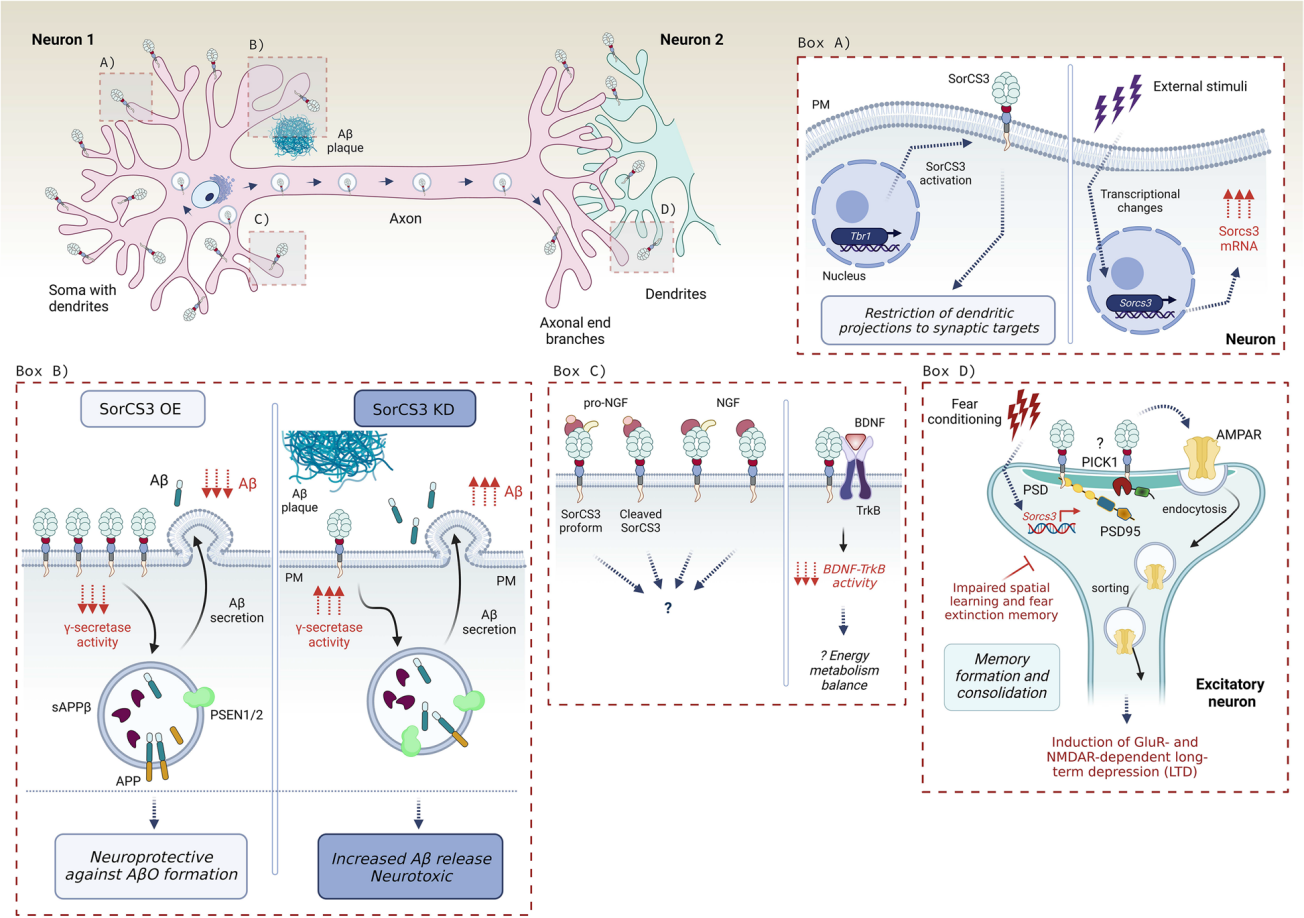
SorCS3 localization and AD-related signaling. SorCS3 predominantly displays somatodendritic localization, especially at the plasma membrane where it binds its ligands. It is also found in axons but at minor extent. Box A) SorCS3 is a downstream effector of transcription factor Tbr1, which restricts dendritic projections to their synaptic targets during development (*left panel*). Moreover, SorCS3 transcription is inducible upon various external stimuli such as LTP, seizures or fear conditioning (*right panel*). Box B) SorCS3 signaling is involved in amoyloidogenic pathway. SorCS3 overexpression (OE) reduces γ-secretase activity and APP processing resulting in lower production and secretion of Aβ. SorCS3 gain of function is thus neuroprotective against accumulation of Aβ oligomers. In contrast, SorCS3 downregulation (KD) results in an enhanced γ-secretase activity and thus larger formation and secretion of Aβ into the extracellular space. Thus, SorCS3 loss of function is neurotoxic as it promotes the amyloidogenic pathway. However, molecular mechanisms behind these observations are unknown. Box C) SorCS3 can signal upon its ligands’ binding already as a proform. Both SorCS3 isoforms bind proNGF and NGF, however the functional consequences remain unknown. SorCS3 can bind TrkB, by which it blunts BDNF-mediated TrkB activation. It is believed that this signaling axis is important for energy metabolism balance. Box D) SorCS3 signaling at the synapse is critical for memory formation and consolidation of excitatory neurons. SorCS3 resides at the postsynaptic side of excitatory neurons where it interacts with PSD95 and probably with PICK1 (the interaction was shown only in the HEK293 cells), which was suggested to mediate the endocytosis and sorting of AMPA receptors, a critical step for GluR- and NMDAR-dependent long-term depression (LTD), spatial learning and fear extinction memory. Notably, *Sorcs3* expression is upregulated in engram neurons during memory formation

SorCS3 interacts with its binding partners even prior to its maturation by propeptide cleavage [136]. The SorCS3 cytoplasmic tail is responsible for its intracellular trafficking as it navigates SorCS3 into dendrites and to a lower extent also into axons [122] (Fig. 8). The major function of SorCS3 is to control synaptic structure and function, via binding of scaffold proteins and controlling glutamate receptor trafficking [354, 355]. However, SorCS3 involvement in neurotrophin signaling has been proposed [136, 144]. In 2013, Reitz et al. described for the first time that *SORCS3* is genetically associated to AD (12 SNPs). An epistatic analysis of the AD cohort revealed a strong interaction of *SORCS3* mutations with those in *SORCS2* (24 SNP pairs) and *SORCS1* (8 SNP pairs). These mutations were all located in introns 1 and 2, thus the introns contiguous to the exons encoding the ligand-binding VPS10 domain (similarly to SNPs found in *SORCS1* and *SORCS2*) [84]. Further, a recent whole genome sequence analysis of a multiethnic cohort comprising 11,000 women, found a strong genome-wide significant association between *SORCS3* and dementia with an odds-ratio of no less than 4.4. Transcriptome analysis confirmed that SorCS3 expression is indeed substantially decreased in the context of AD [356]. These studies found that SorCS3 is consistently downregulated in AD [84, 357]. Recently, the Psychiatric Genomic Consortium identified *SORCS3* as a shared top-risk gene across 8 different psychiatric disorders highlighting the pleiotropic though unclear function of SorCS3 in healthy and diseased human brain [90]. This is interestingly given a GWAS analysis from a Han Chinese cohort of AD patients with major depressive disorder further identified 675 SNPs in *SORCS3* gene, thus bridging these two commonly comorbid disorders [358, 359] with one risk factor [90, 360]. In the following paragraphs, we will discuss the direct and indirect functional links of SorCS3 to AD.

#### SorCS3 interactions in amyloidogenic cascade

Ni et al. found a differential expression of *SORCS3* in AD brains when comparing hippocampus, entorhinal cortex, frontal cortex, and temporal cortex [360]. Additionally, Reitz et al. showed that expression of three *SORCS3* exons (exons 10, 17 and 21) is reduced in the amygdala of AD patients. In contrast, the occipital lobe and cerebellum that also express *SORCS3* were unaffected by the disease [84]. The authors proposed that SorCS3 is involved in the amyloidogenic pathway by regulating the activity of γ-secretase and APP processing. Indeed, overexpression of SorCS3 leads to downregulation of γ-secretase activity, whereas SorCS3 knock-down causes an increase in γ-secretase processing of APP [84] (Fig. 8, BOX B). A recent study by Hermey et al. investigated SorCS3 expression in specific brain regions during healthy aging and after amyloidosis. They compared SorCS3 expression in aging wild-type mice with APP/PS1 mice that model AD, and develop Aβ plaque within the first year. They found that amyloid plaques formation, but not aging, reduces SorCS3 expression in the frontal cerebral cortex, with no change in the hippocampus [101]. These data suggest that the AD pathogenesis is associated with impaired SorCS3 activity in a brain region-specific manner.

#### SorCS3 in AD-related neurotrophin signaling and synaptic transmission

During development, SorCS3 acts as a downstream effector of Transcription factor T-box brain1 (Tbr1) expression that restricts dendritic projections towards their synaptic targets [361] (Fig. 8, BOX A). Immature as well as cleaved SorCS3 receptor can bind proNGF and NGF in vitro but the functional consequences have not been explored [136]. However, a recent paper by Zhang et al. suggests that SorCS3, as opposed to Sortilin and SorCS2, prevents p75^NTR^ signaling by increasing its internalization and transport to lysosomes for degradation [362]. The implications for proNGF signaling, however, were not studied. As proposed for SorCS1, SorCS3 can physically interact with TrkB which abrupts BDNF-dependent TrkB activity in the hypothalamus, and likely contributes to energy metabolism balance [144] (Fig. 8, BOX C). Unfortunately, as for SorCS1, the functional impact and mechanistic insight of this crosstalk are not clear since only mice devoid in both *Sorcs1* and *Sorcs3* were studied. However, TrkB was enriched in synaptosomes in brain extracts of the double *Sorcs1; Sorcs3* knockout mice while it was reduced in sorting vesicles. Accordingly, BDNF-induced TrkB phosphorylation was stronger in cultured cortical neurons from mice lacking both receptors [144]. Hence, the VPS10p-D receptors jointly modulate all key functions of BDNF signaling. Strikingly, they do so by controlling specific steps in TrkB trafficking (for Sortilin also demonstrated with TrkA and TrkC [142]). Sortilin enables the anterograde transport of TrkB from the soma along axons to the synapse [142]. SorLA can do the same but it also facilitates its retrograde transport, potentially in signaling endosomes [143]. Locally at the synapse, SorCS2 takes over the TrkB from Sortilin and SorLA to target it from extrasynaptic sites to postsynaptic densities [54]. In marked contrast to the rest of the family, SorCS1 and/or SorCS3 impedes BDNF signaling by removing TrkB from the synapse [144]. In all, the receptors jointly control and fine-tune BDNF signaling; a key regulator of neuronal integrity and vulnerability.

Aside from these observations, SorCS3 relations to neurotrophic signaling and plasticity remain unknown. Recent studies highlighted SorCS3 as an important regulator of synaptic events in excitatory neurons but with only minimal impact on the inhibitory input [354, 355]. Moreover, SorCS3 deficient mice exhibit loss of NMDAR and mGluR-dependent LTD in the hippocampal CA1 region, while LTP is preserved. These mice have no sign of brain atrophy but respond to repetitive stimulation with synaptic facilitation and reduced synaptic depression; a phenotype that progressively worsened as the animals aged [355]. This is particularly interesting as SorCS3 expression is regulated by neuronal activity including LTP [124] and seizures [125]. Fear conditioning enhances *Sorcs3* expression by no less than 170-fold in hippocampal engram cells arguing that SorCS3 signaling and/or sorting contributes to memory formation and consolidation [126]. Indeed, studies using S*orcs3 KO* mice reported that the animals exhibited impaired spatial learning but increased fear extinction [354]. Interestingly, protein levels of PSD95, AMPA receptors, NMDA receptors, mGluR5, p75^NTR^, and TrkB are not changed in postsynaptic density fraction extracted from hippocampi of *Sorcs3 KO* mice [354]*.* However, Breiderhoff et al. showed that SorCS3 crosstalks with some synaptic proteins. SorCS3 localizes at the postsynaptic density where it binds PSD95 via its putative PDZ domain binding motif in its cytoplasmic domain [354]. The authors also proposed that SorCS3 interacts with an adaptor molecule PICK1 by which it controls the necessary removal of the AMPA receptors from the postsynaptic side [354], similarly to SorCS1 [133]. This study was further supported by electrophysiological observations that LTD deficiency in CA1 region is age-dependent, and that the loss of SorCS3 impacts on AMPA receptors mobility [355]. SorCS3 actions at postsynapse of excitatory neurons during memory formation are depicted in (Fig. 8, BOX D).

Given the genetic association to AD and the important functions of SorCS3 in APP processing, synaptic transmission, and synaptic retraction of excitatory neurons involved in learning and memory, additional studies are merited to examine its role in the context of AD. SorCS3 remains the least studied of the VPS10 p-D receptors. Future studies should thus: 1) Determine SorCS3 binding partners in amyloidogenic pathways and synaptic events, and 2) describe the molecular mechanisms by which SorCS3 regulates synaptic transmission in the healthy and AD brain. Moreover, SorCS3 possible interaction with the neurotrophic signaling deserves more attention as the rest of this receptor family serves critical functions in neurotrophin-dependent neuronal survival and death.

### Therapeutic perspectives of VPS10p-D receptors in AD

It remains unclear whether certain neurons in AD brains become vulnerable mostly due to intrinsic or extrinsic factors. The largest therapeutic potential used to lie within blocking the amyloidogenic cascade, and/or dissolving the amyloid plaques and Tau-containing tangles. Unfortunately, the current strategies are inefficient and do not stop the disease progression. In this review, we illustrated that the AD pathogenesis has been tightly connected with impaired activity of VPS10p-D receptors in neurons. However, not much is known about VPS10p-D receptors signaling in the context of intercommunication between cell types such as microglia-neuron, a feature largely implicated in AD. Microglia are non-neuronal cells that support neurons by secreting trophic factors and performing phagocytic clearance during synapse remodeling and tissue repair. Together with astrocytes they are responsible for ApoE production which controls the deposition and clearance of Aβ peptides. With AD progression and increased Aβ accumulation, activated microglia compromise their phagocytic abilities, alter their secretome, and mediate chronic neuroinflammation leading to synaptic loss and AD neurodegeneration. The possession of ApoE4 allele triggers and sustains the microglia-driven neuroinflammation. Enhancing phagocytosis and decreasing neuroinflammation in AD patients has become a new therapeutic target, even though this cell communication remains a black box [363, 364]. Since neuronal VPS10p-D receptors closely and diversely interact with ApoE, Aβ and synaptic proteins, understanding the molecular mechanisms involved in this intercellular communication is key when targeting AD pathogenesis.

The importance of VPS10p-D receptors in neurotrophic signaling during CNS development and homeostasis also possesses a high translational value. Current advances in regenerative medicine show that reactivating the developmental features related to neurotrophic signaling during trauma and neuroinflammation promotes healing and improves the cognitive decline as it provides substantial functional recovery [365]. The potential of cell replacement therapies in AD represents a symptomatic solution with limited efficacy due to the broad spread of neurodegeneration. However, recent studies uncovered that elevating the neurotrophin levels (such as BDNF, NGF and GDNF) in AD mice supports neuronal integration of grafted cells into the circuits, which improves the cognitive deficits [366, 367]. Recent work from Choi et al. combined pharmacological and genetic approaches to elevate BDNF levels in the AD mouse model (5xFAD) to trigger adult neurogenesis in hippocampus. Indeed, this strategy improved cognitive performance of the mice [368]. Similarly, conditional BDNF delivery provided by astrocytes rescued dendrite outgrowth, neuronal connectivity and memory deficits in another AD study [369]. As boosting the BDNF concentration increases the neuroprotective levels of SorLA [137], such therapy could provide a relief of the AD symptoms.

Neurotrophins and Trk receptors facilitate a vast spectrum of functions in the CNS, and thus they represent a difficult therapeutic target. We therefore suggest that novel pharmacological interventions should instead aim for their binding partners, the VPS10p-D receptors, because they control more defined processes. Promoting the interaction between Trk receptors and VPS10p-D receptors might have positive effects on neurotrophin-dependent cell survival. *Sortilin KO* mice are resistant to acute and senescent neurodegeneration [53]. Therefore, drug discovery of Sortilin antagonists that block the ability of Sortilin interaction with p75^NTR^ also represents a novel way of intervention against progressive cell death in AD. This approach has been proven functional for treating another neurodegenerative disease, frontotemporal dementia (FTD) which exhibits impaired Sortilin-progranulin signaling axis [272, 370]. Currently, there is an ongoing FTD immunotherapy entering Phase 3 clinical trials that uses a monoclonal anti-human Sortilin antibody called AL001 (Identifier: NCT04374136) [371]. If approved, this treatment could be expanded to AD patients.

Indeed, the various molecular interactions of VPS10p-D receptors in AD-related cell communication, their functional involvement in amyloidogenic pathways, and most importantly, their genetic links to AD identify these receptors as highly promising clinical targets for future advances in AD diagnostics and therapy.

## Conclusions

Several human genetic and functional studies have repeatedly linked the VPS10p-D receptor family to AD, and to its pathophysiological features including accumulation of extracellular Aβ. Importantly, simultaneous deregulation of multiple members of this family has an epistatic effect on the AD onset. For example, both SorLA, Sortilin, and SorCS1 play a major role in controlling APP trafficking, but they guide the precursor to different subcellular destinies. Similarly, SorLA directly interacts with Aβ, whereas the other receptors influence amyloid accumulation by acting in its metabolic processing. Impaired signaling of VPS10p-D proteins also leads to altered ratio between the amount of immature and mature neurotrophic factors thereby altering synaptic plasticity and neuronal cell fate, common features of AD. Last but not least, VPS10p-D receptor family has been linked to major depressive disorder, prion-like infections, and diabetes mellitus, thus diseases that often accompany AD diagnosis. At present, we still have poor understanding about the precise molecular mechanisms by which these receptors signal in healthy and AD brains. Nevertheless, it is indeed clear that the versatility of VPS10p-D receptors and their broad molecular interactions with AD-related pathways can help explaining the AD diversity and its comorbidities. We therefore expect that this exciting field will soon escalate, and lead to uncovering many new diagnostic and therapeutic possibilities for AD patients in the future, in particular with focus on SorLA, Sortilin, and SorCS1/3.

## Data Availability

Not applicable.

## Abbreviations

AD: Alzheimer’s disease
ADAM9/10/17: Alpha secretase ADAM metallopeptidase domain 9/10/17
AMPAR: α-amino-3-hydroxy-5-methyl-4-isoxazolepropionic acid AMPA glutamate receptor
AP-1/AP-2: Adaptor protein complex 1/ and 2
APOE: Apolipoprotein E
APP: Amyloid-beta precursor protein
Aβ: Amyloid beta
AβO: Cytotoxic amyloid beta oligomers
BACE1: Beta-site amyloid precursor protein cleaving enzyme secretase 1
BDNF: Brain-derived neurotrophic factor
BDNF-Val66Met: Brain-derived neurotrophic factor carrying a substitution of Valine with Methionine
BFCN: Basal forebrain cholinergic neuron
BMP: Bone morphogenetic protein
CASP3: Caspase 3
CNS: Central nervous system
CSF: Cerebral spinal fluid
EAAT3: Excitatory amino acid transporter 3
EOAD: Early-onset Alzheimer’s disease
EphA4: Ephrin type-A receptor 4
FTD: Frontotemporal dementia
GDNF: Glial cell line-derived neurotrophic factor
GFRα1: GDNF Family Receptor Alpha 1
GGA: Golgi-localized gamma-ear-containing ARF-binding protein
GluN2A/2B: Glutamate (NMDA) receptor subunit 2A/2B
GLUT4: Glucose transporter 4
GRN: Granulin
GSK3β: Glycogen synthase kinase-3 beta
GWAS: Genome-wide association studies
HSP12A: Heat shock protein 12A
iPS cells: Induced pluripotent stem cells
IR: Insulin receptor
LDL: Low-density lipoprotein
LOAD: Late-onset Alzheimer’s disease
LTD: Long-term depression
LTP: Long-term potentiation
mGluR5: Metabotropic glutamate receptor 5
NGF: Nerve growth factor
NMDAR: N-methyl-D-aspartate receptor
NT3/4: Neurotrophin 3/4
p75^NTR^: Neurotrophin receptor denoted p75
PAK 1-3: Rac-p21-activated kinases 1 to 3
pBDNF: Propeptide of BDNF (after cleavage of proBDNF)
PCSK9: Proprotein convertase subtilisin/kexin type 9
PGRN: Progranulin
PICK1: Protein interacting with C kinase 1
PNS: Peripheral nervous system
proBDNF: Precursor of brain-derived neurotrophic factor
proNGF: Precursor of nerve growth factor
proNTs: Proneurotrophins
PrP^C^: Prion protein
PrP^Sc^: Scrapie isoform of prion protein
PSEN1/2: Presenilin 1/2
pTau: Hyperphosphorylated Tau
Rab7/11: Ras-related protein 7/11
Rac1: Rac Family Small GTPase 1
RET: Ret proto-oncogene
sAPP: Soluble Amyloid-beta precursor protein
SNP: Single nucleotide polymorphism
SNX-1: Sorting nexin 1
SNX-27: Sorting nexin 27
Sorl1/SorLA/LR11: Sortilin-related receptor 1
*SORL1-30B*: Splice variant of *SORL1* containing novel exon 30B
*SORL1-38b*: Splice variant of *SORL1* containing inclusion of additional exon 38b
Sort1: Sortilin
sSorLA: Soluble SorLA
T2DM: Type 2 Diabetes Mellitus
TDP-43: TAR DNA-binding protein 43
TGFβ: Transforming growth factor beta
TGN: Trans-Golgi network
TNFα: Tumor necrosis factor alpha
TrkA/B/C: Tropomyosin receptor tyrosine kinases A/B/C
VPS10p-D: Vacuolar protein sorting 10p-Domain
VPS35: Vacuolar protein sorting-associated protein 35
WAT: White adipose tissue

## References

[1] 2020 Alzheimer's disease facts and figures. Alzheimer's & Dementia 2020;16(3):391-460. 10.1002/alz.12068. 10.1002/alz.12068 32157811

[2] Scheltens P, De Strooper B, Kivipelto M, Holstege H, Chételat G, Teunissen CE, Cummings J, van der Flier WM (2021). Alzheimer’s disease. Lancet.

[3] Wang J, Gu BJ, Masters CL, Wang Y-J (2017). A systemic view of Alzheimer disease — insights from amyloid-β metabolism beyond the brain. Nat Rev Neurol.

[4] Busche MA, Hyman BT (2020). Synergy between amyloid-β and tau in Alzheimer’s disease. Nat Neurosci.

[5] Panza F, Lozupone M, Logroscino G, Imbimbo BP (2019). A critical appraisal of amyloid-β-targeting therapies for Alzheimer disease. Nat Rev Neurol.

[6] Caselli RJ, Knopman DS, Bu G (2020). An agnostic reevaluation of the amyloid cascade hypothesis of Alzheimer’s disease pathogenesis: The role of APP homeostasis. Alzheimers Dement.

[7] Rice HC, de Malmazet D, Schreurs A, Frere S, Van Molle I, Volkov AN, Creemers E, Vertkin I, Nys J, Ranaivoson FM (2019). Secreted amyloid-beta precursor protein functions as a GABABR1a ligand to modulate synaptic transmission.

[8] Gao L, Zhang Y, Sterling K, Song W (2022). Brain-derived neurotrophic factor in Alzheimer’s disease and its pharmaceutical potential. Transl Neurodegener.

[9] Mitre M, Mariga A, Chao MV (2017). Neurotrophin signalling: novel insights into mechanisms and pathophysiology. Clin Sci (London, England: 1979).

[10] Fahnestock M, Shekari A (2019). ProNGF and Neurodegeneration in Alzheimer’s Disease. Front Neurosci.

[11] Gatz M, Reynolds CA, Fratiglioni L, Johansson B, Mortimer JA, Berg S, Fiske A, Pedersen NL (2006). Role of genes and environments for explaining Alzheimer disease. Arch Gen Psychiatry.

[12] Hoogmartens J, Cacace R, Van Broeckhoven C (2021). Insight into the genetic etiology of Alzheimer’s disease: A comprehensive review of the role of rare variants. Alzheimers Dement (Amst).

[13] Jansen IE, Savage JE, Watanabe K, Bryois J, Williams DM, Steinberg S, Sealock J, Karlsson IK, Hägg S, Athanasiu L (2019). Genome-wide meta-analysis identifies new loci and functional pathways influencing Alzheimer’s disease risk. Nat Genet.

[14] Bellenguez C, Küçükali F, Jansen IE, Kleineidam L, Moreno-Grau S, Amin N, Naj AC, Campos-Martin R, Grenier-Boley B, Andrade V (2022). New insights into the genetic etiology of Alzheimer’s disease and related dementias. Nat Genet.

[15] Kunkle BW, Grenier-Boley B, Sims R, Bis JC, Damotte V, Naj AC, Boland A, Vronskaya M, van der Lee SJ, Amlie-Wolf A (2019). Genetic meta-analysis of diagnosed Alzheimer’s disease identifies new risk loci and implicates Aβ, tau, immunity and lipid processing. Nat Genet.

[16] Ridge PG, Hoyt KB, Boehme K, Mukherjee S, Crane PK, Haines JL, Mayeux R, Farrer LA, Pericak-Vance MA, Schellenberg GD, Kauwe JSK (2016). Assessment of the genetic variance of late-onset Alzheimer’s disease. Neurobiol Aging.

[17] Nixon RA (2017). Amyloid precursor protein and endosomal-lysosomal dysfunction in Alzheimer’s disease: inseparable partners in a multifactorial disease. FASEB J.

[18] Wang X, Huang T, Bu G, Xu H (2014). Dysregulation of protein trafficking in neurodegeneration. Mol Neurodegener.

[19] Liu CC, Liu CC, Kanekiyo T, Xu H, Bu G (2013). Apolipoprotein E and Alzheimer disease: risk, mechanisms and therapy. Nat Rev Neurol.

[20] Farrer LA, Cupples LA, Haines JL, Hyman B, Kukull WA, Mayeux R, Myers RH, Pericak-Vance MA, Risch N, van Duijn CM (1997). Effects of age, sex, and ethnicity on the association between apolipoprotein E genotype and Alzheimer disease. A meta-analysis. APOE and Alzheimer Disease Meta Analysis Consortium. JAMA.

[21] O’Donoghue MC, Murphy SE, Zamboni G, Nobre AC, Mackay CE (2018). APOE genotype and cognition in healthy individuals at risk of Alzheimer’s disease: A review. Cortex.

[22] Deane R, Sagare A, Hamm K, Parisi M, Lane S, Finn MB, Holtzman DM, Zlokovic BV (2008). apoE isoform-specific disruption of amyloid beta peptide clearance from mouse brain. J Clin Investig.

[23] Dorey E, Bamji-Mirza M, Najem D, Li Y, Liu H, Callaghan D, Walker D, Lue LF, Stanimirovic D, Zhang W (2017). Apolipoprotein E Isoforms Differentially Regulate Alzheimer’s Disease and Amyloid-beta-Induced Inflammatory Response in vivo and in vitro. J Alzheimers Dis.

[24] Nuriel T, Peng KY, Ashok A, Dillman AA, Figueroa HY, Apuzzo J, Ambat J, Levy E, Cookson MR, Mathews PM, Duff KE (2017). The Endosomal-Lysosomal Pathway Is Dysregulated by APOE4 Expression in Vivo. Front Neurosci.

[25] Nuriel T, Angulo SL, Khan U, Ashok A, Chen Q, Figueroa HY, Emrani S, Liu L, Herman M, Barrett G (2017). Neuronal hyperactivity due to loss of inhibitory tone in APOE4 mice lacking Alzheimer’s disease-like pathology. Nat Commun.

[26] Shi Y, Yamada K, Liddelow SA, Smith ST, Zhao L, Luo W, Tsai RM, Spina S, Grinberg LT, Rojas JC (2017). ApoE4 markedly exacerbates tau-mediated neurodegeneration in a mouse model of tauopathy. Nature.

[27] Chen Y, Durakoglugil MS, Xian X, Herz J (2010). ApoE4 reduces glutamate receptor function and synaptic plasticity by selectively impairing ApoE receptor recycling. Proc Natl Acad Sci USA.

[28] Conroy JN, Coulson EJ (2022). High-affinity TrkA and p75 neurotrophin receptor complexes: A twisted affair. J Biol Chem.

[29] Scott-Solomon E, Kuruvilla R (2018). Mechanisms of neurotrophin trafficking via Trk receptors. Mol Cell Neurosci.

[30] Phillips HS, Hains JM, Armanini M, Laramee GR, Johnson SA, Winslow JW (1991). BDNF mRNA is decreased in the hippocampus of individuals with Alzheimer’s disease. Neuron.

[31] Ginsberg SD, Malek-Ahmadi MH, Alldred MJ, Chen Y, Chen K, Chao MV, Counts SE, Mufson EJ (2019). Brain-derived neurotrophic factor (BDNF) and TrkB hippocampal gene expression are putative predictors of neuritic plaque and neurofibrillary tangle pathology. Neurobiol Dis.

[32] Lee J, Fukumoto H, Orne J, Klucken J, Raju S, Vanderburg CR, Irizarry MC, Hyman BT, Ingelsson M (2005). Decreased levels of BDNF protein in Alzheimer temporal cortex are independent of BDNF polymorphisms. Exp Neurol.

[33] Hock C, Heese K, Hulette C, Rosenberg C, Otten U (2000). Region-specific neurotrophin imbalances in Alzheimer disease: decreased levels of brain-derived neurotrophic factor and increased levels of nerve growth factor in hippocampus and cortical areas. Arch Neurol.

[34] Girotra P, Behl T, Sehgal A, Singh S, Bungau S. Investigation of the molecular role of brain-derived neurotrophic factor in Alzheimer's disease. J Mol Neurosci. 2022;72(2):173–86. 10.1007/s12031-021-01824-8. 10.1007/s12031-021-01824-8 34424488

[35] Notaras M, van den Buuse M (2020). Neurobiology of BDNF in fear memory, sensitivity to stress, and stress-related disorders. Mol Psychiatry.

[36] Giza JI, Kim J, Meyer HC, Anastasia A, Dincheva I, Zheng CI, Lopez K, Bains H, Yang J, Bracken C (2018). The BDNF Val66Met Prodomain Disassembles Dendritic Spines Altering Fear Extinction Circuitry and Behavior. Neuron.

[37] Boots EA, Schultz SA, Clark LR, Racine AM, Darst BF, Koscik RL, Carlsson CM, Gallagher CL, Hogan KJ, Bendlin BB (2017). BDNF Val66Met predicts cognitive decline in the Wisconsin Registry for Alzheimer’s Prevention. Neurology.

[38] Lim YY, Hassenstab J, Cruchaga C, Goate A, Fagan AM, Benzinger TL, Maruff P, Snyder PJ, Masters CL, Allegri R (2016). BDNF Val66Met moderates memory impairment, hippocampal function and tau in preclinical autosomal dominant Alzheimer’s disease. Brain.

[39] Lim YY, Rainey-Smith S, Lim Y, Laws SM, Gupta V, Porter T, Bourgeat P, Ames D, Fowler C, Salvado O (2017). BDNF Val66Met in preclinical Alzheimer’s disease is associated with short-term changes in episodic memory and hippocampal volume but not serum mBDNF. Int Psychogeriatr.

[40] Nagahara AH, Merrill DA, Coppola G, Tsukada S, Schroeder BE, Shaked GM, Wang L, Blesch A, Kim A, Conner JM (2009). Neuroprotective effects of brain-derived neurotrophic factor in rodent and primate models of Alzheimer’s disease. Nat Med.

[41] Blurton-Jones M, Kitazawa M, Martinez-Coria H, Castello NA, Müller FJ, Loring JF, Yamasaki TR, Poon WW, Green KN, LaFerla FM (2009). Neural stem cells improve cognition via BDNF in a transgenic model of Alzheimer disease. Proc Natl Acad Sci USA.

[42] Matrone C, Ciotti MT, Mercanti D, Marolda R, Calissano P (2008). NGF and BDNF signaling control amyloidogenic route and Abeta production in hippocampal neurons. Proc Natl Acad Sci USA.

[43] Wang ZH, Xiang J, Liu X, Yu SP, Manfredsson FP, Sandoval IM, Wu S, Wang JZ, Ye K (2019). Deficiency in BDNF/TrkB Neurotrophic Activity Stimulates δ-Secretase by Upregulating C/EBPβ in Alzheimer’s Disease. Cell Rep.

[44] Xiang J, Wang ZH, Ahn EH, Liu X, Yu SP, Manfredsson FP, Sandoval IM, Ju G, Wu S, Ye K (2019). Delta-secretase-cleaved Tau antagonizes TrkB neurotrophic signalings, mediating Alzheimer’s disease pathologies. Proc Natl Acad Sci USA.

[45] Xia Y, Wang ZH, Liu P, Edgington-Mitchell L, Liu X, Wang XC, Ye K (2021). TrkB receptor cleavage by delta-secretase abolishes its phosphorylation of APP, aggravating Alzheimer’s disease pathologies. Mol Psychiatry.

[46] Sleiman SF, Henry J, Al-Haddad R, El Hayek L, Abou Haidar E, Stringer T, Ulja D, Karuppagounder SS, Holson EB, Ratan RR (2016). Exercise promotes the expression of brain derived neurotrophic factor (BDNF) through the action of the ketone body β-hydroxybutyrate. eLife.

[47] Huang H, Li W, Qin Z, Shen H, Li X, Wang W (2021). Physical exercise increases peripheral brain-derived neurotrophic factors in patients with cognitive impairment: A meta-analysis. Restor Neurol Neurosci.

[48] Wang YL, Chio CC, Kuo SC, Yeh CH, Ma JT, Liu WP, Lin MT, Lin KC, Chang CP (2022). Exercise Rehabilitation and/or Astragaloside Attenuate Amyloid-beta Pathology by Reversing BDNF/TrkB Signaling Deficits and Mitochondrial Dysfunction. Mol Neurobiol.

[49] Tsai CL, Pai MC, Ukropec J, Ukropcová B (2019). Distinctive Effects of Aerobic and Resistance Exercise Modes on Neurocognitive and Biochemical Changes in Individuals with Mild Cognitive Impairment. Curr Alzheimer Res.

[50] Lee R, Kermani P, Teng KK, Hempstead BL (2001). Regulation of cell survival by secreted proneurotrophins. Science (New York, NY).

[51] Costa RO, Perestrelo T, Almeida RD (2018). PROneurotrophins and CONSequences. Mol Neurobiol.

[52] Nykjaer A, Lee R, Teng KK, Jansen P, Madsen P, Nielsen MS, Jacobsen C, Kliemannel M, Schwarz E, Willnow TE (2004). Sortilin is essential for proNGF-induced neuronal cell death. Nature.

[53] Jansen P, Giehl K, Nyengaard JR, Teng K, Lioubinski O, Sjoegaard SS, Breiderhoff T, Gotthardt M, Lin F, Eilers A (2007). Roles for the pro-neurotrophin receptor sortilin in neuronal development, aging and brain injury. Nat Neurosci.

[54] Glerup S, Bolcho U, Molgaard S, Boggild S, Vaegter CB, Smith AH, Nieto-Gonzalez JL, Ovesen PL, Pedersen LF, Fjorback AN (2016). SorCS2 is required for BDNF-dependent plasticity in the hippocampus. Mol Psychiatry.

[55] Deinhardt K, Kim T, Spellman DS, Mains RE, Eipper BA, Neubert TA, Chao MV, Hempstead BL (2011). Neuronal growth cone retraction relies on proneurotrophin receptor signaling through Rac. Sci Signal.

[56] Woo NH, Teng HK, Siao CJ, Chiaruttini C, Pang PT, Milner TA, Hempstead BL, Lu B (2005). Activation of p75NTR by proBDNF facilitates hippocampal long-term depression. Nat Neurosci.

[57] Fleitas C, Pinol-Ripoll G, Marfull P, Rocandio D, Ferrer I, Rampon C, Egea J, Espinet C (2018). proBDNF is modified by advanced glycation end products in Alzheimer’s disease and causes neuronal apoptosis by inducing p75 neurotrophin receptor processing. Mol Brain.

[58] Michalski B, Fahnestock M (2003). Pro-brain-derived neurotrophic factor is decreased in parietal cortex in Alzheimer’s disease. Brain Res Mol Brain Res.

[59] Mufson EJ, Counts SE, Fahnestock M, Ginsberg SD (2007). Cholinotrophic molecular substrates of mild cognitive impairment in the elderly. Curr Alzheimer Res.

[60] Peng S, Wuu J, Mufson EJ, Fahnestock M (2005). Precursor form of brain-derived neurotrophic factor and mature brain-derived neurotrophic factor are decreased in the pre-clinical stages of Alzheimer’s disease. J Neurochem.

[61] Fahnestock M, Garzon D, Holsinger RMD, Michalski B. Neurotrophic factors and Alzheimer's disease: are we focusing on the wrong molecule? J Neural Transm Suppl. 2002;(62):241-52. 10.1007/978-3-7091-6139-5_22. 10.1007/978-3-7091-6139-5_22 12456067

[62] Fahnestock M, Michalski B, Xu B, Coughlin MD (2001). The precursor pro-nerve growth factor is the predominant form of nerve growth factor in brain and is increased in Alzheimer’s disease. Mol Cell Neurosci.

[63] Peng S, Wuu J, Mufson EJ, Fahnestock M (2004). Increased proNGF levels in subjects with mild cognitive impairment and mild Alzheimer disease. J Neuropathol Exp Neurol.

[64] Mufson EJ, He B, Nadeem M, Perez SE, Counts SE, Leurgans S, Fritz J, Lah J, Ginsberg SD, Wuu J, Scheff SW (2012). Hippocampal proNGF signaling pathways and beta-amyloid levels in mild cognitive impairment and Alzheimer disease. J Neuropathol Exp Neurol.

[65] Counts SE, He B, Prout JG, Michalski B, Farotti L, Fahnestock M, Mufson EJ (2016). Cerebrospinal Fluid proNGF: A Putative Biomarker for Early Alzheimer’s Disease. Curr Alzheimer Res.

[66] Triaca V, Ruberti F, Canu N (2021). NGF and the Amyloid Precursor Protein in Alzheimer’s Disease: From Molecular Players to Neuronal Circuits. Adv Exp Med Biol.

[67] Cuello AC, Bruno MA, Bell KF (2007). NGF-cholinergic dependency in brain aging, MCI and Alzheimer’s disease. Curr Alzheimer Res.

[68] Mufson EJ, Kordower JH (1992). Cortical neurons express nerve growth factor receptors in advanced age and Alzheimer disease. Proc Natl Acad Sci USA.

[69] Ito S, Menard M, Atkinson T, Brown L, Whitfield J, Chakravarthy B (2016). Relative expression of the p75 neurotrophin receptor, tyrosine receptor kinase A, and insulin receptor in SH-SY5Y neuroblastoma cells and hippocampi from Alzheimer’s disease patients. Neurochem Int.

[70] Hu XY, Zhang HY, Qin S, Xu H, Swaab DF, Zhou JN (2002). Increased p75(NTR) expression in hippocampal neurons containing hyperphosphorylated tau in Alzheimer patients. Exp Neurol.

[71] Chakravarthy B, Menard M, Ito S, Gaudet C, Dal Pra I, Armato U, Whitfield J (2012). Hippocampal membrane-associated p75NTR levels are increased in Alzheimer’s disease. J Alzheimers Dis.

[72] Jiao SS, Bu XL, Liu YH, Wang QH, Liu CH, Yao XQ, Zhou XF, Wang YJ (2015). Differential levels of p75NTR ectodomain in CSF and blood in patients with Alzheimer’s disease: a novel diagnostic marker. Transl Psychiatry.

[73] Yao XQ, Jiao SS, Saadipour K, Zeng F, Wang QH, Zhu C, Shen LL, Zeng GH, Liang CR, Wang J (2015). p75NTR ectodomain is a physiological neuroprotective molecule against amyloid-beta toxicity in the brain of Alzheimer’s disease. Mol Psychiatry.

[74] Mufson EJ, Li JM, Sobreviela T, Kordower JH (1996). Decreased trkA gene expression within basal forebrain neurons in Alzheimer’s disease. NeuroReport.

[75] Dubus P, Faucheux B, Boissière F, Groppi A, Vital C, Vital A, Agid Y, Hirsch EC, Merlio JP (2000). Expression of Trk isoforms in brain regions and in the striatum of patients with Alzheimer’s disease. Exp Neurol.

[76] Hock C, Heese K, Müller-Spahn F, Hulette C, Rosenberg C, Otten U (1998). Decreased trkA neurotrophin receptor expression in the parietal cortex of patients with Alzheimer’s disease. Neurosci Lett.

[77] Ginsberg SD, Che S, Wuu J, Counts SE, Mufson EJ (2006). Down regulation of trk but not p75NTR gene expression in single cholinergic basal forebrain neurons mark the progression of Alzheimer’s disease. J Neurochem.

[78] Yi C, Goh KY, Wong LW, Ramanujan A, Tanaka K, Sajikumar S, Ibáñez CF (2021). Inactive variants of death receptor p75(NTR) reduce Alzheimer’s neuropathology by interfering with APP internalization. EMBO J.

[79] Qian L, Milne MR, Shepheard S, Rogers ML, Medeiros R, Coulson EJ (2019). Removal of p75 Neurotrophin Receptor Expression from Cholinergic Basal Forebrain Neurons Reduces Amyloid-β Plaque Deposition and Cognitive Impairment in Aged APP/PS1 Mice. Mol Neurobiol.

[80] Knowles JK, Rajadas J, Nguyen TV, Yang T, LeMieux MC, Vander Griend L, Ishikawa C, Massa SM, Wyss-Coray T, Longo FM (2009). The p75 neurotrophin receptor promotes amyloid-beta(1–42)-induced neuritic dystrophy in vitro and in vivo. J Neurosci.

[81] Triaca V, Sposato V, Bolasco G, Ciotti MT, Pelicci P, Bruni AC, Cupidi C, Maletta R, Feligioni M, Nisticò R (2016). NGF controls APP cleavage by downregulating APP phosphorylation at Thr668: relevance for Alzheimer’s disease. Aging Cell.

[82] Canu N, Pagano I, La Rosa LR, Pellegrino M, Ciotti MT, Mercanti D, Moretti F, Sposato V, Triaca V, Petrella C (2017). Association of TrkA and APP Is Promoted by NGF and Reduced by Cell Death-Promoting Agents. Front Mol Neurosci.

[83] Rogaeva E, Meng Y, Lee JH, Gu Y, Kawarai T, Zou F, Katayama T, Baldwin CT, Cheng R, Hasegawa H (2007). The neuronal sortilin-related receptor SORL1 is genetically associated with Alzheimer disease. Nat Genet.

[84] Reitz C, Tosto G, Vardarajan B, Rogaeva E, Ghani M, Rogers RS, Conrad C, Haines JL, Pericak-Vance MA, Fallin MD (2013). Independent and epistatic effects of variants in VPS10-d receptors on Alzheimer disease risk and processing of the amyloid precursor protein (APP). Transl Psychiatry.

[85] Thonberg H, Chiang HH, Lilius L, Forsell C, Lindstrom AK, Johansson C, Bjorkstrom J, Thordardottir S, Sleegers K, Van Broeckhoven C (2017). Identification and description of three families with familial Alzheimer disease that segregate variants in the SORL1 gene. Acta Neuropathol Commun.

[86] Verheijen J, Van den Bossche T, van der Zee J, Engelborghs S, Sanchez-Valle R, Llado A, Graff C, Thonberg H, Pastor P, Ortega-Cubero S (2016). A comprehensive study of the genetic impact of rare variants in SORL1 in European early-onset Alzheimer’s disease. Acta Neuropathol.

[87] Pottier C, Hannequin D, Coutant S, Rovelet-Lecrux A, Wallon D, Rousseau S, Legallic S, Paquet C, Bombois S, Pariente J (2012). High frequency of potentially pathogenic SORL1 mutations in autosomal dominant early-onset Alzheimer disease. Mol Psychiatry.

[88] Alemany S, Ribases M, Vilor-Tejedor N, Bustamante M, Sanchez-Mora C, Bosch R, Richarte V, Cormand B, Casas M, Ramos-Quiroga JA, Sunyer J (2015). New suggestive genetic loci and biological pathways for attention function in adult attention-deficit/hyperactivity disorder. Am J Med Genet B Neuropsychiatr Genet.

[89] Lane RF, St George-Hyslop P, Hempstead BL, Small SA, Strittmatter SM, Gandy S (2012). Vps10 family proteins and the retromer complex in aging-related neurodegeneration and diabetes. J Neurosci.

[90] Lee PH, Anttila V, Won H, Feng Y-CA, Rosenthal J, Zhu Z, Tucker-Drob EM, Nivard MG, Grotzinger AD, Posthuma D (2019). Genomic Relationships, Novel Loci, and Pleiotropic Mechanisms across Eight Psychiatric Disorders. Cell.

[91] Nyborg AC, Ladd TB, Zwizinski CW, Lah JJ, Golde TE (2006). Sortilin, SorCS1b, and SorLA Vps10p sorting receptors, are novel gamma-secretase substrates. Mol Neurodegener.

[92] Lane RF, Raines SM, Steele JW, Ehrlich ME, Lah JA, Small SA, Tanzi RE, Attie AD, Gandy S (2010). Diabetes-associated SorCS1 regulates Alzheimer’s amyloid-beta metabolism: evidence for involvement of SorL1 and the retromer complex. J Neurosci.

[93] Carlo AS, Gustafsen C, Mastrobuoni G, Nielsen MS, Burgert T, Hartl D, Rohe M, Nykjaer A, Herz J, Heeren J (2013). The pro-neurotrophin receptor sortilin is a major neuronal apolipoprotein E receptor for catabolism of amyloid-beta peptide in the brain. J Neurosci.

[94] Philtjens S, Van Mossevelde S, van der Zee J, Wauters E, Dillen L, Vandenbulcke M, Vandenberghe R, Ivanoiu A, Sieben A, Willems C (2018). Rare nonsynonymous variants in SORT1 are associated with increased risk for frontotemporal dementia. Neurobiol Aging.

[95] Andersen OM, Reiche J, Schmidt V, Gotthardt M, Spoelgen R, Behlke J, von Arnim CA, Breiderhoff T, Jansen P, Wu X (2005). Neuronal sorting protein-related receptor sorLA/LR11 regulates processing of the amyloid precursor protein. Proc Natl Acad Sci USA.

[96] Huang TY, Zhao Y, Jiang LL, Li X, Liu Y, Sun Y, Pina-Crespo JC, Zhu B, Masliah E, Willnow TE (2017). SORLA attenuates EphA4 signaling and amyloid beta-induced neurodegeneration. J Exp Med.

[97] Gustafsen C, Glerup S, Pallesen LT, Olsen D, Andersen OM, Nykjaer A, Madsen P, Petersen CM (2013). Sortilin and SorLA display distinct roles in processing and trafficking of amyloid precursor protein. J Neurosci.

[98] Schmidt V, Baum K, Lao A, Rateitschak K, Schmitz Y, Teichmann A, Wiesner B, Petersen CM, Nykjaer A, Wolf J (2012). Quantitative modelling of amyloidogenic processing and its influence by SORLA in Alzheimer’s disease. EMBO J.

[99] Mehmedbasic A, Christensen SK, Nilsson J, Ruetschi U, Gustafsen C, Poulsen AS, Rasmussen RW, Fjorback AN, Larson G, Andersen OM (2015). SorLA complement-type repeat domains protect the amyloid precursor protein against processing. J Biol Chem.

[100] Andersen OM, Schmidt V, Spoelgen R, Gliemann J, Behlke J, Galatis D, McKinstry WJ, Parker MW, Masters CL, Hyman BT (2006). Molecular dissection of the interaction between amyloid precursor protein and its neuronal trafficking receptor SorLA/LR11. Biochemistry.

[101] Hermey G, Hoffmeister-Ullerich SA, Merz B, Gross D, Kuhl D, Kins S (2019). Amyloidosis causes downregulation of SorLA, SorCS1 and SorCS3 expression in mice. Biol Chem.

[102] Nunes AF, Amaral JD, Lo AC, Fonseca MB, Viana RJ, Callaerts-Vegh Z, D’Hooge R, Rodrigues CM (2012). TUDCA, a bile acid, attenuates amyloid precursor protein processing and amyloid-β deposition in APP/PS1 mice. Mol Neurobiol.

[103] Finan GM, Okada H, Kim TW (2011). BACE1 retrograde trafficking is uniquely regulated by the cytoplasmic domain of sortilin. J Biol Chem.

[104] Ruan CS, Liu J, Yang M, Saadipour K, Zeng YQ, Liao H, Wang YJ, Bobrovskaya L, Zhou XF (2018). Sortilin inhibits amyloid pathology by regulating non-specific degradation of APP. Exp Neurol.

[105] Yang M, Virassamy B, Vijayaraj SL, Lim Y, Saadipour K, Wang YJ, Han YC, Zhong JH, Morales CR, Zhou XF (2013). The intracellular domain of sortilin interacts with amyloid precursor protein and regulates its lysosomal and lipid raft trafficking. PLoS ONE.

[106] Lane RF, Steele JW, Cai D, Ehrlich ME, Attie AD, Gandy S (2013). Protein sorting motifs in the cytoplasmic tail of SorCS1 control generation of Alzheimer’s amyloid-beta peptide. J Neurosci.

[107] Hermey G, Schmidt N, Bluhm B, Mensching D, Ostermann K, Rupp C, Kuhl D, Kins S (2015). SorCS1 variants and amyloid precursor protein (APP) are co-transported in neurons but only SorCS1c modulates anterograde APP transport. J Neurochem.

[108] Reitz C, Tokuhiro S, Clark LN, Conrad C, Vonsattel JP, Hazrati LN, Palotas A, Lantigua R, Medrano M, Z Jiménez-Velázquez I (2011). SORCS1 alters amyloid precursor protein processing and variants may increase Alzheimer’s disease risk. Ann Neurol.

[109] Kitago Y, Nagae M, Nakata Z, Yagi-Utsumi M, Takagi-Niidome S, Mihara E, Nogi T, Kato K, Takagi J (2015). Structural basis for amyloidogenic peptide recognition by sorLA. Nat Struct Mol Biol.

[110] Yajima R, Tokutake T, Koyama A, Kasuga K, Tezuka T, Nishizawa M, Ikeuchi T (2015). ApoE-isoform-dependent cellular uptake of amyloid-beta is mediated by lipoprotein receptor LR11/SorLA. Biochem Biophys Res Commun.

[111] Caglayan S, Takagi-Niidome S, Liao F, Carlo AS, Schmidt V, Burgert T, Kitago Y, Fuchtbauer EM, Fuchtbauer A, Holtzman DM (2014). Lysosomal sorting of amyloid-beta by the SORLA receptor is impaired by a familial Alzheimer’s disease mutation. Sci Transl Med.

[112] Takamura A, Sato Y, Watabe D, Okamoto Y, Nakata T, Kawarabayashi T, Oddo S, Laferla FM, Shoji M, Matsubara E (2012). Sortilin is required for toxic action of Abeta oligomers (AbetaOs): extracellular AbetaOs trigger apoptosis, and intraneuronal AbetaOs impair degradation pathways. Life Sci.

[113] Saadipour K, Yang M, Lim Y, Georgiou K, Sun Y, Keating D, Liu J, Wang YR, Gai WP, Zhong JH (2013). Amyloid beta(1)(-)(4)(2) (Abeta(4)(2)) up-regulates the expression of sortilin via the p75(NTR)/RhoA signaling pathway. J Neurochem.

[114] Spoelgen R, von Arnim CA, Thomas AV, Peltan ID, Koker M, Deng A, Irizarry MC, Andersen OM, Willnow TE, Hyman BT (2006). Interaction of the cytosolic domains of sorLA/LR11 with the amyloid precursor protein (APP) and beta-secretase beta-site APP-cleaving enzyme. J Neurosci.

[115] Hermey G, Sjogaard SS, Petersen CM, Nykjaer A, Gliemann J (2006). Tumour necrosis factor alpha-converting enzyme mediates ectodomain shedding of Vps10p-domain receptor family members. Biochem J.

[116] Yamazaki H, Bujo H, Kusunoki J, Seimiya K, Kanaki T, Morisaki N, Schneider WJ, Saito Y (1996). Elements of neural adhesion molecules and a yeast vacuolar protein sorting receptor are present in a novel mammalian low density lipoprotein receptor family member. J Biol Chem.

[117] Evans SF, Irmady K, Ostrow K, Kim T, Nykjaer A, Saftig P, Blobel C, Hempstead BL (2011). Neuronal brain-derived neurotrophic factor is synthesized in excess, with levels regulated by sortilin-mediated trafficking and lysosomal degradation. J Biol Chem.

[118] Navarro V, Vincent JP, Mazella J (2002). Shedding of the luminal domain of the neurotensin receptor-3/sortilin in the HT29 cell line. Biochem Biophys Res Commun.

[119] Hermans-Borgmeyer I, Hampe W, Schinke B, Methner A, Nykjaer A, Susens U, Fenger U, Herbarth B, Schaller HC (1998). Unique expression pattern of a novel mosaic receptor in the developing cerebral cortex. Mech Dev.

[120] Hermans-Borgmeyer I, Hermey G, Nykjaer A, Schaller C (1999). Expression of the 100-kDa neurotensin receptor sortilin during mouse embryonal development. Brain Res Mol Brain Res.

[121] Hermey G, Plath N, Hubner CA, Kuhl D, Schaller HC, Hermans-Borgmeyer I (2004). The three sorCS genes are differentially expressed and regulated by synaptic activity. J Neurochem.

[122] Oetjen S, Mahlke C, Hermans-Borgmeyer I, Hermey G (2014). Spatiotemporal Expression Analysis of the Growth Factor Receptor SorCS3. J Comp Neurol.

[123] Rezgaoui M, Hermey G, Riedel IB, Hampe W, Schaller HC, Hermans-Borgmeyer I (2001). Identification of SorCS2, a novel member of the VPS10 domain containing receptor family, prominently expressed in the developing mouse brain. Mech Dev.

[124] Maag JL, Panja D, Sporild I, Patil S, Kaczorowski DC, Bramham CR, Dinger ME, Wibrand K (2015). Dynamic expression of long noncoding RNAs and repeat elements in synaptic plasticity. Front Neurosci.

[125] Hermey G, Mahlke C, Gutzmann JJ, Schreiber J, Blüthgen N, Kuhl D (2013). Genome-Wide Profiling of the Activity-Dependent Hippocampal Transcriptome. PLoS ONE.

[126] Rao-Ruiz P, Couey JJ, Marcelo IM, Bouwkamp CG, Slump DE, Matos MR, van der Loo RJ, Martins GJ, van den Hout M, van IJcken WF (2019). Engram-specific transcriptome profiling of contextual memory consolidation. Nat Commun.

[127] Visanji NP, Kamali Sarvestani I, Creed MC, Shams Shoaei Z, Nobrega JN, Hamani C, Hazrati LN (2015). Deep brain stimulation of the subthalamic nucleus preferentially alters the translational profile of striatopallidal neurons in an animal model of Parkinson’s disease. Front Cell Neurosci.

[128] Talbot H, Saada S, Naves T, Gallet PF, Fauchais AL, Jauberteau MO (2018). Regulatory Roles of Sortilin and SorLA in Immune-Related Processes. Front Pharmacol.

[129] Hermey G, Riedel IB, Hampe W, Schaller HC, Hermans-Borgmeyer I (1999). Identification and characterization of SorCS, a third member of a novel receptor family. Biochem Biophys Res Commun.

[130] Hermey G, Schaller HC (2000). Alternative splicing of murine SorCS leads to two forms of the receptor that differ completely in their cytoplasmic tails. Biochem Biophys Acta.

[131] Hampe W, Rezgaoui M, Hermans-Borgmeyer I, Schaller HC (2001). The genes for the human VPS10 domain-containing receptors are large and contain many small exons. Hum Genet.

[132] Glerup S, Olsen D, Vaegter CB, Gustafsen C, Sjoegaard SS, Hermey G, Kjolby M, Molgaard S, Ulrichsen M, Boggild S (2014). SorCS2 Regulates Dopaminergic Wiring and Is Processed into an Apoptotic Two-Chain Receptor in Peripheral Glia. Neuron.

[133] Savas JN, Ribeiro LF, Wierda KD, Wright R, DeNardo-Wilke LA, Rice HC, Chamma I, Wang YZ, Zemla R, Lavallee-Adam M (2015). The Sorting Receptor SorCS1 Regulates Trafficking of Neurexin and AMPA Receptors. Neuron.

[134] Teng HK, Teng KK, Lee R, Wright S, Tevar S, Almeida RD, Kermani P, Torkin R, Chen ZY, Lee FS (2005). ProBDNF induces neuronal apoptosis via activation of a receptor complex of p75NTR and sortilin. J Neurosci.

[135] Leloup N, Chataigner LMP, Janssen BJC (2018). Structural insights into SorCS2-Nerve Growth Factor complex formation. Nat Commun.

[136] Westergaard UB, Kirkegaard K, Sorensen ES, Jacobsen C, Nielsen MS, Petersen CM, Madsen P (2005). SorCS3 does not require propeptide cleavage to bind nerve growth factor. FEBS Lett.

[137] Rohe M, Synowitz M, Glass R, Paul SM, Nykjaer A, Willnow TE (2009). Brain-derived neurotrophic factor reduces amyloidogenic processing through control of SORLA gene expression. J Neurosci.

[138] Chen ZY, Ieraci A, Teng H, Dall H, Meng CX, Herrera DG, Nykjaer A, Hempstead BL, Lee FS (2005). Sortilin controls intracellular sorting of brain-derived neurotrophic factor to the regulated secretory pathway. J Neurosci.

[139] Anastasia A, Deinhardt K, Chao MV, Will NE, Irmady K, Lee FS, Hempstead BL, Bracken C (2013). Val66Met polymorphism of BDNF alters prodomain structure to induce neuronal growth cone retraction. Nat Commun.

[140] Tauris J, Gustafsen C, Christensen EI, Jansen P, Nykjaer A, Nyengaard JR, Teng KK, Schwarz E, Ovesen T, Madsen P, Petersen CM (2011). Proneurotrophin-3 may induce Sortilin-dependent death in inner ear neurons. Eur J Neurosci.

[141] Yano H, Torkin R, Martin LA, Chao MV, Teng KK (2009). Proneurotrophin-3 is a neuronal apoptotic ligand: evidence for retrograde-directed cell killing. J Neurosci.

[142] Vaegter CB, Jansen P, Fjorback AW, Glerup S, Skeldal S, Kjolby M, Richner M, Erdmann B, Nyengaard JR, Tessarollo L (2011). Sortilin associates with Trk receptors to enhance anterograde transport and neurotrophin signaling. Nat Neurosci.

[143] Rohe M, Hartl D, Fjorback AN, Klose J, Willnow TE (2013). SORLA-mediated trafficking of TrkB enhances the response of neurons to BDNF. PLoS ONE.

[144] Subkhangulova A, Malik AR, Hermey G, Popp O, Dittmar G, Rathjen T, Poy MN, Stumpf A, Beed PS, Schmitz D (2018). SORCS1 and SORCS3 control energy balance and orexigenic peptide production. EMBO reports.

[145] Jacobsen L, Madsen P, Jacobsen C, Nielsen MS, Gliemann J, Petersen CM (2001). Activation and functional characterization of the mosaic receptor SorLA/LR11. J Biol Chem.

[146] Munck Petersen C, Nielsen MS, Jacobsen C, Tauris J, Jacobsen L, Gliemann J, Moestrup SK, Madsen P (1999). Propeptide cleavage conditions sortilin/neurotensin receptor-3 for ligand binding. EMBO J.

[147] Nielsen MS, Jacobsen C, Olivecrona G, Gliemann J, Petersen CM (1999). Sortilin/neurotensin receptor-3 binds and mediates degradation of lipoprotein lipase. J Biol Chem.

[148] Hermey G, Keat SJ, Madsen P, Jacobsen C, Petersen CM, Gliemann J (2003). Characterization of sorCS1, an alternatively spliced receptor with completely different cytoplasmic domains that mediate different trafficking in cells. J Biol Chem.

[149] Whittle AJ, Jiang M, Peirce V, Relat J, Virtue S, Ebinuma H, Fukamachi I, Yamaguchi T, Takahashi M, Murano T (2015). Soluble LR11/SorLA represses thermogenesis in adipose tissue and correlates with BMI in humans. Nat Commun.

[150] Stupack J, Xiong XP, Jiang LL, Zhang T, Zhou L, Campos A, Ranscht B, Mobley W, Pasquale EB, Xu H, Huang TY (2020). Soluble SORLA Enhances Neurite Outgrowth and Regeneration through Activation of the EGF Receptor/ERK Signaling Axis. J Neurosci.

[151] Ogawa K, Ueno T, Iwasaki T, Kujiraoka T, Ishihara M, Kunimoto S, Takayama T, Kanai T, Hirayama A, Hattori H (2016). Soluble sortilin is released by activated platelets and its circulating levels are associated with cardiovascular risk factors. Atherosclerosis.

[152] Januliene D, Manavalan A, Ovesen PL, Pedersen KM, Thirup S, Nykjaer A, Moeller A (2017). Hidden Twins: SorCS Neuroreceptors Form Stable Dimers. J Mol Biol.

[153] Malik AR, Willnow TE (2020). VPS10P Domain Receptors: Sorting Out Brain Health and Disease. Trends Neurosci.

[154] Yabe-Wada T, Matsuba S, Unno M, Onai N (2018). Crystal structure of the ligand-free form of the Vps10 ectodomain of dimerized Sortilin at acidic pH. FEBS Lett.

[155] Leloup N, Lossl P, Meijer DH, Brennich M, Heck AJR, Thies-Weesie DME, Janssen BJC (2017). Low pH-induced conformational change and dimerization of sortilin triggers endocytosed ligand release. Nat Commun.

[156] Dong F, Wu C, Jiang W, Zhai M, Li H, Zhai L, Zhang X (2022). Cryo-EM structure studies of the human VPS10 domain-containing receptor SorCS3. Biochem Biophys Res Commun.

[157] Zhang X, Wu C, Song Z, Sun D, Zhai L, Liu C (2022). Cryo-EM structures reveal distinct apo conformations of sortilin-related receptor SORLA. Biochem Biophys Res Commun.

[158] Holstege H, van der Lee SJ, Hulsman M, Wong TH, van Rooij JG, Weiss M, Louwersheimer E, Wolters FJ, Amin N, Uitterlinden AG (2017). Characterization of pathogenic SORL1 genetic variants for association with Alzheimer’s disease: a clinical interpretation strategy. Eur J Human Genet.

[159] Raghavan NS, Brickman AM, Andrews H, Manly JJ, Schupf N, Lantigua R, Wolock CJ, Kamalakaran S, Petrovski S, Tosto G (2018). Whole-exome sequencing in 20,197 persons for rare variants in Alzheimer’s disease. Ann Clin Transl Neurol.

[160] Louwersheimer E, Cohn-Hokke PE, Pijnenburg YA, Weiss MM, Sistermans EA, Rozemuller AJ, Hulsman M, van Swieten JC, van Duijn CM, Barkhof F (2017). Rare Genetic Variant in SORL1 May Increase Penetrance of Alzheimer’s Disease in a Family with Several Generations of APOE-varepsilon4 Homozygosity. J Alzheimers Dis.

[161] Miyashita A, Koike A, Jun G, Wang LS, Takahashi S, Matsubara E, Kawarabayashi T, Shoji M, Tomita N, Arai H (2013). SORL1 is genetically associated with late-onset Alzheimer’s disease in Japanese, Koreans and Caucasians. PloS one.

[162] Lambert JC, Ibrahim-Verbaas CA, Harold D, Naj AC, Sims R, Bellenguez C, DeStafano AL, Bis JC, Beecham GW, Grenier-Boley B (2013). Meta-analysis of 74,046 individuals identifies 11 new susceptibility loci for Alzheimer’s disease. Nat Genet.

[163] Bettens K, Brouwers N, Engelborghs S, De Deyn PP, Van Broeckhoven C, Sleegers K (2008). SORL1 is genetically associated with increased risk for late-onset Alzheimer disease in the Belgian population. Hum Mutat.

[164] Lee JH, Cheng R, Schupf N, Manly J, Lantigua R, Stern Y, Rogaeva E, Wakutani Y, Farrer L, St George-Hyslop P, Mayeux R (2007). The association between genetic variants in SORL1 and Alzheimer disease in an urban, multiethnic, community-based cohort. Arch Neurol.

[165] Fernández MV, Black K, Carrell D, Saef B, Budde J, Deming Y, Howells B, Del-Aguila JL, Ma S, Bi C (2016). SORL1 variants across Alzheimer’s disease European American cohorts. Eur J Human Genet.

[166] Gómez-Tortosa E, Ruggiero M, Sainz MJ, Villarejo-Galende A, Prieto-Jurczynska C, Venegas Pérez B, Ordás C, Agüero P, Guerrero-López R, Pérez-Pérez J (2018). SORL1 Variants in Familial Alzheimer’s Disease. J Alzheimers Dis.

[167] Korpioja A, Krüger J, Koivuluoma S, Pylkäs K, Moilanen V, Helisalmi S, Hiltunen M, Remes AM. Novel Rare SORL1 Variants in Early-Onset Dementia. Journal of Alzheimer’s disease : JAD. 2021;82:761–70. 10.3233/JAD-210207 34092641

[168] Holstege H, Hulsman M, Charbonnier C, Grenier-Boley B, Quenez O, Ahmad S, Amin N, van Rooij JGJ, Grozeva D, Norsworthy P, et al. Exome sequencing identifies three novel AD-associated genes. Alzheimer's & Dementia. 2020;16:e041592. 10.1002/alz.041592.

[169] Nicolas G, Charbonnier C, Wallon D, Quenez O, Bellenguez C, Grenier-Boley B, Rousseau S, Richard AC, Rovelet-Lecrux A, Le Guennec K, et al. SORL1 rare variants: a major risk factor for familial early-onset Alzheimer's disease. Mol Psychiatry. 2016;21:831-836. 10.1038/mp.2015.121. 10.1038/mp.2015.121 26303663

[170] Alvarez-Mora MI, Blanco-Palmero VA, Quesada-Espinosa JF, Arteche-Lopez AR, Llamas-Velasco S, Palma Milla C, Lezana Rosales JM, Gomez-Manjon I, Hernandez-Lain A, Jimenez Almonacid J (2022). Heterozygous and Homozygous Variants in SORL1 Gene in Alzheimer’s Disease Patients: Clinical, Neuroimaging and Neuropathological Findings. Int J Mol Sci.

[171] El Bitar F, Qadi N, Al Rajeh S, Majrashi A, Abdulaziz S, Majrashi N, Al Inizi M, Taher A, Al Tassan N (2019). Genetic Study of Alzheimer’s Disease in Saudi Population. J Alzheimers Dis.

[172] Jacobsen L, Madsen P, Moestrup SK, Lund AH, Tommerup N, Nykjaer A, Sottrup-Jensen L, Gliemann J, Petersen CM (1996). Molecular characterization of a novel human hybrid-type receptor that binds the alpha2-macroglobulin receptor-associated protein. J Biol Chem.

[173] Hampe W, Frank RW, Schulze C, Dehning I, Schaller HC (1996). Photoaffinity labeling of the head-activator receptor from hydra. Eur J Biochem.

[174] Glerup S, Lume M, Olsen D, Nyengaard JR, Vaegter CB, Gustafsen C, Christensen EI, Kjolby M, Hay-Schmidt A, Bender D (2013). SorLA controls neurotrophic activity by sorting of GDNF and its receptors GFRalpha1 and RET. Cell Rep.

[175] Nielsen MS, Gustafsen C, Madsen P, Nyengaard JR, Hermey G, Bakke O, Mari M, Schu P, Pohlmann R, Dennes A, Petersen CM (2007). Sorting by the cytoplasmic domain of the amyloid precursor protein binding receptor SorLA. Mol Cell Biol.

[176] Huang TY, Zhao Y, Li X, Wang X, Tseng IC, Thompson R, Tu S, Willnow TE, Zhang YW, Xu H (2016). SNX27 and SORLA Interact to Reduce Amyloidogenic Subcellular Distribution and Processing of Amyloid Precursor Protein. J Neurosci.

[177] Fjorback AW, Seaman M, Gustafsen C, Mehmedbasic A, Gokool S, Wu C, Militz D, Schmidt V, Madsen P, Nyengaard JR (2012). Retromer binds the FANSHY sorting motif in SorLA to regulate amyloid precursor protein sorting and processing. J Neurosci.

[178] Schmidt V, Sporbert A, Rohe M, Reimer T, Rehm A, Andersen OM, Willnow TE (2007). SorLA/LR11 regulates processing of amyloid precursor protein via interaction with adaptors GGA and PACS-1. J Biol Chem.

[179] Jacobsen L, Madsen P, Nielsen MS, Geraerts WP, Gliemann J, Smit AB, Petersen CM (2002). The sorLA cytoplasmic domain interacts with GGA1 and -2 and defines minimum requirements for GGA binding. FEBS Lett.

[180] Farías GG, Cuitino L, Guo X, Ren X, Jarnik M, Mattera R, Bonifacino JS (2012). Signal-mediated, AP-1/clathrin-dependent sorting of transmembrane receptors to the somatodendritic domain of hippocampal neurons. Neuron.

[181] Klinger SC, Hojland A, Jain S, Kjolby M, Madsen P, Svendsen AD, Olivecrona G, Bonifacino JS, Nielsen MS (2016). Polarized trafficking of the sorting receptor SorLA in neurons and MDCK cells. Febs j.

[182] Madsen P, Isaksen TJ, Siupka P, Tóth AE, Nyegaard M, Gustafsen C, Nielsen MS (2019). HSPA12A targets the cytoplasmic domain and affects the trafficking of the Amyloid Precursor Protein receptor SorLA. Sci Rep.

[183] Binkle L, Klein M, Borgmeyer U, Kuhl D, Hermey G (2022). The adaptor protein PICK1 targets the sorting receptor SorLA. Mol Brain.

[184] Monti G, Kjolby M, Jensen AMG, Allen M, Reiche J, Møller PL, Comaposada-Baró R, Zolkowski BE, Vieira C, Jørgensen MM (2021). Expression of an alternatively spliced variant of SORL1 in neuronal dendrites is decreased in patients with Alzheimer’s disease. Acta Neuropathol Commun.

[185] Scherzer CR, Offe K, Gearing M, Rees HD, Fang G, Heilman CJ, Schaller C, Bujo H, Levey AI, Lah JJ (2004). Loss of apolipoprotein E receptor LR11 in Alzheimer disease. Arch Neurol.

[186] Dodson SE, Gearing M, Lippa CF, Montine TJ, Levey AI, Lah JJ (2006). LR11/SorLA expression is reduced in sporadic Alzheimer disease but not in familial Alzheimer disease. J Neuropathol Exp Neurol.

[187] Dodson SE, Andersen OM, Karmali V, Fritz JJ, Cheng D, Peng J, Levey AI, Willnow TE, Lah JJ (2008). Loss of LR11/SORLA enhances early pathology in a mouse model of amyloidosis: evidence for a proximal role in Alzheimer’s disease. J Neurosci.

[188] Rohe M, Carlo AS, Breyhan H, Sporbert A, Militz D, Schmidt V, Wozny C, Harmeier A, Erdmann B, Bales KR (2008). Sortilin-related receptor with A-type repeats (SORLA) affects the amyloid precursor protein-dependent stimulation of ERK signaling and adult neurogenesis. J Biol Chem.

[189] Ma QL, Galasko DR, Ringman JM, Vinters HV, Edland SD, Pomakian J, Ubeda OJ, Rosario ER, Teter B, Frautschy SA, Cole GM (2009). Reduction of SorLA/LR11, a sorting protein limiting beta-amyloid production, in Alzheimer disease cerebrospinal fluid. Arch Neurol.

[190] Ikeuchi T, Hirayama S, Miida T, Fukamachi I, Tokutake T, Ebinuma H, Takubo K, Kaneko H, Kasuga K, Kakita A (2010). Increased Levels of Soluble LR11 in Cerebrospinal Fluid of Patients with Alzheimer Disease. Dement Geriatr Cogn Disord.

[191] Graebert KS, Popp GM, Kehle T, Herzog V (1995). Regulated O-glycosylation of the Alzheimer beta-A4 amyloid precursor protein in thyrocytes. Eur J Cell Biol.

[192] Georgopoulou N, McLaughlin M, McFarlane I, Breen KC. The role of post-translational modification in beta-amyloid precursor protein processing. Biochem Soc Symp. 2001;(67):23-36. 10.1042/bss0670023. 10.1042/bss0670023 11447837

[193] Blechingberg J, Poulsen ASA, Kjolby M, Monti G, Allen M, Ivarsen AK, Lincoln SJ, Thotakura G, Vaegter CB, Ertekin-Taner N (2018). An alternative transcript of the Alzheimer’s disease risk gene SORL1 encodes a truncated receptor. Neurobiol Aging.

[194] Tsolakidou A, Alexopoulos P, Guo LH, Grimmer T, Westerteicher C, Kratzer M, Jiang M, Bujo H, Roselli F, Leante MR (2013). beta-Site amyloid precursor protein-cleaving enzyme 1 activity is related to cerebrospinal fluid concentrations of sortilin-related receptor with A-type repeats, soluble amyloid precursor protein, and tau. Alzheimers Dement.

[195] Simoes S, Guo J, Buitrago L, Qureshi YH, Feng X, Kothiya M, Cortes E, Patel V, Kannan S, Kim YH (2021). Alzheimer’s vulnerable brain region relies on a distinct retromer core dedicated to endosomal recycling. Cell Rep.

[196] Small SA, Petsko GA (2020). Endosomal recycling reconciles the Alzheimer’s disease paradox. Sci Transl Med.

[197] Small SA, Kent K, Pierce A, Leung C, Kang MS, Okada H, Honig L, Vonsattel JP, Kim TW (2005). Model-guided microarray implicates the retromer complex in Alzheimer’s disease. Ann Neurol.

[198] Vagnozzi AN, Pratico D (2019). Endosomal sorting and trafficking, the retromer complex and neurodegeneration. Mol Psychiatry.

[199] Chandra M, Kendall AK, Jackson LP (2021). Toward Understanding the Molecular Role of SNX27/Retromer in Human Health and Disease. Front Cell Dev Biol.

[200] Young JE, Boulanger-Weill J, Williams DA, Woodruff G, Buen F, Revilla AC, Herrera C, Israel MA, Yuan SH, Edland SD, Goldstein LS (2015). Elucidating molecular phenotypes caused by the SORL1 Alzheimer’s disease genetic risk factor using human induced pluripotent stem cells. Cell Stem Cell.

[201] Murai KK, Pasquale EB (2004). Eph receptors, ephrins, and synaptic function. Neuroscientist.

[202] Egea J, Nissen UV, Dufour A, Sahin M, Greer P, Kullander K, Mrsic-Flogel TD, Greenberg ME, Kiehn O, Vanderhaeghen P, Klein R (2005). Regulation of EphA 4 kinase activity is required for a subset of axon guidance decisions suggesting a key role for receptor clustering in Eph function. Neuron.

[203] Rosenberger AF, Rozemuller AJ, van der Flier WM, Scheltens P, van der Vies SM, Hoozemans JJ (2014). Altered distribution of the EphA4 kinase in hippocampal brain tissue of patients with Alzheimer’s disease correlates with pathology. Acta Neuropathol Commun.

[204] Fu AK, Hung KW, Huang H, Gu S, Shen Y, Cheng EY, Ip FC, Huang X, Fu WY, Ip NY (2014). Blockade of EphA4 signaling ameliorates hippocampal synaptic dysfunctions in mouse models of Alzheimer’s disease. Proc Natl Acad Sci USA.

[205] Vargas LM, Leal N, Estrada LD, Gonzalez A, Serrano F, Araya K, Gysling K, Inestrosa NC, Pasquale EB, Alvarez AR (2014). EphA4 activation of c-Abl mediates synaptic loss and LTP blockade caused by amyloid-beta oligomers. PLoS ONE.

[206] Simon AM, de Maturana RL, Ricobaraza A, Escribano L, Schiapparelli L, Cuadrado-Tejedor M, Perez-Mediavilla A, Avila J, Del Rio J, Frechilla D (2009). Early changes in hippocampal Eph receptors precede the onset of memory decline in mouse models of Alzheimer’s disease. J Alzheimers Dis.

[207] Vardarajan BN, Ghani M, Kahn A, Sheikh S, Sato C, Barral S, Lee JH, Cheng R, Reitz C, Lantigua R (2015). Rare coding mutations identified by sequencing of Alzheimer disease genome-wide association studies loci. Ann Neurol.

[208] Schmidt V, Schulz N, Yan X, Schürmann A, Kempa S, Kern M, Blüher M, Poy MN, Olivecrona G, Willnow TE (2016). SORLA facilitates insulin receptor signaling in adipocytes and exacerbates obesity. J Clin Investig.

[209] Takahashi M, Bujo H, Jiang M, Noike H, Saito Y, Shirai K (2010). Enhanced circulating soluble LR11 in patients with coronary organic stenosis. Atherosclerosis.

[210] Jin W, Jiang M, Han X, Han X, Murano T, Hiruta N, Ebinuma H, Piao L, Schneider WJ, Bujo H (2016). Circulating soluble form of LR11, a regulator of smooth muscle cell migration, is a novel marker for intima-media thickness of carotid arteries in type 2 diabetes. Clin Chim Acta.

[211] Ma QL, Teter B, Ubeda OJ, Morihara T, Dhoot D, Nyby MD, Tuck ML, Frautschy SA, Cole GM (2007). Omega-3 fatty acid docosahexaenoic acid increases SorLA/LR11, a sorting protein with reduced expression in sporadic Alzheimer’s disease (AD): relevance to AD prevention. J Neurosci.

[212] Ghanim H, Monte SV, Sia CL, Abuaysheh S, Green K, Caruana JA, Dandona P (2012). Reduction in inflammation and the expression of amyloid precursor protein and other proteins related to Alzheimer’s disease following gastric bypass surgery. J Clin Endocrinol Metab.

[213] Berk KA, Vongpromek R, Jiang M, Schneider WJ, Timman R, Verhoeven AJ, Bujo H, Sijbrands EJ, Mulder MT (2016). Levels of the soluble LDL receptor-relative LR11 decrease in overweight individuals with type 2 diabetes upon diet-induced weight loss. Atherosclerosis.

[214] Cifre M, Palou A, Oliver P (2018). Cognitive impairment in metabolically-obese, normal-weight rats: identification of early biomarkers in peripheral blood mononuclear cells. Mol Neurodegener.

[215] Zhang S, Zhao M, Wang F, Liu J, Zheng H, Lei P (2020). Relationship between normal weight obesity and mild cognitive impairment is reflected in cognitive-related genes in human peripheral blood mononuclear cells. Psychogeriatrics.

[216] Schmidt V, Horváth C, Dong H, Blüher M, Qvist P, Wolfrum C, Willnow TE (2021). SORLA is required for insulin-induced expansion of the adipocyte precursor pool in visceral fat. J Cell Biol.

[217] Andersson CH, Hansson O, Minthon L, Andreasen N, Blennow K, Zetterberg H, Skoog I, Wallin A, Nilsson S, Kettunen P (2016). A Genetic Variant of the Sortilin 1 Gene is Associated with Reduced Risk of Alzheimer’s Disease. J Alzheimers Dis.

[218] Hu X, Hu ZL, Li Z, Ruan CS, Qiu WY, Pan A, Li CQ, Cai Y, Shen L, Chu Y (2017). Sortilin Fragments Deposit at Senile Plaques in Human Cerebrum. Front Neuroanat.

[219] Mufson EJ, Wuu J, Counts SE, Nykjaer A (2010). Preservation of cortical sortilin protein levels in MCI and Alzheimer’s disease. Neurosci Lett.

[220] Zhou FQ, Jiang J, Griffith CM, Patrylo PR, Cai H, Chu Y, Yan XX (2018). Lack of human-like extracellular sortilin neuropathology in transgenic Alzheimer’s disease model mice and macaques. Alzheimers Res Ther.

[221] Petersen CM, Nielsen MS, Nykjaer A, Jacobsen L, Tommerup N, Rasmussen HH, Roigaard H, Gliemann J, Madsen P, Moestrup SK (1997). Molecular identification of a novel candidate sorting receptor purified from human brain by receptor-associated protein affinity chromatography. J Biol Chem.

[222] Sarret P, Krzywkowski P, Segal L, Nielsen MS, Petersen CM, Mazella J, Stroh T, Beaudet A (2003). Distribution of NTS3 receptor/sortilin mRNA and protein in the rat central nervous system. J Comp Neurol.

[223] Johnson NR, Condello C, Guan S, Oehler A, Becker J, Gavidia M, Carlson GA, Giles K, Prusiner SB (2017). Evidence for sortilin modulating regional accumulation of human tau prions in transgenic mice. Proc Natl Acad Sci USA.

[224] Nielsen MS, Madsen P, Christensen EI, Nykjaer A, Gliemann J, Kasper D, Pohlmann R, Petersen CM (2001). The sortilin cytoplasmic tail conveys Golgi-endosome transport and binds the VHS domain of the GGA2 sorting protein. EMBO J.

[225] Baltes J, Larsen JV, Radhakrishnan K, Geumann C, Kratzke M, Petersen CM, Schu P (2014). σ1B adaptin regulates adipogenesis by mediating the sorting of sortilin in adipose tissue. J Cell Sci.

[226] Pallesen LT, Gustafsen C, Cramer JF, Petersen SV, Thirup SS, Madsen P, Petersen CM (2020). PAK Kinases Target Sortilin and Modulate Its Sorting. Mol Cell Biol.

[227] Progida C, Nielsen MS, Koster G, Bucci C, Bakke O (2012). Dynamics of Rab7b-dependent transport of sorting receptors. Traffic.

[228] Mari M, Bujny MV, Zeuschner D, Geerts WJ, Griffith J, Petersen CM, Cullen PJ, Klumperman J, Geuze HJ (2008). SNX1 defines an early endosomal recycling exit for sortilin and mannose 6-phosphate receptors. Traffic.

[229] Seaman MNJ (2021). The Retromer Complex: From Genesis to Revelations. Trends Biochem Sci.

[230] Yang M, Lim Y, Li X, Zhong JH, Zhou XF (2011). Precursor of brain-derived neurotrophic factor (proBDNF) forms a complex with Huntingtin-associated protein-1 (HAP1) and sortilin that modulates proBDNF trafficking, degradation, and processing. J Biol Chem.

[231] Walter J, Fluhrer R, Hartung B, Willem M, Kaether C, Capell A, Lammich S, Multhaup G, Haass C (2001). Phosphorylation regulates intracellular trafficking of beta-secretase. J Biol Chem.

[232] Huse JT, Pijak DS, Leslie GJ, Lee VM, Doms RW (2000). Maturation and endosomal targeting of beta-site amyloid precursor protein-cleaving enzyme. The Alzheimer’s disease beta-secretase. J Biol Chem.

[233] He X, Li F, Chang WP, Tang J (2005). GGA proteins mediate the recycling pathway of memapsin 2 (BACE). J Biol Chem.

[234] Tesco G, Koh YH, Kang EL, Cameron AN, Das S, Sena-Esteves M, Hiltunen M, Yang SH, Zhong Z, Shen Y (2007). Depletion of GGA3 stabilizes BACE and enhances beta-secretase activity. Neuron.

[235] Lomoio S, Willen R, Kim W, Ho KZ, Robinson EK, Prokopenko D, Kennedy ME, Tanzi RE, Tesco G (2020). Gga3 deletion and a GGA3 rare variant associated with late onset Alzheimer’s disease trigger BACE1 accumulation in axonal swellings. Sci Transl Med.

[236] Santosa C, Rasche S, Barakat A, Bellingham SA, Ho M, Tan J, Hill AF, Masters CL, McLean C, Evin G (2011). Decreased expression of GGA3 protein in Alzheimer’s disease frontal cortex and increased co-distribution of BACE with the amyloid precursor protein. Neurobiol Dis.

[237] Canuel M, Lefrancois S, Zeng J, Morales CR (2008). AP-1 and retromer play opposite roles in the trafficking of sortilin between the Golgi apparatus and the lysosomes. Biochem Biophys Res Commun.

[238] Lefrancois S, Zeng J, Hassan AJ, Canuel M, Morales CR (2003). The lysosomal trafficking of sphingolipid activator proteins (SAPs) is mediated by sortilin. EMBO J.

[239] Yang W, Xiang Y, Liao MJ, Wu PF, Yang L, Huang GH, Shi BZ, Yi L, Lv SQ (2021). Presenilin1 inhibits glioblastoma cell invasiveness via promoting Sortilin cleavage. Cell Commun Signal.

[240] Hemming ML, Elias JE, Gygi SP, Selkoe DJ (2009). Identification of beta-secretase (BACE1) substrates using quantitative proteomics. PLoS ONE.

[241] Cramer JF, Gustafsen C, Behrens MA, Oliveira CL, Pedersen JS, Madsen P, Petersen CM, Thirup SS (2010). GGA autoinhibition revisited. Traffic.

[242] Yaar M, Zhai S, Pilch PF, Doyle SM, Eisenhauer PB, Fine RE, Gilchrest BA (1997). Binding of beta-amyloid to the p75 neurotrophin receptor induces apoptosis. A possible mechanism for Alzheimer’s disease. J Clin Invest.

[243] Asaro A, Carlo-Spiewok AS, Malik AR, Rothe M, Schipke CG, Peters O, Heeren J, Willnow TE (2020). Apolipoprotein E4 disrupts the neuroprotective action of sortilin in neuronal lipid metabolism and endocannabinoid signaling. Alzheimers Dement.

[244] Asaro A, Sinha R, Bakun M, Kalnytska O, Carlo-Spiewok AS, Rubel T, Rozeboom A, Dadlez M, Kaminska B, Aronica E (2021). ApoE4 disrupts interaction of sortilin with fatty acid-binding protein 7 essential to promote lipid signaling. Journal of cell science.

[245] Mortensen MB, Kjolby M, Gunnersen S, Larsen JV, Palmfeldt J, Falk E, Nykjaer A, Bentzon JF (2014). Targeting sortilin in immune cells reduces proinflammatory cytokines and atherosclerosis. J Clin Investig.

[246] Scott SA, Mufson EJ, Weingartner JA, Skau KA, Crutcher KA (1995). Nerve growth factor in Alzheimer’s disease: increased levels throughout the brain coupled with declines in nucleus basalis. J Neurosci.

[247] Capsoni S, Tiveron C, Amato G, Vignone D, Cattaneo A (2010). Peripheral neutralization of nerve growth factor induces immunosympathectomy and central neurodegeneration in transgenic mice. J Alzheimers Dis.

[248] Tiveron C, Fasulo L, Capsoni S, Malerba F, Marinelli S, Paoletti F, Piccinin S, Scardigli R, Amato G, Brandi R (2013). ProNGF\NGF imbalance triggers learning and memory deficits, neurodegeneration and spontaneous epileptic-like discharges in transgenic mice. Cell Death Differ.

[249] Capsoni S, Amato G, Vignone D, Criscuolo C, Nykjaer A, Cattaneo A (2013). Dissecting the role of sortilin receptor signaling in neurodegeneration induced by NGF deprivation. Biochem Biophys Res Commun.

[250] Capsoni S, Tiveron C, Vignone D, Amato G, Cattaneo A (2010). Dissecting the involvement of tropomyosin-related kinase A and p75 neurotrophin receptor signaling in NGF deficit-induced neurodegeneration. Proc Natl Acad Sci USA.

[251] Ross CA, Poirier MA (2004). Protein aggregation and neurodegenerative disease. Nat Med.

[252] Castle AR, Gill AC (2017). Physiological Functions of the Cellular Prion Protein. Front Mol Biosci.

[253] Chen RJ, Chang WW, Lin YC, Cheng PL, Chen YR (2013). Alzheimer’s amyloid-beta oligomers rescue cellular prion protein induced tau reduction via the Fyn pathway. ACS Chem Neurosci.

[254] Griffiths HH, Whitehouse IJ, Baybutt H, Brown D, Kellett KA, Jackson CD, Turner AJ, Piccardo P, Manson JC, Hooper NM (2011). Prion protein interacts with BACE1 protein and differentially regulates its activity toward wild type and Swedish mutant amyloid precursor protein. J Biol Chem.

[255] Whitehouse IJ, Miners JS, Glennon EB, Kehoe PG, Love S, Kellett KA, Hooper NM (2013). Prion protein is decreased in Alzheimer’s brain and inversely correlates with BACE1 activity, amyloid-beta levels and Braak stage. PLoS ONE.

[256] Parkin ET, Watt NT, Hussain I, Eckman EA, Eckman CB, Manson JC, Baybutt HN, Turner AJ, Hooper NM (2007). Cellular prion protein regulates beta-secretase cleavage of the Alzheimer’s amyloid precursor protein. Proc Natl Acad Sci USA.

[257] Gimbel DA, Nygaard HB, Coffey EE, Gunther EC, Lauren J, Gimbel ZA, Strittmatter SM (2010). Memory impairment in transgenic Alzheimer mice requires cellular prion protein. J Neurosci.

[258] Lauren J, Gimbel DA, Nygaard HB, Gilbert JW, Strittmatter SM (2009). Cellular prion protein mediates impairment of synaptic plasticity by amyloid-beta oligomers. Nature.

[259] Purro SA, Nicoll AJ, Collinge J (2018). Prion Protein as a Toxic Acceptor of Amyloid-beta Oligomers. Biol Psychiat.

[260] Salazar SV, Gallardo C, Kaufman AC, Herber CS, Haas LT, Robinson S, Manson JC, Lee MK, Strittmatter SM (2017). Conditional Deletion of Prnp Rescues Behavioral and Synaptic Deficits after Disease Onset in Transgenic Alzheimer’s Disease. J Neurosci.

[261] Dohler F, Sepulveda-Falla D, Krasemann S, Altmeppen H, Schluter H, Hildebrand D, Zerr I, Matschke J, Glatzel M (2014). High molecular mass assemblies of amyloid-beta oligomers bind prion protein in patients with Alzheimer’s disease. Brain.

[262] Um JW, Kaufman AC, Kostylev M, Heiss JK, Stagi M, Takahashi H, Kerrisk ME, Vortmeyer A, Wisniewski T, Koleske AJ (2013). Metabotropic glutamate receptor 5 is a coreceptor for Alzheimer aβ oligomer bound to cellular prion protein. Neuron.

[263] Haas LT, Kostylev MA, Strittmatter SM (2014). Therapeutic molecules and endogenous ligands regulate the interaction between brain cellular prion protein (PrPC) and metabotropic glutamate receptor 5 (mGluR5). J Biol Chem.

[264] Panes JD, Saavedra P, Pineda B, Escobar K, Cuevas ME, Moraga-Cid G, Fuentealba J, Rivas CI, Rezaei H, Muñoz-Montesino C (2021). PrP (C) as a Transducer of Physiological and Pathological Signals. Front Mol Neurosci.

[265] Takahashi RH, Yokotsuka M, Tobiume M, Sato Y, Hasegawa H, Nagao T, Gouras GK (2021). Accumulation of cellular prion protein within β-amyloid oligomer plaques in aged human brains. Brain Pathol (Zurich, Switzerland).

[266] Brody AH, Strittmatter SM (2018). Synaptotoxic Signaling by Amyloid Beta Oligomers in Alzheimer’s Disease Through Prion Protein and mGluR5. Adv Pharmacol.

[267] Gunther EC, Smith LM, Kostylev MA, Cox TO, Kaufman AC, Lee S, Folta-Stogniew E, Maynard GD, Um JW, Stagi M (2019). Rescue of Transgenic Alzheimer’s Pathophysiology by Polymeric Cellular Prion Protein Antagonists. Cell Rep.

[268] Uchiyama K, Tomita M, Yano M, Chida J, Hara H, Das NR, Nykjaer A, Sakaguchi S (2017). Prions amplify through degradation of the VPS10P sorting receptor sortilin. PLoS Pathog.

[269] Virgilio E, De Marchi F, Contaldi E, Dianzani U, Cantello R, Mazzini L, Comi C (2022). The Role of Tau beyond Alzheimer’s Disease: A Narrative Review. Biomedicines.

[270] Wang XM, Zeng P, Fang YY, Zhang T, Tian Q (2021). Progranulin in neurodegenerative dementia. J Neurochem.

[271] Carrasquillo MM, Nicholson AM, Finch N, Gibbs JR, Baker M, Rutherford NJ, Hunter TA, DeJesus-Hernandez M, Bisceglio GD, Mackenzie IR (2010). Genome-wide screen identifies rs646776 near sortilin as a regulator of progranulin levels in human plasma. Am J Hum Genet.

[272] Hu F, Padukkavidana T, Vaegter CB, Brady OA, Zheng Y, Mackenzie IR, Feldman HH, Nykjaer A, Strittmatter SM (2010). Sortilin-mediated endocytosis determines levels of the frontotemporal dementia protein, progranulin. Neuron.

[273] Meneses A, Koga S, O’Leary J, Dickson DW, Bu G, Zhao N (2021). TDP-43 Pathology in Alzheimer’s Disease. Mol Neurodegener.

[274] Mohagheghi F, Prudencio M, Stuani C, Cook C, Jansen-West K, Dickson DW, Petrucelli L, Buratti E (2016). TDP-43 functions within a network of hnRNP proteins to inhibit the production of a truncated human SORT1 receptor. Hum Mol Genet.

[275] Prudencio M, Jansen-West KR, Lee WC, Gendron TF, Zhang YJ, Xu YF, Gass J, Stuani C, Stetler C, Rademakers R (2012). Misregulation of human sortilin splicing leads to the generation of a nonfunctional progranulin receptor. Proc Natl Acad Sci USA.

[276] Su X, Chen L, Chen X, Dai C, Wang B (2022). Emerging roles of sortilin in affecting the metabolism of glucose and lipid profiles. Bosn J Basic Med Sci.

[277] Mitok KA, Keller MP, Attie AD (2022). Sorting Through the Extensive and Confusing Roles of Sortilin in Metabolic Disease. J Lipid Res.

[278] Bi L, Chiang JY, Ding WX, Dunn W, Roberts B, Li T (2013). Saturated fatty acids activate ERK signaling to downregulate hepatic sortilin 1 in obese and diabetic mice. J Lipid Res.

[279] Kaddai V, Jager J, Gonzalez T, Najem-Lendom R, Bonnafous S, Tran A, Le Marchand-Brustel Y, Gual P, Tanti JF, Cormont M (2009). Involvement of TNF-alpha in abnormal adipocyte and muscle sortilin expression in obese mice and humans. Diabetologia.

[280] Ai D, Baez JM, Jiang H, Conlon DM, Hernandez-Ono A, Frank-Kamenetsky M, Milstein S, Fitzgerald K, Murphy AJ, Woo CW (2012). Activation of ER stress and mTORC1 suppresses hepatic sortilin-1 levels in obese mice. J Clin Investig.

[281] Chen C, Li J, Matye DJ, Wang Y, Li T (2019). Hepatocyte sortilin 1 knockout and treatment with a sortilin 1 inhibitor reduced plasma cholesterol in Western diet-fed mice. J Lipid Res.

[282] Rabinowich L, Fishman S, Hubel E, Thurm T, Park WJ, Pewzner-Jung Y, Saroha A, Erez N, Halpern Z, Futerman AH, Zvibel I (2015). Sortilin deficiency improves the metabolic phenotype and reduces hepatic steatosis of mice subjected to diet-induced obesity. J Hepatol.

[283] Hagita S, Rogers MA, Pham T, Wen JR, Mlynarchik AK, Aikawa M, Aikawa E (2018). Transcriptional control of intestinal cholesterol absorption, adipose energy expenditure and lipid handling by Sortilin. Sci Rep.

[284] Ariga M, Yoneyama Y, Fukushima T, Ishiuchi Y, Ishii T, Sato H, Hakuno F, Nedachi T, Takahashi SI (2017). Glucose deprivation attenuates sortilin levels in skeletal muscle cells. Endocr J.

[285] Li J, Matye DJ, Li T (2015). Insulin resistance induces posttranslational hepatic sortilin 1 degradation in mice. J Biol Chem.

[286] Lin BZ, Pilch PF, Kandror KV (1997). Sortilin is a major protein component of Glut4-containing vesicles. J Biol Chem.

[287] Morris NJ, Ross SA, Lane WS, Moestrup SK, Petersen CM, Keller SR, Lienhard GE (1998). Sortilin is the major 110-kDa protein in GLUT4 vesicles from adipocytes. J Biol Chem.

[288] Shi J, Kandror KV (2005). Sortilin is essential and sufficient for the formation of Glut4 storage vesicles in 3T3-L1 adipocytes. Dev Cell.

[289] Lui A, Sparks R, Patel R, Patel NA (2021). Identification of Sortilin Alternatively Spliced Variants in Mouse 3T3L1 Adipocytes. Int J Mol Sci.

[290] Ariga M, Nedachi T, Katagiri H, Kanzaki M (2008). Functional role of sortilin in myogenesis and development of insulin-responsive glucose transport system in C2C12 myocytes. J Biol Chem.

[291] Pan X, Zaarur N, Singh M, Morin P, Kandror KV (2017). Sortilin and retromer mediate retrograde transport of Glut4 in 3T3-L1 adipocytes. Mol Biol Cell.

[292] Li J, Matye DJ, Wang Y, Li T (2017). Sortilin 1 knockout alters basal adipose glucose metabolism but not diet-induced obesity in mice. FEBS Lett.

[293] Kathiresan S, Melander O, Guiducci C, Surti A, Burtt NP, Rieder MJ, Cooper GM, Roos C, Voight BF, Havulinna AS (2008). Six new loci associated with blood low-density lipoprotein cholesterol, high-density lipoprotein cholesterol or triglycerides in humans. Nat Genet.

[294] Musunuru K, Strong A, Frank-Kamenetsky M, Lee NE, Ahfeldt T, Sachs KV, Li X, Li H, Kuperwasser N, Ruda VM (2010). From noncoding variant to phenotype via SORT1 at the 1p13 cholesterol locus. Nature.

[295] Strong A, Ding Q, Edmondson AC, Millar JS, Sachs KV, Li X, Kumaravel A, Wang MY, Ai D, Guo L (2012). Hepatic sortilin regulates both apolipoprotein B secretion and LDL catabolism. J Clin Investig.

[296] Strong A, Rader DJ (2012). Sortilin as a regulator of lipoprotein metabolism. Curr Atheroscler Rep.

[297] Kjolby M, Andersen OM, Breiderhoff T, Fjorback AW, Pedersen KM, Madsen P, Jansen P, Heeren J, Willnow TE, Nykjaer A (2010). Sort1, encoded by the cardiovascular risk locus 1p13.3, is a regulator of hepatic lipoprotein export. Cell Metab.

[298] Gustafsen C, Kjolby M, Nyegaard M, Mattheisen M, Lundhede J, Buttenschon H, Mors O, Bentzon JF, Madsen P, Nykjaer A, Glerup S (2014). The hypercholesterolemia-risk gene SORT1 facilitates PCSK9 secretion. Cell Metab.

[299] Nielsen MS, Keat SJ, Hamati JW, Madsen P, Gutzmann JJ, Engelsberg A, Pedersen KM, Gustafsen C, Nykjaer A, Gliemann J (2008). Different motifs regulate trafficking of SorCS1 isoforms. Traffic.

[300] Kurochkina N, Guha U (2013). SH3 domains: modules of protein-protein interactions. Biophys Rev.

[301] Hermey G, Riedel IB, Rezgaoui M, Westergaard UB, Schaller C, Hermans-Borgmeyer I (2001). SorCS1, a member of the novel sorting receptor family, is localized in somata and dendrites of neurons throughout the murine brain. Neurosci Lett.

[302] Ribeiro LF, Verpoort B, Nys J, Vennekens KM, Wierda KD, de Wit J (2019). SorCS1-mediated sorting in dendrites maintains neurexin axonal surface polarization required for synaptic function. PLoS Biol.

[303] Januliene D, Andersen JL, Nielsen JA, Quistgaard EM, Hansen M, Strandbygaard D, Moeller A, Petersen CM, Madsen P, Thirup SS (2017). Acidic Environment Induces Dimerization and Ligand Binding Site Collapse in the Vps10p Domain of Sortilin. Structure (London, England: 1993).

[304] Larsen JV, Hermey G, Sorensen ES, Prabakaran T, Christensen EI, Gliemann J, Madsen P, Petersen CM (2014). Human sorCS1 binds sortilin and hampers its cellular functions. Biochem J.

[305] Sanders TH, Weiss J, Hogewood L, Chen L, Paton C, McMahan RL, Sweatt JD (2019). Cognition-Enhancing Vagus Nerve Stimulation Alters the Epigenetic Landscape. J Neurosci.

[306] Reitz C, Lee JH, Rogers RS, Mayeux R (2011). Impact of genetic variation in SORCS1 on memory retention. PLoS ONE.

[307] Liang X, Slifer M, Martin ER, Schnetz-Boutaud N, Bartlett J, Anderson B, Zuchner S, Gwirtsman H, Gilbert JR, Pericak-Vance MA, Haines JL (2009). Genomic convergence to identify candidate genes for Alzheimer disease on chromosome 10. Hum Mutat.

[308] Schjeide BM, McQueen MB, Mullin K, DiVito J, Hogan MF, Parkinson M, Hooli B, Lange C, Blacker D, Tanzi RE, Bertram L (2009). Assessment of Alzheimer’s disease case-control associations using family-based methods. Neurogenetics.

[309] Laumet G, Chouraki V, Grenier-Boley B, Legry V, Heath S, Zelenika D, Fievet N, Hannequin D, Delepine M, Pasquier F (2010). Systematic analysis of candidate genes for Alzheimer’s disease in a French, genome-wide association study. J Alzheimers Dis.

[310] Xu W, Xu J, Wang Y, Tang H, Deng Y, Ren R, Wang G, Niu W, Ma J, Wu Y (2013). The genetic variation of SORCS1 is associated with late-onset Alzheimer’s disease in Chinese Han population. PLoS ONE.

[311] Bai Z, Stamova B, Xu H, Ander BP, Wang J, Jickling GC, Zhan X, Liu D, Han G, Jin LW (2014). Distinctive RNA expression profiles in blood associated with Alzheimer disease after accounting for white matter hyperintensities. Alzheimer Dis Assoc Disord.

[312] Printy BP, Verma N, Cowperthwaite MC, Markey MK (2014). Effects of genetic variation on the dynamics of neurodegeneration in Alzheimer’s disease. Ann Int Conf IEEE Eng Med Biol Soc.

[313] Wang HF, Yu JT, Zhang W, Wang W, Liu QY, Ma XY, Ding HM, Tan L (2012). SORCS1 and APOE polymorphisms interact to confer risk for late-onset Alzheimer’s disease in a Northern Han Chinese population. Brain Res.

[314] Park JH, Park I, Youm EM, Lee S, Park JH, Lee J, Lee DY, Byun MS, Lee JH, Yi D (2021). Novel Alzheimer’s disease risk variants identified based on whole-genome sequencing of APOE ε4 carriers. Transl Psychiatry.

[315] Brown CA, Schmidt C, Poulter M, Hummerich H, Klohn PC, Jat P, Mead S, Collinge J, Lloyd SE (2014). In vitro screen of prion disease susceptibility genes using the scrapie cell assay. Hum Mol Genet.

[316] Vieira MNN, Lima-Filho RAS, De Felice FG (2018). Connecting Alzheimer’s disease to diabetes: Underlying mechanisms and potential therapeutic targets. Neuropharmacology.

[317] Hao K, Di Narzo AF, Ho L, Luo W, Li S, Chen R, Li T, Dubner L, Pasinetti GM (2015). Shared genetic etiology underlying Alzheimer’s disease and type 2 diabetes. Mol Aspects Med.

[318] Janson J, Laedtke T, Parisi JE, O’Brien P, Petersen RC, Butler PC (2004). Increased risk of type 2 diabetes in Alzheimer disease. Diabetes.

[319] Talbot K, Wang HY, Kazi H, Han LY, Bakshi KP, Stucky A, Fuino RL, Kawaguchi KR, Samoyedny AJ, Wilson RS (2012). Demonstrated brain insulin resistance in Alzheimer’s disease patients is associated with IGF-1 resistance, IRS-1 dysregulation, and cognitive decline. J Clin Investig.

[320] Bomfim TR, Forny-Germano L, Sathler LB, Brito-Moreira J, Houzel JC, Decker H, Silverman MA, Kazi H, Melo HM, McClean PL (2012). An anti-diabetes agent protects the mouse brain from defective insulin signaling caused by Alzheimer’s disease- associated Abeta oligomers. J Clin Investig.

[321] Steen E, Terry BM, Rivera EJ, Cannon JL, Neely TR, Tavares R, Xu XJ, Wands JR, de la Monte SM (2005). Impaired insulin and insulin-like growth factor expression and signaling mechanisms in Alzheimer’s disease–is this type 3 diabetes?. J Alzheimers Dis.

[322] de la Monte SM, Tong M, Wands JR (2018). The 20-Year Voyage Aboard the Journal of Alzheimer’s Disease: Docking at ‘Type 3 Diabetes’, Environmental/Exposure Factors, Pathogenic Mechanisms, and Potential Treatments. J Alzheimers Dis.

[323] de la Monte SM, Wands JR (2005). Review of insulin and insulin-like growth factor expression, signaling, and malfunction in the central nervous system: relevance to Alzheimer’s disease. J Alzheimers Dis.

[324] Michailidis M, Moraitou D, Tata DA, Kalinderi K, Papamitsou T, Papaliagkas V (2022). Alzheimer’s Disease as Type 3 Diabetes: Common Pathophysiological Mechanisms between Alzheimer’s Disease and Type 2 Diabetes. Int J Mol Sci.

[325] Rorbach-Dolata A, Piwowar A (2019). Neurometabolic Evidence Supporting the Hypothesis of Increased Incidence of Type 3 Diabetes Mellitus in the 21st Century. Biomed Res Int.

[326] Kebede MA, Oler AT, Gregg T, Balloon AJ, Johnson A, Mitok K, Rabaglia M, Schueler K, Stapleton D, Thorstenson C (2014). SORCS1 is necessary for normal insulin secretory granule biogenesis in metabolically stressed beta cells. J Clin Investig.

[327] Clee SM, Yandell BS, Schueler KM, Rabaglia ME, Richards OC, Raines SM, Kabara EA, Klass DM, Mui ET, Stapleton DS (2006). Positional cloning of Sorcs1, a type 2 diabetes quantitative trait locus. Nat Genet.

[328] Goodarzi MO, Lehman DM, Taylor KD, Guo X, Cui J, Quinones MJ, Clee SM, Yandell BS, Blangero J, Hsueh WA (2007). SORCS1: a novel human type 2 diabetes susceptibility gene suggested by the mouse. Diabetes.

[329] Paterson AD, Waggott D, Boright AP, Hosseini SM, Shen E, Sylvestre MP, Wong I, Bharaj B, Cleary PA, Lachin JM (2010). A genome-wide association study identifies a novel major locus for glycemic control in type 1 diabetes, as measured by both A1C and glucose. Diabetes.

[330] Hertel JK, Johansson S, Raeder H, Platou CG, Midthjell K, Hveem K, Molven A, Njolstad PR (2011). Evaluation of four novel genetic variants affecting hemoglobin A1c levels in a population-based type 2 diabetes cohort (the HUNT2 study). BMC Med Genet.

[331] He Y, Fang Z, Yu G (2012). Sortilin-related VPS10 domain containing receptor 1 and Alzheimer’s disease-associated allelic variations preferentially exist in female or type 2 diabetes mellitus patients in southern Han Chinese. Psychogeriatrics.

[332] Lahiri DK, Maloney B. The “LEARn” (latent early-life associated regulation) model: an epigenetic pathway linking metabolic and cognitive disorders. J Alzheimers Dis. 2012;30(Suppl 2):S15-30. 10.3233/JAD-2012-120373 22555376

[333] Duits FH, Brinkmalm G, Teunissen CE, Brinkmalm A, Scheltens P, Van der Flier WM, Zetterberg H, Blennow K (2018). Synaptic proteins in CSF as potential novel biomarkers for prognosis in prodromal Alzheimer’s disease. Alzheimers Res Ther.

[334] Lleo A, Nunez-Llaves R, Alcolea D, Chiva C, Balateu-Panos D, Colom-Cadena M, Gomez-Giro G, Munoz L, Querol-Vilaseca M, Pegueroles J (2019). Changes in synaptic proteins precede neurodegeneration markers in preclinical Alzheimer’s disease cerebrospinal fluid. Mol Cell Proteomics.

[335] Rahman MM, Westermark GT, Zetterberg H, Hard T, Sandgren M (2018). Protofibrillar and Fibrillar Amyloid-beta Binding Proteins in Cerebrospinal Fluid. J Alzheimers Dis.

[336] Cataldo A, Rebeck GW, Ghetri B, Hulette C, Lippa C, Van Broeckhoven C, van Duijn C, Cras P, Bogdanovic N, Bird T (2001). Endocytic disturbances distinguish among subtypes of Alzheimer’s disease and related disorders. Ann Neurol.

[337] Brito-Moreira J, Lourenco MV, Oliveira MM, Ribeiro FC, Ledo JH, Diniz LP, Vital JFS, Magdesian MH, Melo HM, Barros-Aragao F (2017). Interaction of amyloid-beta (Abeta) oligomers with neurexin 2alpha and neuroligin 1 mediates synapse damage and memory loss in mice. J Biol Chem.

[338] Naito Y, Tanabe Y, Lee AK, Hamel E, Takahashi H (2017). Amyloid-beta Oligomers Interact with Neurexin and Diminish Neurexin-mediated Excitatory Presynaptic Organization. Sci Rep.

[339] Sindi IA, Tannenberg RK, Dodd PR (2014). Role for the neurexin-neuroligin complex in Alzheimer’s disease. Neurobiol Aging.

[340] Kreis A, Desloovere J, Suelves N, Pierrot N, Yerna X, Issa F, Schakman O, Gualdani R, de Clippele M, Tajeddine N (2021). Overexpression of wild-type human amyloid precursor protein alters GABAergic transmission. Sci Rep.

[341] Xu Y, Zhao M, Han Y, Zhang H (2020). GABAergic Inhibitory Interneuron Deficits in Alzheimer’s Disease: Implications for Treatment. Front Neurosci.

[342] Henley JM, Wilkinson KA (2016). Synaptic AMPA receptor composition in development, plasticity and disease. Nat Rev Neurosci.

[343] Boggild S, Molgaard S, Glerup S, Nyengaard JR (2018). Highly segregated localization of the functionally related vps10p receptors sortilin and SorCS2 during neurodevelopment. J Comp Neurol.

[344] Ma Q, Yang J, Milner TA, Vonsattel JG, Palko ME, Tessarollo L, Hempstead BL (2017). SorCS2-mediated NR2A trafficking regulates motor deficits in Huntington’s disease. JCI insight.

[345] Malik AR, Szydlowska K, Nizinska K, Asaro A, van Vliet EA, Popp O, Dittmar G, Fritsche-Guenther R, Kirwan JA, Nykjaer A (2019). SorCS2 Controls Functional Expression of Amino Acid Transporter EAAT3 and Protects Neurons from Oxidative Stress and Epilepsy-Induced Pathology. Cell Rep.

[346] Yang J, Ma Q, Dincheva I, Giza J, Jing D, Marinic T, Milner TA, Rajadhyaksha A, Lee FS, Hempstead BL (2021). SorCS2 is required for social memory and trafficking of the NMDA receptor. Mol Psychiatry.

[347] Gospodinova KO, Olsen D, Kaas M, Anderson SM, Phillips J, Walker RM, Bermingham ML, Payne AL, Giannopoulos P, Pandya D, Spires-Jones TL, Abbott CM, Porteous DJ, Glerup S, Evans KL. Loss of SORCS2 is Associated with Neuronal DNA Double-Strand Breaks. Cell Mol Neurobiol. 2021. 10.1007/s10571-021-01163-7. 10.1007/s10571-021-01163-7 PMC9813074 34741697

[348] Chaves G, Stanley J, Pourmand N (2019). Mutant Huntingtin Affects Diabetes and Alzheimer’s Markers in Human and Cell Models of Huntington’s Disease. Cells.

[349] Bothwell M, Giniger E (2000). Alzheimer’s disease: neurodevelopment converges with neurodegeneration. Cell.

[350] Batool S, Raza H, Zaidi J, Riaz S, Hasan S, Syed NI (2019). Synapse formation: from cellular and molecular mechanisms to neurodevelopmental and neurodegenerative disorders. J Neurophysiol.

[351] Olsen D, Wellner N, Kaas M, de Jong IEM, Sotty F, Didriksen M, Glerup S, Nykjaer A (2021). Altered dopaminergic firing pattern and novelty response underlie ADHD-like behavior of SorCS2-deficient mice. Transl Psychiatry.

[352] Vignoli B, Sansevero G, Sasi M, Rimondini R, Blum R, Bonaldo V, Biasini E, Santi S, Berardi N, Lu B, Canossa M (2021). Astrocytic microdomains from mouse cortex gain molecular control over long-term information storage and memory retention. Commun Biol.

[353] Mizui T, Ishikawa Y, Kumanogoh H, Lume M, Matsumoto T, Hara T, Yamawaki S, Takahashi M, Shiosaka S, Itami C (2015). BDNF pro-peptide actions facilitate hippocampal LTD and are altered by the common BDNF polymorphism Val66Met. Proc Natl Acad Sci USA.

[354] Breiderhoff T, Christiansen GB, Pallesen LT, Vaegter C, Nykjaer A, Holm MM, Glerup S, Willnow TE (2013). Sortilin-related receptor SORCS3 is a postsynaptic modulator of synaptic depression and fear extinction. PLoS ONE.

[355] Christiansen GB, Andersen KH, Riis S, Nykjaer A, Bolcho U, Jensen MS, Holm MM (2017). The sorting receptor SorCS3 is a stronger regulator of glutamate receptor functions compared to GABAergic mechanisms in the hippocampus. Hippocampus.

[356] Blue EE, Thornton TA, Kooperberg C, Liu S, Wactawski-Wende J, Manson J, Kuller L, Hayden K, Reiner AP (2021). Non-coding variants in MYH11, FZD3, and SORCS3 are associated with dementia in women. Alzheimers Dement.

[357] Wang M, Wang S, Li Y, Cai G, Cao M, Li L (2020). Integrated analysis and network pharmacology approaches to explore key genes of Xingnaojing for treatment of Alzheimer’s disease. Brain Behav.

[358] Cortes N, Andrade V, Maccioni RB (2018). Behavioral and Neuropsychiatric Disorders in Alzheimer’s Disease. J Alzheimers Dis.

[359] Novais F, Starkstein S (2015). Phenomenology of Depression in Alzheimer’s Disease. J Alzheimers Dis.

[360] Ni H, Xu M, Zhan GL, Fan Y, Zhou H, Jiang HY, Lu WH, Tan L, Zhang DF, Yao YG, Zhang C (2018). The GWAS Risk Genes for Depression May Be Actively Involved in Alzheimer’s Disease. J Alzheimers Dis.

[361] Liu J, Reggiani JDS, Laboulaye MA, Pandey S, Chen B, Rubenstein JLR, Krishnaswamy A, Sanes JR (2018). Tbr1 instructs laminar patterning of retinal ganglion cell dendrites. Nat Neurosci.

[362] Zhang Y, Li Y, Fan Y, Zhang X, Tang Z, Qi J, Zhao B, Li F, Chen X, Liang H (2022). SorCS3 promotes the internalization of p75(NTR) to inhibit GBM progression. Cell Death Dis.

[363] Sarlus H, Heneka MT (2017). Microglia in Alzheimer’s disease. J Clin Investig.

[364] Shi Y, Holtzman DM (2018). Interplay between innate immunity and Alzheimer disease: APOE and TREM2 in the spotlight. Nat Rev Immunol.

[365] Sampaio TB, Savall AS, Gutierrez MEZ, Pinton S (2017). Neurotrophic factors in Alzheimer’s and Parkinson’s diseases: implications for pathogenesis and therapy. Neural Regen Res.

[366] Li B, Gao Y, Zhang W, Xu JR (2018). Regulation and effects of neurotrophic factors after neural stem cell transplantation in a transgenic mouse model of Alzheimer disease. J Neurosci Res.

[367] Wu CC, Lien CC, Hou WH, Chiang PM, Tsai KJ (2016). Gain of BDNF Function in Engrafted Neural Stem Cells Promotes the Therapeutic Potential for Alzheimer’s Disease. Sci Rep.

[368] Choi SH, Bylykbashi E, Chatila ZK, Lee SW, Pulli B, Clemenson GD, Kim E, Rompala A, Oram MK, Asselin C (2018). Combined adult neurogenesis and BDNF mimic exercise effects on cognition in an Alzheimer’s mouse model. Science (New York, NY).

[369] de Pins B, Cifuentes-Diaz C, Thamila Farah A, Lopez-Molina L, Montalban E, Sancho-Balsells A, Lopez A, Gines S, Delgado-Garcia JM, Alberch J (2019). Conditional BDNF delivery from astrocytes rescues memory deficits, spine density and synaptic properties in the 5xFAD mouse model of Alzheimer disease. J Neurosci.

[370] Lee WC, Almeida S, Prudencio M, Caulfield TR, Zhang YJ, Tay WM, Bauer PO, Chew J, Sasaguri H, Jansen-West KR (2014). Targeted manipulation of the sortilin-progranulin axis rescues progranulin haploinsufficiency. Hum Mol Genet.

[371] A Phase 3 Study to Evaluate Efficacy and Safety of AL001 in Frontotemporal Dementia (INFRONT-3) https://clinicaltrials.gov/ct2/show/NCT04374136

